# Safety and immunogenicity of novel live attenuated type 1 and type 3 oral poliomyelitis vaccines in healthy adults in the USA: a first-in-human, observer-masked, multicentre, phase 1 randomised controlled trial

**DOI:** 10.1016/S1473-3099(25)00285-3

**Published:** 2025-12

**Authors:** Laina D Mercer, Arlene C Seña, E Ross Colgate, Jessica W Crothers, Peter F Wright, Mohamed Al-Ibrahim, Erman Tritama, Annelet Vincent, Bernardo A Mainou, Yiting Zhang, Jennifer Konopka-Anstadt, Ananda S Bandyopadhyay, Alan Fix, John O Konz, Chris Gast

**Affiliations:** a Center for Vaccine Innovation and Access, PATH, Seattle, WA, USA; b University of North Carolina at Chapel Hill, Institute for Global Health and Infectious Diseases, Chapel Hill, NC, USA; c The Vaccine Testing Center, Department of Microbiology and Molecular Genetics, University of Vermont Larner College of Medicine, Burlington, VT, USA; d Department of Pathology and Laboratory Medicine, University of Vermont Larner College of Medicine, Burlington, VT, USA; e Geisel School of Medicine, Dartmouth, Hanover, NH, USA; f Pharmaron CPC, Baltimore, MD, USA; g PT Bio Farma, Bandung, Indonesia; h Polio and Picornavirus Branch, Centers for Disease Control and Prevention, Atlanta, GA, USA; i Bill & Melinda Gates Foundation, Seattle, WA, USA

## Abstract

**Background:**

Reducing the risks of vaccine-derived polioviruses and vaccine-associated paralytic poliomyelitis motivated the development of novel types 1 and 3 oral poliovirus vaccines (nOPV1 and nOPV3, respectively), designed to have similar safety and immunogenicity and improved genetic stability (to reduce risk of reversion to neurovirulence) relative to types 1 or 3 Sabin-strain OPVs. We aimed to assess the safety and immunogenicity of nOPV1 and nOPV3 in healthy adults.

**Methods:**

We did a first-in-human, observer-masked, multicentre, phase 1 randomised controlled trial in healthy adults at four centres in the USA. Participants were block randomised, stratified by site and according to polio vaccination history (inactivated poliovirus vaccine [IPV] only [hereafter IPV participants] or regimens including OPV [hereafter OPV participants]), and randomly assigned to receive either nOPV or homotypic Sabin-strain monovalent OPV (mOPV). IPV participants received a single dose of nOPV1 or mOPV1 (cohort 1) or nOPV3 or mOPV3 (cohort 3) and OPV participants received two doses 28 days apart of nOPV1 or mOPV1 (cohort 2) or nOPV3 or mOPV3 (cohort 4). The primary outcome was safety among vaccinated participants. Secondary outcomes included homotypic serum neutralising antibody responses measured before and 28 days after each dose in a per-protocol population, and faecal viral shedding mainly in IPV participants assessed up to 56 days following each dose among vaccinated participants. This study was registered with ClinicalTrials.gov (NCT04529538) and is complete.

**Findings:**

Between May 6, 2021 and Feb 17, 2023, 377 individuals were assessed for eligibility, 226 were randomly assigned, and 205 receive at least one dose of nOPV1 (n=70), mOPV1 (n=45), nOPV3 (n=54), or mOPV3 (n=36). No serious adverse events were observed. Most adverse events were mild, severe events were rare, and solicited events were balanced across groups. Severe solicited events were predominantly fatigue, occurring in 1–4% of participants across all groups except for mOPV1 recipients in whom no such events were reported, and one case of nausea and vomiting in an mOPV1 recipient, and one case of abdominal pain in an nOPV1 recipient. Two (3%) participants in the nOPV1 groups (one reporting severe fatigue, headache, and myalgia, and one reporting abdominal pain) and one (2%) participant in the mOPV1 groups (kidney infection) reported a severe unsolicited adverse event within 28 days. Homotypic seroprotection was nearly 100% at baseline and was 100% 28 days after the first dose. Homotypic seroconversion rates after a single dose were high and similar for nOPV and mOPV (ranging from 86% to 100% for nOPV and from 86% to 93% for mOPV). Similar rates of viral shedding were observed among participants receiving nOPV or mOPV. Peak viral shedding rate detected via PCR among IPV participants was 100% on day 8 after a dose, across groups.

**Interpretation:**

nOPV1 and nOPV3 were well tolerated and showed similar immunogenicity and shedding profiles to mOPV1 and mOPV3, respectively, supporting progression of these vaccine candidates to phase 2 studies.

**Funding:**

Bill & Melinda Gates Foundation.

## Introduction

Since the Global Polio Eradication Initiative was established in 1988, the use of Sabin-strain oral poliovirus vaccines (OPVs) for immunisation has drastically reduced the burden of poliomyelitis and transmission of poliovirus and has made a major contribution to the eradication of types 2 and 3 wild polioviruses.^1^ These vaccines provide individual protection and interrupt person-to-person transmission of polioviruses. However, on extremely rare occasions, use of OPVs can result in vaccine-associated paralytic polio (VAPP) or paralytic outbreaks due to circulating vaccine-derived polioviruses (VDPVs).^2^ Central to both VAPP and circulating VDPV-induced disease is reversion of the vaccine strain to a more neurovirulent phenotype during intestinal replication of the virus. In the case of circulating VDPVs, reverted viruses can be transmitted to household or community contacts and cause disease outbreaks in settings of persistently poor immunisation coverage.

**Research in context**

**Evidence before this study**

A PubMed search to identify articles published from database inception to Oct 31, 2024, was done with the following terms: “oral poliovirus vaccine”, “reversion”, “genetic stability”, “circulating”, “meta-analysis”, “systematic review”, “randomized controlled trial”, “clinical trial”, “immunogenicity”, “safety”, “nOPV”, and “novel”. Sabin-strain circulating vaccine-derived polioviruses (VDPVs) and vaccine-associated paralytic polio (VAPP) now contribute a substantial proportion of paralytic poliomyelitis cases worldwide. To reduce the seeding of type 2 circulating VDPVs, a more genetically stable novel type 2 oral poliovirus vaccine (nOPV2) was developed to control outbreaks. WHO granted its use under emergency use listing in 2020 and prequalified the vaccine in 2023. More than one billion doses have been distributed since March, 2021, with surveillance data showing a promising safety and effectiveness profile. Sabin-strain types 1 and 3 polioviruses present similar risks for circulating VDPV and VAPP. In preclinical studies, chimeric viruses with the non-structural regions of nOPV2, including changes to the RNA sequence in the 5' untranslated region, the non-structural protein 2C, and the polymerase 3D, coupled with the coding region for the type-specific Sabin-strain capsid proteins, have shown similar immunogenicity and antigenicity, and lower neurovirulence compared with Sabin-strain viruses.

**Added value of this study**

This study is the first-in-human trial of novel type 1 and type 3 oral poliovirus vaccines (nOPV1 and nOPV3, respectively). It includes safety and immunogenicity data in adults with a history of either exclusive receipt of inactivated poliovirus vaccine (IPV) or exposure to OPV, permitting evaluation of nOPVs in both contexts, allowing a robust viral shedding evaluation in a subset of participants with no previous OPV exposure, and broadening demographic groups, given the timing of the IPV-to-OPV switch in the USA. We found that nOPV1 and nOPV3 were safe, well tolerated, and induced similar immunogenicity to their Sabin controls. The magnitude and duration of nOPV shedding was not higher than with Sabin controls. We also observed a reduction of shedding following the second dose, which is consistent with enhancement of intestinal immunity.

**Implications of all the available evidence**

The successful deployment of nOPV2 to combat type 2 circulating VDPVs suggested that use of such novel vaccines could be effective in the control of circulating VDPV outbreaks after the cessation of vaccines containing Sabin-strain types 1 and 3 polioviruses. nOPVs can thus support the Polio Endgame Strategy by providing outbreak response vaccines that are less likely to be associated with VAPP and seeding of new circulating VDPVs. The safety and immunogenicity evidence generated for nOPV1 and nOPV3 in this phase 1 clinical study were sufficient to justify the now ongoing phase 2 studies in geographically relevant target populations of previously vaccinated children and infants, as well as vaccine-naive neonates.

Due to the risks of VAPP and circulating VDPVs, the withdrawal of routine and supplementary immunisation activities with all OPVs, after the eradication of wild polioviruses, will be the final stage of polio eradication.^2^ The certification of eradication of type 2 wild poliovirus in 2015, and the predominance of type 2 polioviruses among circulating VDPVs, led to a coordinated global discontinuation of the routine use of Sabin-strain type 2 OPV (OPV2) in April, 2016. However, as the cohort of children without intestinal mucosal immunity against poliovirus type 2 grew following OPV2 cessation, and as inactivated poliovirus vaccine (IPV) coverage remained insufficient, suboptimal vaccination campaigns with Sabin-strain monovalent OPV2 (mOPV2) to control ongoing type 2 circulating VDPV outbreaks seeded new type 2 circulating VDPV outbreaks.^3^, ^4^

In parallel, novel OPV2 (nOPV2) was developed and introduced to reduce the risk of type 2 circulating VDPV outbreaks and VAPP cases. nOPV2 includes modifications to the Sabin 2 genome to stabilise domain V (the primary determinant of attenuation) and relocation of the cis-acting replication element and modifications to the polymerase to reduce recombination risk and enhance fidelity of replication. Phase 1 and 2 clinical trials have shown nOPV2 to be safe, immunogenic, and more genetically stable than the Sabin-strain OPV2.^5^, ^6^, ^7^ Based on these results, nOPV2 was deployed for outbreak control under WHO's emergency use listing, granted in November, 2020.^8^ Following completion of clinical development,^9^, ^10^ nOPV2 was prequalified by WHO in December, 2023.^11^ More than one billion doses of nOPV2 have now been deployed, and surveillance data are promising in terms of effectiveness and safety.^11^, ^12^, ^13^ The estimated emergence risk of type 2 circulating VDPVs has been substantially reduced, although not completely eliminated.^14^

Sabin-strain type 1 OPV (OPV1) and type 3 OPV (OPV3), like Sabin-strain OPV2, pose rare but potentially consequential risks for VAPP and VDPVs, necessitating development of genetically stable alternatives. Candidate viruses for both polioviruses type 1 and type 3 (novel OPV1 [nOPV1] and novel OPV3 [nOPV3], respectively) are chimeric viruses with nOPV2's non-structural regions coupled with Sabin-strain type 1 or type 3 structural proteins. Compared with the Sabin-strain OPVs, these novel candidates have similar pre-clinical immunogenicity and antigenicity, markedly lower neurovirulence in a transgenic mouse disease model, and reduced reversion to neurovirulence during cell-culture passaging.^15^ We aimed to evaluate the safety and immunogenicity of nOPV1 and nOPV3 in healthy adults.

## Methods

### Study design

We did a first-in-human, observer-masked, multicentre, phase 1 randomised controlled trial at the University of Vermont Vaccine Testing Center (Burlington, VT, USA), University of North Carolina Institute for Global Health and Infectious Diseases (Chapel Hill, NC, USA), Dartmouth-Hitchcock Medical Center (Lebanon, NH, USA), and Pharmaron (Baltimore, MD, USA).

The protocol (appendix pp 13–88) was approved by Advarra Institutional Review Board (Pro00045927) and the University of Vermont Institutional Review Board (STUDY00001242). Testing of serum and stool samples was reviewed by the US Centers for Disease Prevention and Control (CDC), and was done in accordance with applicable federal law and CDC policy.

### Participants

Participants were aged 18–45 years at enrolment and previously received either three or more doses of IPV and no OPV (cohorts 1 and 3; referred to as IPV participants) or a full primary poliovirus immunisation series containing OPV (cohorts 2 and 4; referred to as OPV participants). Previous vaccination status was ascertained through a combination of vaccination records, self-report, and participant age relative to the OPV-to-IPV switch dates. Eligible participants had serum neutralising antibody reciprocal titre ≥1:8 (seropositivity) for poliovirus type 1 (cohorts 1 and 2) or type 3 (cohorts 3 and 4), no clinically relevant medical conditions, and, if female, a negative serum pregnancy test. Participants were required to reside in the study area and not travel outside the USA until confirmation of cessation of vaccine virus shedding. Participants could not be breastfeeding or pregnant, and those of child-bearing potential agreed to practice adequate contraception for 30 days before the first study vaccination and for at least 90 days following the last study vaccination. Participants were excluded for conditions that might be adversely affected by participation, for medical conditions that might affect immune responses, if their occupation involved food handling, if they resided in a home with a septic tank, and if they had close contact with people who were immunosuppressed, pregnant, or who had not completed their primary polio immunisation series. All participants provided written informed consent.

### Randomisation and masking

The preferential enrolment of cohorts receiving type 1 vaccines first reflected the prioritisation of development of nOPV1, given the greater concern about type 1 wild polioviruses and type 1 circulating VDPVs than type 3 circulating VDPVs. IPV participants enrolled in cohort 1 were randomly assigned (1:1) to receive one dose of nOPV1 or Sabin-strain monovalent OPV1 (mOPV1), while OPV participants enrolled in cohort 2 were randomly assigned (2:1) to receive two doses of nOPV1 or mOPV1. After cohort 1 was fully enrolled, additional IPV participants were enrolled and randomly assigned (1:1) in cohort 3 to receive one dose of nOPV3 or Sabin-strain monovalent OPV3 (mOPV3). Similarly, after cohort 2 was fully enrolled, additional OPV participants were enrolled and randomly assigned (2:1) in cohort 4 to receive two doses of nOPV3 or mOPV3. IPV participants received one dose to permit viral shedding observation for 2 months after vaccination, whereas OPV participants provided safety data following each of the two doses before to support the evaluation of multidose trials in younger populations.

Block randomisation (size 2 for cohorts 1 and 3, size 3 for cohorts 2 and 4), stratified by study site, ensured balance within cohorts within each site, without a prespecified number enrolled at each site. The randomisation sequence was generated electronically and maintained by the Emmes Company (Rockville, MD, USA). Participants withdrawn before vaccination were replaced. The unmasked site pharmacists dispensed study vaccines into vials fitted with an overlay to mask contents, had no role in participant follow-up, and maintained vaccine allocation documentation in secure password-protected files. Unintentional unmasking was to be recorded as a protocol deviation.

### Procedures

Both control vaccines were Sabin-strain mOPVs, which are components of the WHO-prequalified bivalent OPV (bOPV) manufactured by Bio Farma (Bandung, Indonesia). The mOPV1 control vaccine contained at least 10^6.0^ 50% cell culture infective dose (CCID_50_) per 0·1 mL dose (lot number 2180120), and the mOPV3 control vaccine contained at least 10^5.8^ CCID_50_ per 0·1 mL dose (lot number 2080120), the standard dose-levels in bOPV.

Both nOPVs are live, attenuated polioviruses derived from a modified Sabin-strain type 2 infectious cDNA clone and propagated in Vero cells.^16^ Non-structural modifications based on nOPV2 were introduced in the viral nucleotide sequences in the 5' untranslated region (UTR) to improve and stabilise attenuation, with an element from the 2C coding region relocated to the 5ʹ UTR to safeguard loss of these modifications through single recombination. Additional modifications to the 3D polymerase reduce the frequency of mutation and recombination. Finally, the type 2 capsid-coding region of the genome was replaced with the capsid from Sabin-strain type 1 (for nOPV1) or Sabin-strain type 3 (for nOPV3) clones, resulting in chimeric viruses with nOPV2 non-structural regions and Sabin type 1 or 3 structural proteins. The nOPVs contained 10^6.5±0.5^ CCID_50_ per 0·1 mL dose (lot 2110120 for nOPV1 and lot 2310120 for nOPV3), reflecting the highest dose anticipated to be administered in a phase 2 study.

IPV participants in cohorts 1 and 3 received a single dose of vaccine on day 1. OPV participants in cohorts 2 and 4 received a dose on day 1 and on day 29. Safety events were reported from the time of informed consent signature through completion of the study 168 days after initial study vaccination (day 1). Each participant was observed for at least 30 min after vaccine administration for immediate adverse reactions. Data on solicited adverse events were collected on electronic participant memory aids for 7 days after each dose of study vaccine. If a solicited adverse event continued beyond 7 days, it was reported solely as a solicited adverse event. Data on unsolicited adverse events were collected for 28 days after each dose of study vaccine. Severity was assessed by the investigator based on the Division of AIDS table (version 2.1).^17^ Data on serious adverse events were collected from the initial study vaccination up to the end of the study participation (day 169).

Clinical laboratory assessments (complete blood count and blood chemistry) were done on days 1 and 8. Blood samples to evaluate vaccine safety were obtained and processed at the trial sites and transported to each sites' designated laboratory for testing. The severity of any abnormal clinical safety laboratory test results reported as adverse events were graded based on the US Food and Drug Administration toxicity grading scales for healthy volunteers in vaccine trials.^18^

For the assessment of humoral immunogenicity, serum samples were collected at the screening visit and on day 29 for all cohorts, and on day 57 for cohorts 2 and 4. All samples were aliquoted and stored at –20°C or lower before shipping to the Polio and Picornavirus Branch at the US CDC to measure type-specific polio neutralising antibodies.^19^ To establish eligibility (seropositivity for the serotype to be administered) before enrolment, an aliquot of the screening sample was evaluated in a similar assay at Quest Diagnostics (San Juan Capistrano, CA, USA).

Stool samples were collected by participants on days 1, 8, 15, 22, 29, 36, 43, 50, and 57 for all cohorts and additionally on days 3, 5, and 10 for cohorts 1 and 3 to detect and assess the quantity of vaccine virus shed, genetic stability of shed polioviruses, and to confirm cessation of shedding. Samples were processed at the trial sites and stored at –20°C or lower before being transported to the US CDC or Cerba Research (Rotterdam, Netherlands). The detection of type-specific poliovirus in stool was determined via multiplex real-time PCR at the US CDC. In samples positive solely for type 1 poliovirus in cohorts 1 and 2 or solely type 3 in cohorts 3 and 4, infectious virus was quantified as the CCID_50_ per g of stool at the US CDC. Next-generation sequencing (NGS) and neurovirulence of shed virus, as assessed by a transgenic mouse neurovirulence test, were performed by Cerba.

### Outcomes

The primary objective was to evaluate the safety and tolerability of nOPV1 and nOPV3 relative to active controls. Primary safety outcomes were the frequency of solicited adverse events for 7 days after each dose, the frequency of unsolicited adverse events for 28 days after each dose (including clinically significant laboratory values on day 8 reported as adverse events), and serious adverse events from day 1 up to study completion.

A secondary objective was to evaluate serum neutralising antibody titres elicited by nOPV1 and nOPV3 compared to type-specific Sabin controls. Secondary outcomes were type-specific neutralising antibodies measured at baseline and 28 days after vaccination, as summarised by medians and geometric mean titres (GMTs), as well as post-vaccination, type-specific, baseline neutralising antibody-adjusted GMT ratios and differences in proportions seroprotected (titre ≥1:8) and seroconversion (appendix p 7). Seroconversion was defined as at least a four-fold rise in titre in participants with titres above the lower limit of quantification (LLOQ; 2·5 log_2_) at baseline or at least a four-fold increase above the LLOQ (4·5 log_2_) for those with baseline titres at or below the LLOQ. Seroconversion was only assessed for participants with baseline titres at least 2·0 log_2_ below the upper limit of quantification (ULOQ; 10·5 log_2_), such that seroconversion could be observed.

An additional secondary objective was to assess faecal viral shedding in IPV participants (cohorts 1 and 3). Secondary outcomes were the proportion of participants positive for poliovirus at each stool collection via PCR and cell culture; the amount of vaccine virus in each stool sample (log_10_ CCID_50_ per g) and the mean of log_10_ CCID_50_ per g across post-vaccination collection timepoints (7 days, 14 days, 21 days, and 28 days after vaccination; the shedding index); and the area under the viral shedding curve. Additionally, time (days) to cessation of faecal shedding, defined as two consecutive PCR-negative specimens obtained at least 24 h apart, was a secondary outcome assessed in all participants.

Exploratory outcomes included additional viral shedding and immunogenicity assessments. Heterotypic immune responses (to viral serotypes other than that of the administered vaccine) were also assessed. Shedding outcomes included assessment in OPV participants (cohorts 2 and 4) of all secondary outcomes assessed for IPV participants (cohorts 1 and 3), as well as NGS and mouse neurovirulence in select stool samples from IPV participants to be reported elsewhere. Genetic stability data are outside the scope of this publication and will be reported elsewhere.

### Statistical analysis

The sample sizes for this study were primarily chosen to enable evaluation of safety in a sufficient number of adults to support a subsequent study in younger participants. We planned to enrol 40 IPV participants into each of cohorts 1 and 3, and 75 participants into each of cohorts 2 and 4. The planned samples sizes of cohorts 3 and 4 were subequently reduced in recognition of enrolment challenges and that these sample sizes would still support study objectives. With at least 50 participants receiving nOPV, there was a 95% probability of observing at least one adverse event if the event rate was 5·8% or higher, and the upper bound of the 95% CI if no events were observed would be 7·1%. Immunogenicity was a secondary objective and primarily descriptive in nature. Comparisons to the relevant controls were planned, but the sample size was not based on reaching a specific level of statistical power for these comparisons. Focusing on the OPV participants receiving nOPV, with at least 48 (cohort 2 assumption; detailed in the protocol) or 28 (cohort 4 assumption) participants receiving nOPV, if the true seroconversion rate was 50% (maximum variability), the confidence interval for the nOPV response rate would be expected to average ±15% or ±20% from the point estimate, respectively.

Dichotomous outcomes, including safety, seroconversion, and vaccine virus shedding positivity were summarised by counts, proportions, and exact 95% CIs. Fisher's exact test was used to compare rates of solicited adverse events between groups. The median and corresponding bootstrap-based 95% CI were calculated for log_2_ neutralising antibodies and for log_10_ CCID_50_ per g of shed virus. GMTs were estimated with likelihood-based methods to accommodate censoring at the assay LLOQ and ULOQ. GMT ratios were estimated via a linear model of log_2_ neutralising antibody titre as a function of group, site, and baseline log_2_ neutralising antibody and similarly accommodated censoring. Two-sided 95% CIs were computed by back-transformation of the confidence intervals derived from the model.

Safety and shedding outcomes were evaluated for all participants who received at least one dose of study vaccine (safety population). The per-protocol population for immunogenicity analyses was defined as all participants who correctly received study vaccinations per randomisation with no major protocol deviations determined to potentially interfere with the immunogenicity result of the participant. Protocol deviations were reviewed by the sponsor and site representatives and exclusions from the per-protocol population (at the timepoint level) were identified before unmasking.

### Role of the funding source

The funder of the study had no role in study design, data collection, data analysis, data interpretation, or writing of the report. ASB, who is employed by the funder, was involved in the design and reporting of the study.

## Results

**Figure 1:**
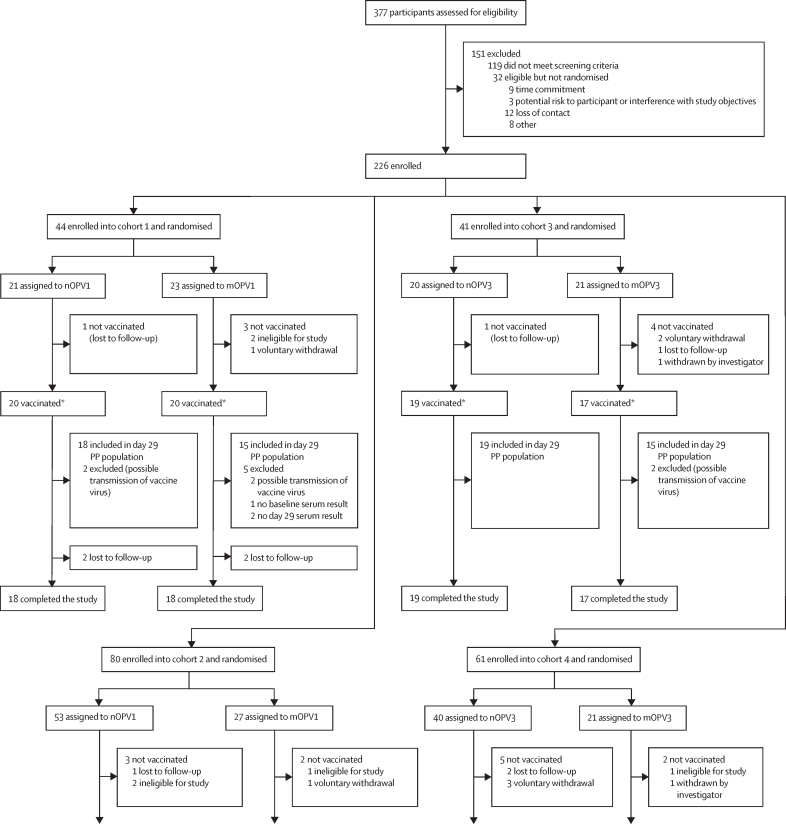

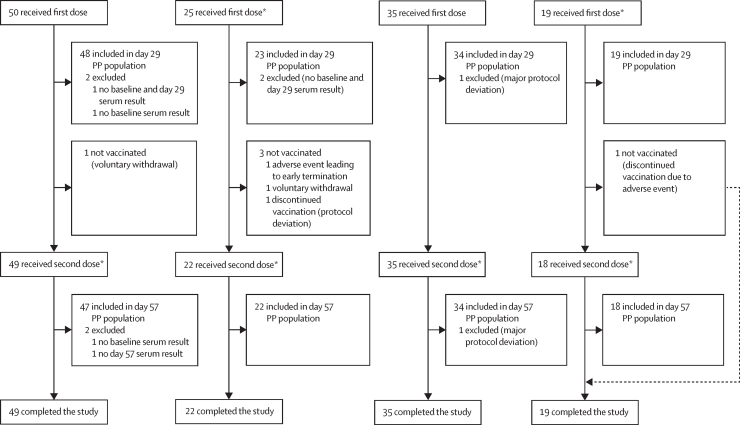
CONSORT diagram Enrolment into cohorts 3 and 4 began only after enrolment into cohorts 1 and 2 were completed, respectively. Individuals in cohorts 1 and 3 had a history of exposure to inactivated poliovirus vaccines only and received single doses of study vaccines. Individuals in cohorts 2 and 4 were previously exposed to OPV-containing regimens and received two doses of study vaccines. mOPV1=monovalent type 1 OPV. mOPV3=monovalent type 3 OPV. nOPV1=novel type 1 OPV. nOPV3=novel type 3 OPV. OPV=oral poliovirus vaccine. PP=per protocol. *Reactogenicity population for individual doses. In cohorts 2 and 4, the safety populations were identical to the reactogenicity populations. In cohort 1, the safety population comprised 18 participants each in the nOPV1 and mOPV1 groups because four participants (two in each group) were excluded due to possible transmission of vaccine virus. In cohort 3, the safety population was the same as the reactogenicity population in the nOPV3 group but included only 15 participants in the mOPV3 group as two participants were excluded due to possible transmission of vaccine virus. Potential vaccine virus transmission events were documented via sequencing of virus in stool.

Between May 6, 2021, and Feb 17, 2023, 377 participants were assessed for eligibility, of whom 119 were excluded for not meeting eligibility criteria and 32 were eligible but not randomised. Major reasons for ineligibility included inability to comply with study restrictions and procedures, presence of chronic or acute health conditions including out-of-range screening laboratory values, or close contact with immunosuppressed or incompletely vaccinated individuals. 226 participants were randomly assigned, 205 of whom received at least one dose of study vaccine (70 nOPV1, 45 mOPV1, 54 nOPV3, and 36 mOPV3; figure 1). These participants were included in the reactogenicity population for assessment of solicited adverse events. Six (8%) of 76 IPV participants who received at least one dose of study vaccine (3% [six of 205 receiving a first dose] of all participants), including two nOPV1 recipients and two mOPV1 recipients in cohort 1, and two mOPV3 recipients in cohort 3, were removed from the safety and per-protocol populations due to possible transmission of vaccine virus from an alternate study group (identified through NGS of stool samples [data not shown]; appendix p 2).

The mean age of participants in the safety population was 27·1 years (SD 7·4), and 110 (55%) of 199 participants were female. Of the 199 participants, 136 (68%) identified as White and 38 (19%) identified as Black or African American. The majority of Black or African American participants (29 [91%] of 32) were enrolled at a single site, exclusively into groups 3 and 4. Within cohorts, the nOPV and mOPV groups were balanced in terms of the distributions of sex, ethnicity, race, height, and weight (table 1).

**Table 1:** Study group demographics in the safety population

		**Cohort 1: IPV participants**		**Cohort 2: OPV participants**		**Cohort 3: IPV participants**		**Cohort 4: OPV participants**	
		nOPV1 (n=18)	mOPV1 (n=18)	nOPV1 (n=50)	mOPV1 (n=25)	nOPV3 (n=19)	mOPV3 (n=15)	nOPV3 (n=35)	mOPV3 (n=19)
Sex									
	Female	10 (55·6%)	9 (50·0%)	25 (50·0%)	15 (60·0%)	10 (52·6%)	7 (46·7%)	21 (60·0%)	13 (68·4%)
	Male	8 (44·4%)	9 (50·0%)	25 (50·0%)	10 (40·0%)	9 (47·4%)	8 (53·3%)	14 (40·0%)	6 (31·6%)
Ethnicity									
	Not Hispanic or Latino	18 (100%)	18 (100%)	44 (88·0%)	24 (96·0%)	17 (89·5%)	13 (86·7%)	33 (94·3%)	19 (100%)
	Hispanic or Latino	..	..	6 (12·0%)	..	2 (10·5%)	..	2 (5·7%)	..
	Unknown	..	..	..	1 (4·0%)	..	2 (13·3%)	..	..
Race									
	American Indian or Alaskan Native	..	..	..	..	..	1 (6·7%)	..	..
	Asian	2 (11·1%)	1 (5·6%)	5 (10·0%)	1 (4·0%)	1 (5·3%)	1 (6·7%)	3 (8·6%)	1 (5·3%)
	Native Hawaiian or Other Pacific Islander	..	..	..	..	..	..	..	..
	Black or African American	..	1 (5·6%)	3 (6·0%)	1 (4·0%)	5 (26·3%)	2 (13·3%)	17 (48·6%)	9 (47·4%)
	White	16 (88·9%)	16 (88·9%)	40 (80·0%)	22 (88·0%)	13 (68·4%)	9 (60·0%)	12 (34·3%)	8 (42·1%)
	Multiracial	..	..	2 (4·0%)	..	..	..	2 (5·7%)	1 (5·3%)
	Unknown	..	..	..	1 (4·0%)	..	2 (13·3%)	1 (2·9%)	..
Mean (SD) age, years		20·8 (2·6)	20·0 (1·6)	30·0 (6·3)	30·0 (6·5)	20·7 (2·1)	21·5 (2·4)	30·4 (6·6)	33·1 (8·8)
Median (range) age, years		21 (18–30)	20 (18–23)	29 (19–43)	28 (19–43)	20 (18–25)	21 (19–27)	30 (22–45)	31 (22–44)
Mean (SD) height, cm		170·8 (7·4)	171·6 (7·5)	172·9 (8·7)	169·8 (9·8)	173·4 (8·0)	173·1 (11·5)	171·0 (9·3)	169·3 (9·1)
Median (range) height, cm		170 (160–186)	174 (157–186)	173 (157–192)	168 (153–185)	174 (154–185)	172 (148–192)	169 (156–195)	168 (155–185)
Mean (SD) weight, kg		73·68 (15·24)	75·93 (15·88)	79·86 (16·24)	74·97 (14·51)	76·05 (18·62)	72·48 (14·83)	85·85 (21·14)	87·91 (21·44)
Median (range) weight, kg		71·6 (53·4–102·3)	73·0 (54·3–114·7)	78·8 (52·0–125·6)	80·2 (51·4–101·7)	68·4 (55·7–122·0)	73·3 (41·8–96·9)	80·9 (55·9–152·7)	81·2 (51·5–121·3)

Data are n (%), unless otherwise indicated. IPV=inactivated poliovirus vaccine. mOPV1=monovalent type 1 OPV. mOPV3=monovalent type 3 OPV. nOPV1=novel type 1 OPV. nOPV3=novel type 3 OPV. OPV=oral poliovirus vaccine.

Among participants receiving type 1 vaccines (cohorts 1 and 2), no participant reported a serious adverse event, and the proportion of participants in the safety population with any adverse event (solicited or unsolicited) was similar between the groups: 50 (74%) of 68 participants in the nOPV1 groups and 32 (74%) of 43 participants in the mOPV1 groups (table 2). In the reactogenicity population 40 (57%) of 70 participants in the nOPV1 groups and 31 (69%) of 45 participants in the mOPV1 groups reported solicited adverse events. Most events were mild, with few participants reporting severe events (one each with fatigue and abdominal pain in the nOPV1 groups, and one with severe nausea and vomiting in the mOPV1 groups). The frequencies of chills, joint aches or arthralgias, and vomiting were all significantly higher in the mOPV1 groups than the nOPV1 groups (p=0·023 for chills, p=0·017 for joint aches or arthralgias, and p=0·022 for vomiting). Chills were reported in three (4%) of 70 participants in the nOPV1 groups and eight (18%) of 45 participants in the mOPV1 groups, joint aches or arthralgias were reported in four (6%) participants in nOPV1 groups and ten (22%) participants in the mOPV1 groups, and vomiting was reported in no participant in the nOPV1 groups and in four (9%) participants in the mOPV1 groups (table 2). Results were generally similar when limited to within-cohort comparisons (appendix pp 10–11).

**Table 2:** Unsolicited and solicited adverse events after any dose in the safety population (unsolicited) and reactogenicity population (solicited)

		**Cohorts 1 & 2**		**Cohorts 3 & 4**	
		nOPV1	mOPV1	nOPV3	mOPV3
**Unsolicited adverse events**					
Number in safety population receiving any dose		68	43	54	34
Any serious adverse event throughout the study		0	0	0	0
Any adverse event, solicited or unsolicited		50 (74%)	32 (74%)	27 (50%)	20 (59%)
Any adverse event leading to withdrawal from the study		0	1 (2%)	0	0
Severe unsolicited adverse event within 28 days		2 (3%)	1 (2%)	0	0
Any unsolicited adverse event within 28 days		36 (53%)	18 (42%)	9 (17%)	5 (15%)
Any unsolicited adverse event assessed related to study vaccine within 28 days		5 (7%)	2 (5%)	0	0
**Solicited adverse events**					
Number of participants receiving any dose, reactogenicity population		70	45	54	36
Any solicited event					
	Severe	2 (3%)	1 (2%)	2 (4%)	1 (3%)
	Moderate	10 (14%)	11 (24%)	11 (20%)	5 (14%)
	Mild	28 (40%)	19 (42%)	12 (22%)	13 (36%)
	Any	40 (57%)	31 (69%)	25 (46%)	19 (53%)
Fever					
	Severe	0	0	0	0
	Moderate	0	0	0	0
	Mild	1 (1%)	0	0	1 (3%)
	Any	1 (1%)	0	0	1 (3%)
Chills					
	Severe	0	0	0	0
	Moderate	0	3 (7%)	0	0
	Mild	3 (4%)	5 (11%)	2 (4%)	1 (3%)
	Any*	3 (4%)	8 (18%)	2 (4%)	1 (3%)
Fatigue					
	Severe	1 (1%)	0	2 (4%)	1 (3%)
	Moderate	6 (9%)	8 (18%)	6 (11%)	3 (8%)
	Mild	18 (26%)	11 (24%)	8 (15%)	10 (28%)
	Any	25 (36%)	19 (42%)	16 (30%)	14 (39%)
Headache					
	Severe	0	0	0	0
	Moderate	2 (3%)	6 (13%)	4 (7%)	3 (8%)
	Mild	18 (26%)	11 (24%)	7 (13%)	7 (19%)
	Any	20 (29%)	17 (38%)	11 (20%)	10 (28%)
Muscle aches or myalgias					
	Severe	0	0	0	0
	Moderate	2 (3%)	4 (9%)	0	2 (6%)
	Mild	9 (13%)	7 (16%)	4 (7%)	2 (6%)
	Any	11 (16%)	11 (24%)	4 (7%)	4 (11%)
Joint aches or arthralgias					
	Severe	0	0	0	0
	Moderate	1 (1%)	1 (2%)	0	1 (3%)
	Mild	3 (4%)	9 (20%)	3 (6%)	2 (6%)
	Any*	4 (6%)	10 (22%)	3 (6%)	3 (8%)
Nausea					
	Severe	0	1 (2%)	0	0
	Moderate	0	3 (7%)	2 (4%)	0
	Mild	10 (14%)	1 (2%)	7 (13%)	2 (6%)
	Any	10 (14%)	5 (11%)	9 (17%)	2 (6%)
Vomiting					
	Severe	0	1 (2%)	0	0
	Moderate	0	2 (4%)	0	0
	Mild	0	1 (2%)	2 (4%)	0
	Any*	0	4 (9%)	2 (4%)	0
Abdominal pain					
	Severe	1 (1%)	0	0	0
	Moderate	2 (3%)	0	4 (7 %)	0
	Mild	8 (11%)	8 (18%)	7 (13%)	3 (8%)
	Any	11 (16%)	8 (18%)	11 (20%)	3 (8%)
Diarrhoea					
	Severe	0	0	0	0
	Moderate	1 (1%)	1 (2%)	1 (2%)	2 (6%)
	Mild	12 (17%)	11 (24%)	7 (13%)	9 (25%)
	Any	13 (19%)	12 (27%)	8 (15%)	11 (31%)

Data are n or n (%).

* mOPV1=monovalent type 1 OPV. mOPV3=monovalent type 3 OPV. nOPV1=novel type 1 OPV. nOPV3=novel type 3 OPV. OPV=oral poliovirus vaccine. p<0·05 from Fisher's Exact test for a difference in proportion of participants with a solicited adverse event between mOPV1 and nOPV1 vaccine groups.

In the safety population 36 (53%) of 68 participants in the nOPV1 groups and 18 (42%) of 43 participants in the mOPV1 groups reported an unsolicited adverse event within 28 days after vaccine administration, most of which were mild (table 2). The proportion of participants with any adverse event assessed by investigators as related to study vaccine was similar in the nOPV1 groups (five [7%] participants) and the mOPV1 groups (two [5%] participants); all were mild except one moderate paresthesia in an mOPV1 recipient. The proportion of participants with any severe adverse event was similar: two (3%) of 68 participants in the nOPV1 groups (one reporting severe fatigue, headache, and myalgia, and one reporting abdominal pain) and one (2%) of 43 participants in the mOPV1 groups (kidney infection) reported a severe unsolicited adverse event within 28 days. No participant in the nOPV1 groups and one (2%) participant in the mOPV1 groups reported an adverse event leading to withdrawal from the study (COVID-19).

Among participants receiving type 3 vaccines (cohorts 3 and 4), no participant reported a serious adverse event, and the proportion of participants in the safety population with any adverse event (solicited or unsolicited) was similar between the groups: 27 (50%) of 54 participants in the nOPV3 groups and 20 (59%) of 34 participants in the mOPV3 groups (table 2). None of the differences in the frequencies of solicited adverse events between the nOPV3 groups and the mOPV3 groups after any dose were statistically significant. In the reactogenicity population 25 (46%) of 54 participants in nOPV3 groups and 19 (53%) of 36 participants in mOPV3 groups reported solicited adverse events. Most events were mild or moderate, with only a few participants reporting severe events (two participants in the nOPV3 groups and one participant in the mOPV3 groups reported severe fatigue). The most frequently reported moderate events, by participant, were fatigue (six [11%] in the nOPV3 groups and three [8%] in the mOPV3 groups), headache (four [7%] in the nOPV3 groups and three [8%] in the mOPV3 groups), abdominal pain (four [7%] in the nOPV3 groups and none in the mOPV3 groups), and diarrhoea (one [2%] in the nOPV3 groups and two [6%] in the mOPV3 groups).

In the safety population**,** nine (17%) of 54 participants in the nOPV3 groups and five (15%) of 34 participants in the mOPV3 groups reported an unsolicited adverse event within 28 days after vaccine administration. No participant receiving nOPV3 or mOPV3 had any related adverse event, severe unsolicited adverse event within 28 days, or an adverse event leading to withdrawal.

Among the six participants who were excluded from the safety population after day 7 due to potential transmission of vaccine virus, one participant in cohort 1 and two in cohort 3 did not report any unsolicited adverse events. Unsolicited adverse events occurred in three participants: mild muscular weakness, peritonsillar abscess and lymphadenopathy, and moderate back pain and tonsillitis after nOPV1 (n=1); mild COVID-19 after nOPV1 (n=1); and mild viral infection after mOPV1 (n=1).

At baseline across all cohorts, the median neutralising antibody titres for all three serotypes were slightly lower in the nOPV groups relative to the mOPV groups, except for type 3 in cohort 3 in which baseline titres were similar (table 3; appendix p 3). In all cohorts, for both nOPV and mOPV, the greatest increases in type-specific neutralising antibody titres on day 28 after receiving a vaccine dose were homotypic to the vaccine administered, with median (log_2_) values reaching the ULOQ of 10·5 log_2_ by 28 days after dose 1 for type 1 (cohorts 1 and 2) and type 3 (cohorts 3 and 4). High titres were maintained for homotypic virus in all groups in cohorts 2 and 4 at 28 days following the second dose, but there was a slight decline in median titre for type 3 among mOPV3 recipients in cohort 4. While the eligibility screening at Quest showed all participants were seroprotected at baseline, seroprotection rates to poliovirus types 1 and 3 at baseline were less than 100% for all groups when serology testing was done at the US CDC laboratory (table 3). Strong homotypic responses were observed, with all groups achieving 100% seroprotection 28 days following the first dose.

**Table 3:** Summaries of homotypic type-specific neutralising antibody titres and seroprotection and seroconversion rates in the per-protocol population

	**Cohort 1: IPV participants**		**Cohort 2: OPV participants**		**Cohort 3: IPV participants**		**Cohort 4: OPV participants**	
	nOPV1	mOPV1	nOPV1	mOPV1	nOPV3	mOPV3	nOPV3	mOPV3
**Day 1, baseline**								
Number of participants	18	15	48	23	19	15	35	19
Median, log_2_	5·7 (5·3–7·4)	6·0 (5·3–7·3)	6·8 (5·2–8·6)	7·2 (5·3–8·3)	6·2 (5·7–8·3)	6·2 (5·5–7·2)	5·8 (3·8–8·0)	6·5 (5·2–8·5)
Seroprotection	15 (83%, 59–96)	14 (93%, 68–100)	45 (94%, 83–99)	20 (87%, 66–97)	18 (95%, 74–100)	14 (93%, 68–100)	31 (89%, 73–97)	18 (95%, 74–100)
**Day 29 (28 days after dose 1)**								
Number of participants	18	15	48	23	19	15	34	19
Median (log_2_)	≥10·5 (≥10·5–≥10·5)	≥10·5 (≥10·5–≥10·5)	≥10·5 (10·3–≥10·5)	≥10·5 (10·2–≥10·5)	≥10·5 (≥10·5–≥10·5)	≥10·5 (10·0–≥10·5)	≥10·5 (10·2–≥10·5)	≥10·5 (9·5–≥10·5)
Seroprotection	18 (100%, 81–100)	15 (100%, 78–100)	48 (100%, 93–100)	23 (100%, 85–100)	19 (100%, 82–100)	15 (100%, 78–100)	34 (100%, 90–100)	19 (100%, 82–100)
Seroconversion	16 (89%, 65–99)	13 (93%, 66–100)	31 (86%, 71–95)	16 (89%, 65–99)	15 (100%, 78–100)	12 (86%, 57–98)	27 (96%, 82–100)	13 (87%, 60–98)
**Day 57 (28 days after dose 2)**								
Number of participants	..	..	47	22	..	..	34	17
Median (log_2_)	..	..	≥10·5 (≥10·5–≥10·5)	≥10·5 (≥10·5–≥10·5)	..	..	≥10·5 (9·9–≥10·5)	9·8 (9·2–≥10·5)
Seroprotection	..	..	47 (100%, 92–100)	22 (100%, 85–100)	..	..	34 (100%, 90–100)	17 (100%, 80–100)
Seroconversion	..	..	34 (97%, 85–100)	16 (89%, 65–99)	..	..	27 (96%, 82–100)	11 (79%, 49–95)

Data are n, median (IQR), or n (%, 95% CI). The lower limit of quantitation is 2·5 log_2_ and the upper limit of quantitation is 10·5 log_2_. IPV=inactivated poliovirus vaccine. mOPV1=monovalent type 1 OPV. mOPV3=monovalent type 3 OPV. nOPV1=novel type 1 OPV. nOPV3=novel type 3 OPV. OPV=oral poliovirus vaccine.

**Figure 2:**
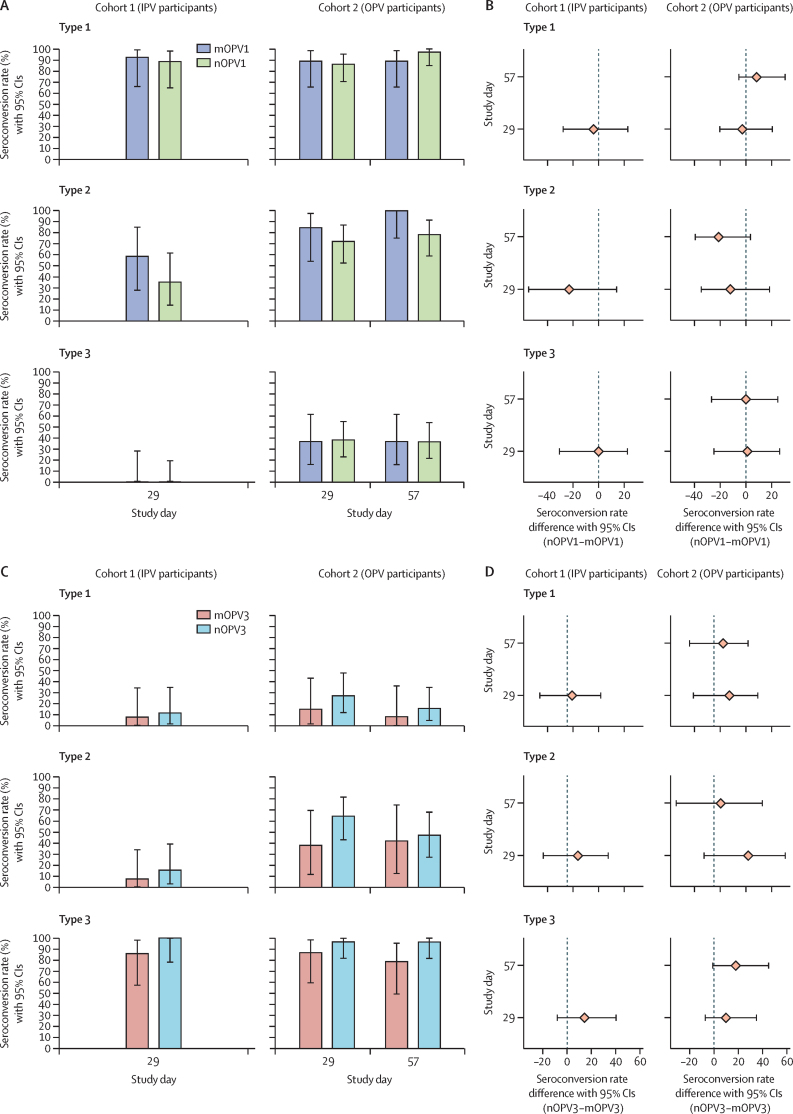
Seroconversion rates and differences in seroconversion rates with 95% CIs, by study day and serotype (A) Seroconversion rates for type 1 OPVs. (B) Difference in seroconversion rates for type 1 OPVs. (C) Seroconversion rates for type 3 OPVs. (D) Difference in seroconversion rates for type 3 OPVs. Individuals in cohorts 1 and 3 had a history of exposure to IPVs only and received single doses of study vaccines. Individuals in cohorts 2 and 4 were previously exposed to OPV-containing regimens and received two doses of study vaccines. IPV=inactivated poliovirus vaccine. mOPV1=monovalent type 1 OPV. nOPV1=novel type 1 OPV. mOPV3=monovalent type 3 OPV. nOPV3=novel type 3 OPV. OPV=oral poliovirus vaccine.

Seroconversion rates were high (>85%) in cohorts 1 and 2 (table 3; figure 2). Seroconversion after dose 2 was higher in the nOPV1 group (34 [97%] of 35) than the mOPV1 group (16 [89%] of 18). In cohorts 3 and 4, seroconversion rates were higher in the nOPV3 groups than in the mOPV3 groups after dose 1 and after dose 2 (cohort 4).

Substantial induction of heterotypic immunity against type 2 was observed in the nOPV1 and mOPV1 groups within cohort 2 (OPV participants), with median titres reaching the ULOQ 28 days after dose 1 (appendix p 3). A similar heterotypic response was also observed for type 2 among the participants receiving nOPV3 or mOPV3 in cohort 4 (OPV participants). Heterotypic responses to type 2 were observed to a lesser extent in cohort 1 (IPV participants receiving type 1 vaccine) and were not observed in cohort 3 (IPV participants receiving type 3 vaccine; figure 2). Heterotypic responses between types 1 and 3 were scarce among IPV participants and ranged from 7% to 27% following one or two doses among OPV participants.

**Figure 3:**
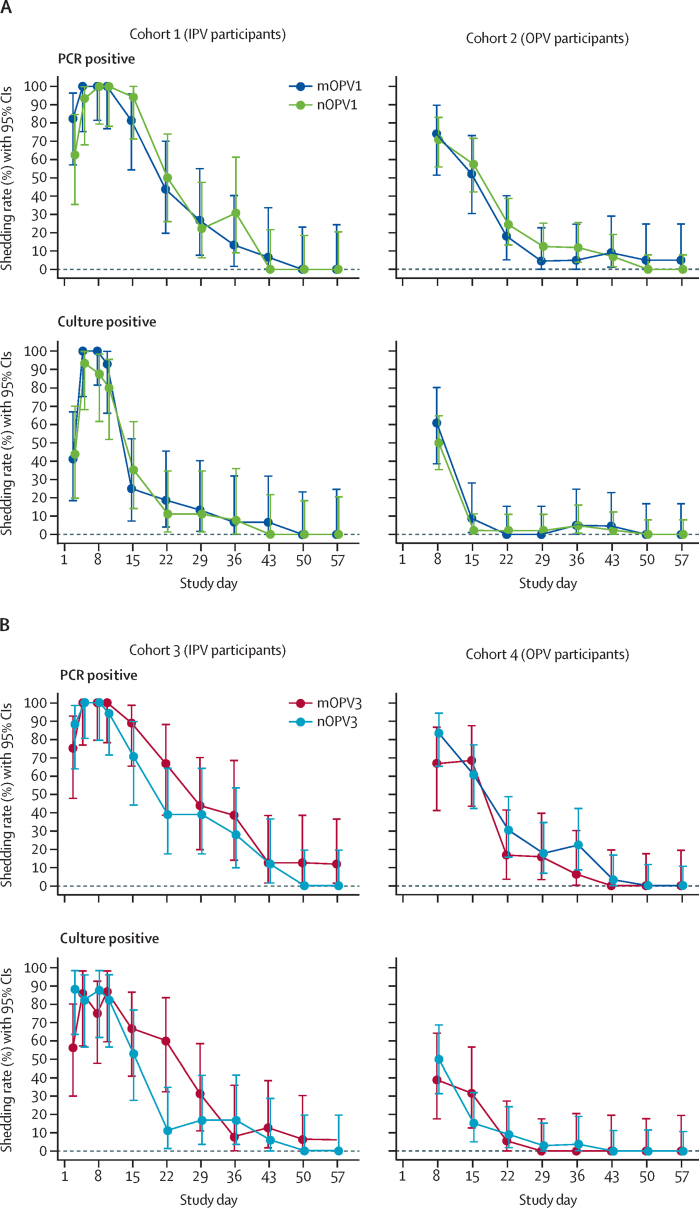
Shedding rates and 95% CIs for type 1 and type 3 vaccine viruses as measured by PCR and infectivity (safety population) (A) Type 1 vaccines. (B) Type 3 vaccines. IPV=inactivated poliovirus vaccine. mOPV1=monovalent type 1 OPV. nOPV1=novel type 1 OPV. mOPV3=monovalent type 3 OPV. nOPV3=novel type 3 OPV. OPV=oral poliovirus vaccine.

The rates of vaccine shedding as measured by PCR in IPV participants in both nOPV and mOPV groups (cohorts 1 and 3) were high (>70%) up to day 15 after vaccination, and, except for two mOPV3 recipient, all participants were PCR negative by 7 weeks (figure 3). For OPV participants, rates of viral shedding after dose 1 were more than 50% up to day 15 and less than 18% by day 29 (before dose 2). Viral shedding rates after dose 2 in all groups were very low, with the highest rates on day 36 (7 days after dose 2): five (12%) of 42 for nOPV1, one (5%) of 20 for mOPV1, six (22%) of 27 for nOPV3, and one (6%) of 16 for mOPV3. Viral infectivity results, as measured by log_10_ CCID_50_ per g, showed lower rates, but similar trends to vaccine shedding rates measured by PCR (figure 3). PCR results indicated that participants in cohorts 1 and 2 shed only poliovirus type 1 and participants in cohorts 3 and 4 shed only poliovirus type 3, except for the participants who were excluded due to potential transmission. As determined by PCR or infectivity, the daily rates and days until cessation of faecal shedding after dose 1 and dose 2 were not meaningfully different between nOPV1 and mOPV1 or between nOPV3 and mOPV3, in any cohort. Similarly, the shedding index and area under the curve, both measures of total excretion following each dose, were not statistically significantly different between groups within any cohort, following any dose (p>0·2).

## Discussion

This first-in-human trial of nOPV1 and nOPV3 showed that both vaccine candidates are safe and well tolerated in healthy adults in the USA and can elicit neutralising antibody responses similar to those elicited by mOPV1 and mOPV3, respectively. In addition, the profile of viral shedding was similar to that of the corresponding mOPV, with shedding in most vaccine recipients limited to the first 2 weeks after the first vaccination, with subsequent rapid decline to largely undetectable levels by 4–6 weeks after vaccination. These similar shedding profiles suggest that these chimeric nOPV viruses appear unlikely to be more transmissible.

Of importance for polio vaccines is provision of protective immunity as shown by type-specific serum neutralising antibody levels. Despite demonstration of homotypic seroprotection as an eligibility criterion through commercial laboratory testing, evaluation of immunogenicity outcomes by the CDC using a similar assay measured up to 17% of participants as being seronegative at baseline, depending on the study group. Differences between screening and CDC results at baseline are most likely attributable to minor assay differences. Following a single dose of the nOPV vaccines, all participants showed type-specific protective immunity. The next step for the advancement of these vaccine candidates is to show similar immunogenicity in geographically relevant target populations of young children and infants.

Detection of vaccine virus in stool provides evidence of successful intestinal infection and viral replication, which is crucial for generation of a robust immune response.^20^ Furthermore, reduced vaccine shedding following the second dose in OPV participants indicates successful induction of intestinal immunity, which is crucial to halt transmission in outbreak settings.^2^

In addition to robust homotypic serum neutralising antibody responses, strong heterotypic responses were observed to type 2 poliovirus by both nOPV and mOPV, with greater responses among those previously receiving OPV versus only IPV, and greater for type 1 than type 3 study vaccines. Such heterotypic responses have been reported in the past, including more frequent heterotypic responses among older age groups, and more frequent type 2 heterotypic responses, compared with types 1 and 3 heterotypic response.^21^ More recently, type 1 and 3 responses were observed among IPV-vaccinated children in Lithuania, following administration of type 2 OPV,^22^ whereas trivalent OPV-primed children in Pakistan showed type 2 responses following mOPV1, and enhanced heterotypic responses following bOPV vaccination.^23^ It is possible that heterotypic responses are short-lived, as has been observed with other OPV vaccines; however, this study does not provide longer-term timepoints necessary for such evaluations.

A limitation of this study is its limited sample size in an adult (non-target) population owing to its first-in-human status, and the limited generalisability of the results to the target paediatric population. In addition, limited observations of potential sample contamination or viral transmission were made from preplanned sequencing of virus identified in stool, which reduced the sample size for some endpoints (appendix p 2). Whereas strong evidence supporting a transmission hypothesis was observed for only one event, with evidence for the other events suggesting contamination during processing, a conservative classification of potential transmission was employed to segregate data for primary analyses. The highly sensitive tests employed, including NGS, could discriminate between the four vaccine viruses administered, although no test could have detected such an event within a vaccination group. Since such events were rarely observed between groups, we can assume incidence is similarly low within groups. Given this, and the limited replication evident after the second doses that were directly administered, such observations are not anticipated to have affected results reported here. Similar observations have been made in studies involving OPVs,^24^ and such events are likely to have occurred in other studies of replicating vaccines without detection.

The value of these candidate vaccines for deployment in the effort to eradicate polio depends not only on demonstration of similar safety and immunogenicity to their respective mOPVs, but also on demonstration of mucosal immunity for transmission blocking and greater genetic stability with less frequent reversion to neurovirulence. As was done for nOPV2,^24^, ^25^, ^26^, ^27^ analysis of NGS and transgenic mouse neurovirulence testing of faecally shed viruses is ongoing and will be reported elsewhere.

The safety and immunogenicity results of this study in healthy adult populations in the USA supported the initiation of currently ongoing phase 2 studies in relevant populations of young children and infants, including for nOPV1 alone (NCT05644184) and combinations of nOPV1, nOPV2, and nOPV3 (NCT06137664) in Bangladesh, and for nOPV3 in Panama (NCT05654467), with the aim of achieving licensure and WHO approval for use in outbreak responses.

### Contributors

### Data sharing

Data for this study will be made available to others in the scientific community upon request, in compliance with the principles of the Gates Foundation Global Access policy. For data access, please contact the corresponding author.

## Declaration of interests

ET is a full-time employee of the vaccine manufacturer, PT Bio Farma (Bandung, Indonesia). ASB is a full-time employee of the funder, Gates Foundation (Seattle, WA, USA). PFW has ongoing support from the Gates Foundation for the study of mucosal immunity in polio. JWC has received a career development grant from the National Institute of Infectious Disease and Allergy, National Institutes of Health (grant number K23AI175660). All other authors declare no competing interests.
