# Supplementary material for: Safety and immunogenicity of novel live attenuated type 1 and type 3 oral poliomyelitis vaccines in healthy adults in the USA: a first-in-human, observer-masked, multicentre, phase 1 randomised controlled trial
Source: Lancet Infect Dis. 2025 Dec;25(12):1363–76. doi: 10.1016/S1473-3099(25)00285-3 (PMC12630075; doi:10.1016/S1473-3099(25)00285-3)
Supplement: Supplementary appendix [file mmc1.pdf]

THE LANCET
Infectious Diseases

Supplementary appendix

This appendix formed part of the original submission and has been peer reviewed. We post it as supplied by the authors.

Supplement to: Mercer LD, Seña AC, Colgate ER, et al. Safety and immunogenicity of novel live attenuated type 1 and type 3 oral poliomyelitis vaccines in healthy adults in the USA: a first-in-human, observer-masked, multicentre, phase 1 randomised controlled trial. *Lancet Infect Dis* 2025; published online Aug 13. https://doi.org/10.1016/S1473-3099(25)00285-3.

Contents

Description of potential poliovirus transmission observed during the study.....2

S. Table 1: Summaries of heterotypic type-specific neutralizing antibody (NAb) titres measured at baseline and 28 days post each dose among the Per-Protocol Population. 3

S. Table 2: Heterotypic type-specific seroconversion rates measured by neutralizing antibody titres from baseline, in Per Protocol Population participants for which it is possible to observe seroconversion (log_2 titre $\leq 8 \cdot 5$ at baseline). 5

S. Figure 1: Geometric mean titres (GMTs) and GMT ratios with 95% CIs by study day and serotype. ...6

S. Figure 2: Seroprotection rates and differences in seroprotection rates with 95% CIs, by study day and serotype.....7

S. Table 3: Time (days) to cessation of fecal shedding in IPV participants (cohorts 1 and 3) in the Safety Population. 8

S. Table 4: Shedding Index Endpoint (SIE), by cohort, dose, and vaccine received in the Viral Shedding Population. 9

S. Table 5: Viral shedding Area Under the Curve (AUC), by cohort, dose, and vaccine received in the Viral Shedding Population. 10

S. Table 6: Summary of Solicited Adverse Events by Cohort, Group and Severity 11

Study Protocol…….13

Description of potential poliovirus transmission observed during the study

Due to the different study products in use and unique, intensive stool collection and testing performed in this study, an opportunity to observe potential OPV transmission events was created. This section summarizes the observations made in this study.

Potential transmission events were identified in six participants with IPV-only vaccination history (2 nOPV1 recipients and 2 mOPV1 recipients in cohort 1, and 2 mOPV3 recipients in cohort 3). These participants were excluded from the Safety and Per-protocol Populations (but not the Reactogenicity Population) due to the genomic detection (PCR and/or NGS testing) in at least one shed stool sample of the alternate vaccine used within the respective cohort. In one of the six cases, a mixture of both mOPV1 and nOPV1 was detected.

Following an investigation by the study team, including additional analysis of the impacted samples, two main hypotheses for the root cause were deemed most probable: 1) limited, inadvertent cross-contamination of stool samples during handling, processing, or testing of the impacted stool specimens, or 2) possible transmission events between study participants who were in close contact (e.g., roommates, romantic partners, teammates, close acquaintances, etc.) and had received opposing vaccines.

For five of the six excluded participants, only a single stool sample, aliquot, or derivative demonstrated the unanticipated alternate vaccine profile, suggesting inadvertent cross-contamination during stool handling or testing may have occurred. Notably, the extremely sensitive molecular techniques employed in this study could detect very low levels of cross-sample contamination between viruses. The study team utilized retesting of original primary samples, testing of backup secondary samples, retesting of derivative materials, and examination of longitudinal samples from the impacted participants to arrive at our conclusion of limited, inadvertent cross-contamination for these five samples. In addition to the results available from the type-specific poliovirus PCR assay, results from a nOPV-specific PCR assay were also utilized to rule out any further or more extensive contamination events. Additionally, robust internal PCR assay controls were in place and provided further reassurance that no extensive testing anomalies were observed.

For the remaining one of six excluded participants, a nOPV1 recipient, multiple longitudinal stool samples demonstrated the presence of mOPV1 or a mixture of both mOPV1 and nOPV1, suggesting sample contamination alone is an unlikely explanation. During investigation, this study participant reported sustained close contact with another study participant who was determined to have received the alternate study vaccine. As such, a transmission event was deemed to be the most probable root cause.

For all six participants, it remains difficult to fully rule out transmission as a possibility. Therefore, all six situations were conservatively treated as “potential transmission events” and all six participants were excluded from the safety and per-protocol populations.

S. Table 1: Summaries of heterotypic type-specific neutralizing antibody (NAb) titres measured at baseline and 28 days post each dose among the Per-Protocol Population.

	Cohort 1 IPV-background (Type 1)		Cohort 2 OPV-background (Type 1)		Cohort 3 IPV-background (Type 3)		Cohort 4 OPV-background (Type 3)	
	**nOPV1**	**mOPV1**	**nOPV1**	**mOPV1**	**nOPV3**	**mOPV3**	**nOPV3**	**mOPV3**
**Poliovirus neutralizing antibody titres**								
	**Type 3**				**Type 1**			
Day 1, baseline								
N	18	15	48	23	19	15	35	19
Median (log_2)	8·0	8·2	6·0	6·5	4·8	5·2	6·5	6·8
95% CI for Median	4·8, 8·5	5·5, 9·5	5·0, 7·5	3·5, 7·5	4·2, 7·2	3·8, 6·8	5·5, 8·5	5·2, 8·2
Seroprotection n (%)	14 (78%)	13 (87%)	44 (92%)	20 (87%)	18 (95%)	14 (93%)	33 (94%)	16 (84%)
95% CI for Seroprotection	52, 94	60, 98	80, 98	66, 97	74, 100	68, 100	81, 99	60, 97
Day 29 (28 days post-dose 1)								
N	18	15	48	23	19	15	34	19
Median (log_2)	7·3	7·5	8·5	8·2	6·2	5·5	7·0	7·2
95% CI for Median	5·2, 8·8	5·2, 9·8	7·5, 9·2	7·5, 9·5	4·5, 7·8	3·8, 6·8	6·2, 9·2	5·8, 8·5
Seroprotection n (%)	14 (78%)	13 (87%)	44 (92%)	22 (96%)	17 (90%)	15 (100%)	32 (94%)	17 (90%)
95% CI for Seroprotection	52, 94	60, 98	80, 98	78, 100	67, 99	78, 100	80, 99	67, 99
Day 57 (28 days post-dose 2)								
N	-	-	47	22	-	-	34	17
Median (log_2)	-	-	8·2	7·8	-	-	7·7	6·8
95% CI for Median	-	-	6·8, 8·5	6·2, 8·8	-	-	5·8, 9·2	4·8, 8·2
Seroprotection n (%)	-	-	45 (96%)	21 (96%)	-	-	31 (91%)	15 (88%)
95% CI for Seroprotection	-	-	85, 99	77, 100	-	-	76, 98	64, 99
	**Type 2**							
Day 1, baseline								
N	18	15	48	23	19	15	35	19
Median (log_2)	7·2	7·5	7·5	8·2	5·8	6·2	7·8	8·5
95% CI for Median	5·0, 7·7	5·5, 8·2	6·5, 9·0	6·2, 9·8	4·5, 7·5	3·8, 8·5	6·5, 8·8	6·5, 9·8
Seroprotection n (%)	15 (83%)	14 (93%)	48 (100%)	23 (100%)	19 (100%)	15 (100%)	32 (91%)	19 (100%)
95% CI for Seroprotection	59, 96	68, 100	93, 100	85, 100	82, 100	78, 100	77, 98	82, 100
Day 29 (28 days post-dose 1)								
N	18	15	48	23	19	15	34	19
Median (log_2)	7·8	8·8	≥10·5	≥10·5	5·8	6·2	10·2	10·2
95% CI for Median	6.8, 9.3	7.5, ≥10.5)	≥10.5, ≥10.5	≥10.5, ≥10.5	4.8, 8.5	3.5, 8.2	9.7, 10.2	8.2, ≥10.5
Seroprotection n (%)	17 (94%)	15 (100%)	48 (100%)	23 (100%)	19 (100%)	13 (87%)	33 (97%)	18 (95%)
95% CI for Seroprotection	73, 100	78, 100	93, 100	85, 100	82, 100	60, 98	85, 100	74, 100
Day 57 (28 days post-dose 2)								
N	-	-	47	22	-	-	34	17
Median (log_2)	-	-	≥10.5	≥10.5	-	-	9.7	9.5
95% CI for Median	-	-	10.2, ≥10.5	10.2, ≥10.5	-	-	8.5, 10.2	8.8, 10.2
Seroprotection n (%)	-	-	47 (100%)	22 (100%)	-	-	33 (97%)	17 (100%)

95% CI for Seroprotection	-	-	92, 100	85, 100	-	-	85, 100	80, 100

S. Table 2: Heterotypic type-specific seroconversion rates measured by neutralizing antibody titres from baseline, in Per Protocol Population participants for which it is possible to observe seroconversion (log_2 titre ≤8·5 at baseline).

	**Cohort 1 IPV-background (Type 1)**		**Cohort 2 OPV-background (Type 1)**		**Cohort 3 IPV-background (Type 3)**		**Cohort 4 OPV-background (Type 3)**	
	**nOPV1**	**mOPV1**	**nOPV1**	**mOPV1**	**nOPV3**	**mOPV3**	**nOPV3**	**mOPV3**
Seroconversion								
	**Type 3**				**Type 1**			
Day 29, post-dose 1								
N	14	9	39	19	18	14	26	14
n (%)	0 (0)	0 (0)	15 (39)	7 (37)	2 (11)	1 (7)	7 (27)	2 (14)
95% CI	(0, 23)	(0, 34)	(23, 55)	(16, 62)	(1, 35)	(0, 34)	(12, 48)	(2, 43)
N	-	-	38	19	-	-	26	14
n (%)	-	-	14 (37)	7 (37)	-	-	4 (15)	1 (7)
95% CI	-	-	(22, 54)	(16, 62)	-	-	(4, 35)	(0, 34)
Type 2								
Day 29, post-dose 1								
N	17	12	29	13	18	13	22	10
n (%)	6 (35)	7 (58)	21 (72)	11 (85)	3 (17)	1 (8)	15 (68)	4 (40)
95% CI	(14, 62)	(28, 85)	(53, 87)	(55, 98)	(4, 41)	(0, 36)	(45, 86)	(12, 74)
Day 57, post-dose 2								
N	-	-	28	13	-	-	22	10
n (%)	-	-	22 (79)	13 (100)	-	-	11 (50)	4 (40)
95% CI	-	-	(59, 92)	(75, 100)	-	-	(28, 72)	(12, 74)

S. Figure 1: Geometric mean titres (GMTs) and GMT ratios with 95% CIs by study day and serotype.

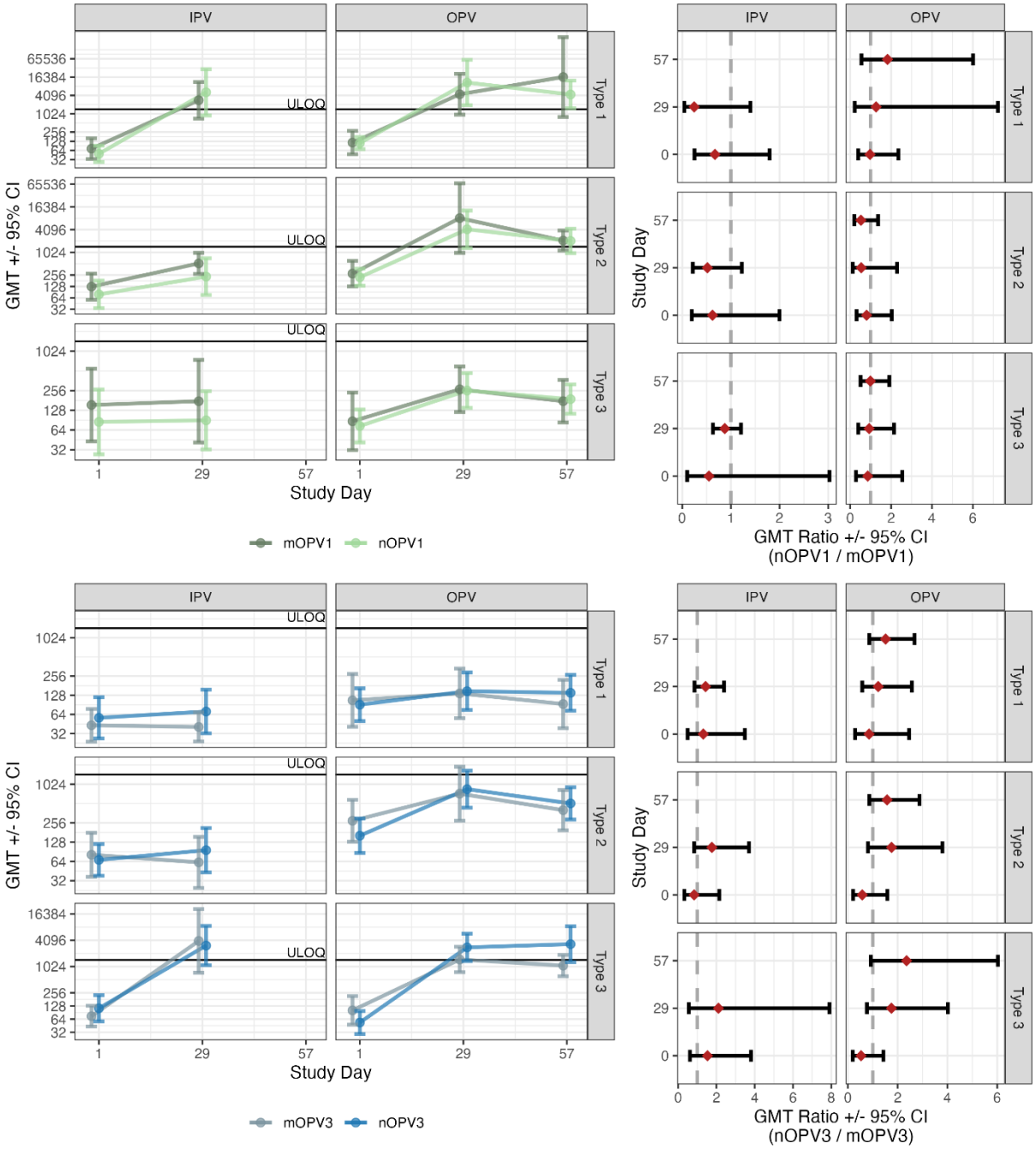

S. Figure 2: Seroprotection rates (SPRs) and differences in seroprotection rates with 95% CIs, by study day and serotype.

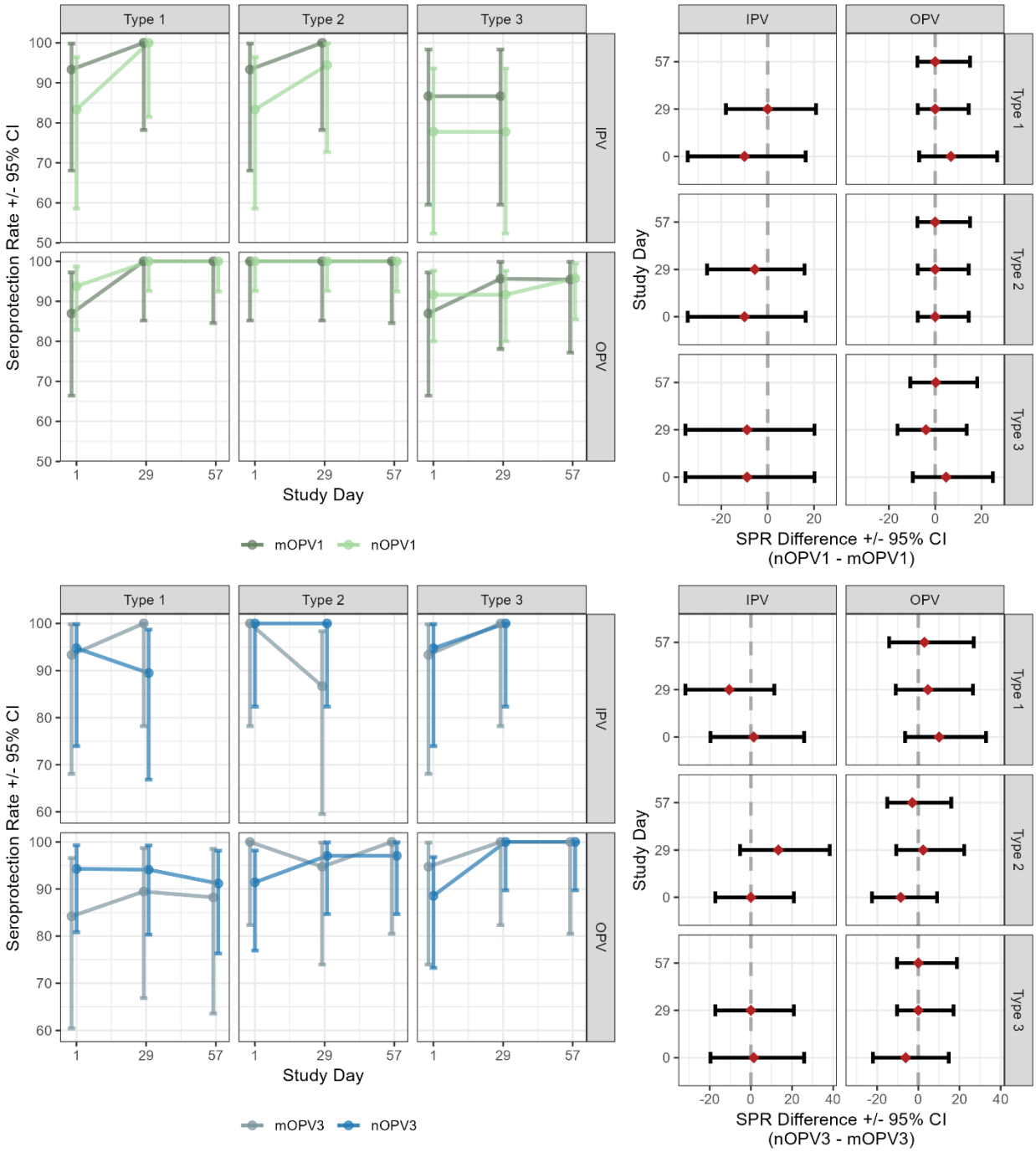

S. Table 3: Time (days) to cessation of fecal shedding in IPV participants (cohorts 1 and 3) in the Safety Population.

Method/ Cohort	Quartile	nOPV		mOPV		
		Estimate	95% CI	Estimate	95% CI [a]	P-value [b]
**PCR**						
Cohort 1 (Type 1)	25%	20	15 - 22	20	14 - 20	
	50% (Median)	22	15 - 24	20	20 - 29	0·975
	75%	29	22 - 42	29	20 - 43	
Cohort 3 (Type 3)	25%	20	10 - 20	22	13 - 29	
	50% (Median)	20	15 - 37	29	22 - 42	0·086
	75%	37	20 - 50	42	29 - 79	
**Culture (> 2·75 $\log_{10}$ $CCID_{50}$/g)**						
Cohort 1 (Type 1)	25%	10	8 - 15	13	9 - 13	
	50% (Median)	15	10 - 15	13	13 - 16	0·683
	75%	16	15 - 36	16	13 - 43	
Cohort 3 (Type 3)	25%	14	2 - 15	13	2 - 22	
	50% (Median)	15·5	14 - 20	25	13 - 35	0·220
	75%	20	15 - 42	35	22 - 43	
**Culture (≥ 4·0 $\log_{10}$ $CCID_{50}$/g)**						
Cohort 1 (Type 1)	25%	2	NC	9	2 - 11	
	50% (Median)	9	2 - 11	11	9 - 11	0·158
	75%	11	9 - 24	11	NC	
Cohort 3 (Type 3)	25%	5	2 - 8	6	2 - 9	
	50% (Median)	13	5 - 13	9	2 - 14	0·941
	75%	13	13 - 16	14	9 - 28	

NC = Not calculated
[a] 95% confidence interval calculated utilizing interval-censoring methodology.
[b] P-value calculated from the generalized log-rank test.

S. Table 4: Shedding Index Endpoint (SIE), by cohort, dose, and vaccine received in the Viral Shedding Population.

		SIE			Difference (nOPV – mOPV)		
**Cohort (Prior Vaccination)**	**Vaccination Group**	**n**	**Median**	**95% CI**	**Estimate**	**95% CI**	**P-Value**
**Post-Dose 1**							
Cohort 1 (IPV)	nOPV1	16	1·88	1·477, 2·344	-0·08	-0·828, 0·484	0·614
	mOPV1	13	1·96	1·734, 2·477			
Cohort 2 (OPV)	nOPV1	45	1·38	0·688, 1·484	-0·09	-0·992, 0·238	0·422
	mOPV1	17	1·46	0·719, 1·695			
Cohort 3 (IPV)	nOPV3	14	2·20	1·844, 3·016	-0·21	-1·484, 0·891	0·443
	mOPV3	11	2·41	1·766, 3·578			
Cohort 4 (OPV)	nOPV3	24	1·38	0·688, 1·539	-0·07	-0·930, 0·695	0·878
	mOPV3	16	1·44	0·688, 2·055			
**Post-Dose 2**							
Cohort 2 (OPV)	nOPV1	34	0·00	0·000, 0·000	0·00	0·000, 0·000	0·960
	mOPV1	19	0·00	0·000, 0·000			
Cohort 4 (OPV)	nOPV3	26	0·00	0·000, 0·000	0·00	0·000, 0·000	0·245
	mOPV3	13	0·00	0·000, 0·000			

N = Number of subjects vaccinated and with samples collected (delivered to the site) at all 4 time points.
95% CI = Bootstrap-based 95% confidence interval for the median and difference in medians (10,000 samples).
P-Value = Wilcoxon 2-sample test of a difference between groups.
SIE is calculated as the arithmetic mean of log10 CCID50/g across the appropriate nominal days or within respective sampling windows.
Log10 CCID50 per gram ≤LLOQ contributed a value equal to the LLOQ.

S. Table 5: Viral shedding Area Under the Curve (AUC), by cohort, dose, and vaccine received in the Viral Shedding Population.

		$\log_{10}$ AUC			Difference (nOPV – mOPV)		
**Cohort (Prior Vaccination)**	**Vaccination Group**	**n**	**Median**	**95% CI**	**Estimate**	**95% CI**	**P-Value**
**Post-Dose 1 Days 7 to 28**							
Cohort 2 (OPV)	nOPV1	46	27·30	9·625, 31·125	-2·05	-22·945, 17·359	0·689
	mOPV1	18	29·35	9·156, 35·625			
Cohort 4 (OPV)	nOPV3	26	27·09	9·625, 30·766	-5·28	-24·125, 11·000	0·566
	mOPV3	17	32·38	17·875, 38·500			
**Post-Dose 1 Days 2 to 28**							
Cohort 1 (IPV)	nOPV1	15	57·84	40·188, 66·625	-9·03	-33·688, 6·313	0·229
	mOPV1	15	66·88	52·797, 78·344			
Cohort 3 (IPV)	nOPV3	17	60·80	49·172, 80·438	-10·66	-32·406, 14·941	0·319
	mOPV3	12	71·46	56·711, 87·945			
**Post-Dose 1 Days 2 to 56**							
Cohort 1 (IPV)	nOPV1	14	58·43	42·086, 66·625	-6·20	-44·797, 14·398	0·482
	mOPV1	13	64·63	51·531, 96·359			
Cohort 3 (IPV)	nOPV3	16	72·37	49·359, 100·734	-17·77	-61·602, 30·969	0·501
	mOPV3	12	90·14	56·711, 122·156			
**Post-Dose-2 Days 28 to 56**							
Cohort 2 (OPV)	nOPV1	40	0·00	0·000, 0·000	0·00	0·000, 0·000	0·833
	mOPV1	20	0·00	0·000, 0·000			
Cohort 4 (OPV)	nOPV3	28	0·00	0·000, 0·000	0·00	0·000, 0·000	0·227
	mOPV3	15	0·00	0·000, 0·000			

N = Number of subjects in the safety population.
95% CI = Bootstrap-based 95% confidence interval for the median and difference in medians (10,000 samples).
P-Value = Wilcoxon 2-sample test of a difference between groups.
Log10 CCID50 per gram ≤LLOQ contributed a value equal to the LLOQ.

S. Table 6: Summary of Solicited Adverse Events by Cohort, Group and Severity

		Participants	Severe	Moderate	Mild	None
Reaction	Vaccine	N	n (%)	n (%)	n (%)	n (%)
**Cohort 1**						
Any Solicited Event	nOPV1	20	1 (5.0)	3 (15.0)	7 (35.0)	9 (45.0)
	mOPV1	20	0 (0.0)	4 (20.0)	8 (40.0)	8 (40.0)
Fever	nOPV1	20	0 (0.0)	0 (0.0)	0 (0.0)	20 (100)
	mOPV1	20	0 (0.0)	0 (0.0)	0 (0.0)	20 (100)
Chills	nOPV1	20	0 (0.0)	0 (0.0)	1 (5.0)	19 (95.0)
	mOPV1	20	0 (0.0)	2 (10.0)	3 (15.0)	15 (75.0)
Fatigue	nOPV1	20	1 (5.0)	2 (10.0)	2 (10.0)	15 (75.0)
	mOPV1	20	0 (0.0)	4 (20.0)	4 (20.0)	12 (60.0)
Headache	nOPV1	20	0 (0.0)	0 (0.0)	5 (25.0)	15 (75.0)
	mOPV1	20	0 (0.0)	2 (10.0)	5 (25.0)	13 (65.0)
Muscle Aches/Myalgias	nOPV1	20	0 (0.0)	1 (5.0)	1 (5.0)	18 (90.0)
	mOPV1	20	0 (0.0)	1 (5.0)	5 (25.0)	14 (70.0)
Joint Aches/Arthralgias	nOPV1	20	0 (0.0)	1 (5.0)	0 (0.0)	19 (95.0)
	mOPV1	20	0 (0.0)	0 (0.0)	4 (20.0)	16 (80.0)
Nausea	nOPV1	20	0 (0.0)	0 (0.0)	2 (10.0)	18 (90.0)
	mOPV1	20	0 (0.0)	2 (10.0)	0 (0.0)	18 (90.0)
Vomiting	nOPV1	20	0 (0.0)	0 (0.0)	0 (0.0)	20 (100)
	mOPV1	20	0 (0.0)	2 (10.0)	0 (0.0)	18 (90.0)
Abdominal pain	nOPV1	20	0 (0.0)	1 (5.0)	4 (20.0)	15 (75.0)
	mOPV1	20	0 (0.0)	0 (0.0)	5 (25.0)	15 (75.0)
Diarrhea	nOPV1	20	0 (0.0)	0 (0.0)	2 (10.0)	18 (90.0)
	mOPV1	20	0 (0.0)	0 (0.0)	4 (20.0)	16 (80.0)
**Cohort 2: Post-Any Dose**						
Any Solicited Event	nOPV1	50	1 (2.0)	7 (14.0)	21 (42.0)	21 (42.0)
	mOPV1	25	1 (4.0)	7 (28.0)	11 (44.0)	6 (24.0)
Fever	nOPV1	50	0 (0.0)	0 (0.0)	1 (2.0)	49 (98.0)
	mOPV1	25	0 (0.0)	0 (0.0)	0 (0.0)	25 (100)
Chills	nOPV1	50	0 (0.0)	0 (0.0)	2 (4.0)	48 (96.0)
	mOPV1	25	0 (0.0)	1 (4.0)	2 (8.0)	22 (88.0)
Fatigue	nOPV1	50	0 (0.0)	4 (8.0)	16 (32.0)	30 (60.0)
	mOPV1	25	0 (0.0)	4 (16.0)	7 (28.0)	14 (56.0)
Headache	nOPV1	50	0 (0.0)	2 (4.0)	13 (26.0)	35 (70.0)
	mOPV1	25	0 (0.0)	4 (16.0)	6 (24.0)	15 (60.0)
Muscle Aches/Myalgias	nOPV1	50	0 (0.0)	1 (2.0)	8 (16.0)	41 (82.0)
	mOPV1	25	0 (0.0)	3 (12.0)	2 (8.0)	20 (80.0)
Joint Aches/Arthralgias	nOPV1	50	0 (0.0)	0 (0.0)	3 (6.0)	47 (94.0)
	mOPV1	25	0 (0.0)	1 (4.0)	5 (20.0)	19 (76.0)
Nausea	nOPV1	50	0 (0.0)	0 (0.0)	8 (16.0)	42 (84.0)
	mOPV1	25	1 (4.0)	1 (4.0)	1 (4.0)	22 (88.0)
Vomiting	nOPV1	50	0 (0.0)	0 (0.0)	0 (0.0)	50 (100)
	mOPV1	25	1 (4.0)	0 (0.0)	1 (4.0)	23 (92.0)
Abdominal pain	nOPV1	50	1 (2.0)	1 (2.0)	4 (8.0)	44 (88.0)
	mOPV1	25	0 (0.0)	0 (0.0)	3 (12.0)	22 (88.0)
Diarrhea	nOPV1	50	0 (0.0)	1 (2.0)	10 (20.0)	39 (78.0)
	mOPV1	25	0 (0.0)	1 (4.0)	7 (28.0)	17 (68.0)
**Cohort 3**						
Any Solicited Event	nOPV3	19	1 (5.3)	4 (21.1)	5 (26.3)	9 (47.4)
	mOPV3	17	0 (0.0)	2 (11.8)	7 (41.2)	8 (47.1)
Fever	nOPV3	19	0 (0.0)	0 (0.0)	0 (0.0)	19 (100)
	mOPV3	17	0 (0.0)	0 (0.0)	0 (0.0)	17 (100)
Chills	nOPV3	19	0 (0.0)	0 (0.0)	1 (5.3)	18 (94.7)
	mOPV3	17	0 (0.0)	0 (0.0)	0 (0.0)	17 (100)
Fatigue	nOPV3	19	1 (5.3)	1 (5.3)	5 (26.3)	12 (63.2)
	mOPV3	17	0 (0.0)	1 (5.9)	6 (35.3)	10 (58.8)
Headache	nOPV3	19	0 (0.0)	2 (10.5)	1 (5.3)	16 (84.2)
	mOPV3	17	0 (0.0)	0 (0.0)	5 (29.4)	12 (70.6)
Muscle Aches/Myalgias	nOPV3	19	0 (0.0)	0 (0.0)	2 (10.5)	17 (89.5)
	mOPV3	17	0 (0.0)	1 (5.9)	1 (5.9)	15 (88.2)
Joint Aches/Arthralgias	nOPV3	19	0 (0.0)	0 (0.0)	1 (5.3)	18 (94.7)
	mOPV3	17	0 (0.0)	0 (0.0)	2 (11.8)	15 (88.2)
Nausea	nOPV3	19	0 (0.0)	0 (0.0)	1 (5.3)	18 (94.7)

		Participants	Severe	Moderate	Mild	None
Reaction	Vaccine	N	n (%)	n (%)	n (%)	n (%)
	mOPV3	17	0 (0.0)	0 (0.0)	1 (5.9)	16 (94.1)
Vomiting	nOPV3	19	0 (0.0)	0 (0.0)	1 (5.3)	18 (94.7)
	mOPV3	17	0 (0.0)	0 (0.0)	0 (0.0)	17 (100)
Abdominal pain	nOPV3	19	0 (0.0)	3 (15.8)	3 (15.8)	13 (68.4)
	mOPV3	17	0 (0.0)	0 (0.0)	1 (5.9)	16 (94.1)
Diarrhea	nOPV3	19	0 (0.0)	0 (0.0)	3 (15.8)	16 (84.2)
	mOPV3	17	0 (0.0)	1 (5.9)	5 (29.4)	11 (64.7)
**Cohort 4: Post-Any Dose**						
Any Solicited Event	nOPV3	35	1 (2.9)	7 (20.0)	7 (20.0)	20 (57.1)
	mOPV3	19	1 (5.3)	3 (15.8)	6 (31.6)	9 (47.4)
Fever	nOPV3	35	0 (0.0)	0 (0.0)	0 (0.0)	35 (100)
	mOPV3	19	0 (0.0)	0 (0.0)	1 (5.3)	18 (94.7)
Chills	nOPV3	35	0 (0.0)	0 (0.0)	1 (2.9)	34 (97.1)
	mOPV3	19	0 (0.0)	0 (0.0)	1 (5.3)	18 (94.7)
Fatigue	nOPV3	35	1 (2.9)	5 (14.3)	3 (8.6)	26 (74.3)
	mOPV3	19	1 (5.3)	2 (10.5)	4 (21.1)	12 (63.2)
Headache	nOPV3	35	0 (0.0)	2 (5.7)	6 (17.1)	27 (77.1)
	mOPV3	19	0 (0.0)	3 (15.8)	2 (10.5)	14 (73.7)
Muscle Aches/Myalgias	nOPV3	35	0 (0.0)	0 (0.0)	2 (5.7)	33 (94.3)
	mOPV3	19	0 (0.0)	1 (5.3)	1 (5.3)	17 (89.5)
Joint Aches/Arthralgias	nOPV3	35	0 (0.0)	0 (0.0)	2 (5.7)	33 (94.3)
	mOPV3	19	0 (0.0)	1 (5.3)	0 (0.0)	18 (94.7)
Nausea	nOPV3	35	0 (0.0)	2 (5.7)	6 (17.1)	27 (77.1)
	mOPV3	19	0 (0.0)	0 (0.0)	1 (5.3)	18 (94.7)
Vomiting	nOPV3	35	0 (0.0)	0 (0.0)	1 (2.9)	34 (97.1)
	mOPV3	19	0 (0.0)	0 (0.0)	0 (0.0)	19 (100)
Abdominal pain	nOPV3	35	0 (0.0)	1 (2.9)	4 (11.4)	30 (85.7)
	mOPV3	19	0 (0.0)	0 (0.0)	2 (10.5)	17 (89.5)
Diarrhea	nOPV3	35	0 (0.0)	1 (2.9)	4 (11.4)	30 (85.7)
	mOPV3	19	0 (0.0)	1 (5.3)	4 (21.1)	14 (73.7)

N= Number of Subjects in the safety population who received the specified dose. n = number of subjects with at least one event.
Cells display number (%) of subjects with at least one event at the specified severity. The maximum severity reported is used for summaries across events (Any Solicited Event).

Protocol Number

CVIA 076

Protocol Title

A first-in-human, phase 1, randomized, observer-blind, controlled study to assess the safety and immunogenicity of novel live attenuated type 1 and type 3 oral poliomyelitis vaccines in healthy adults

Sponsor

PATH

Collaborating Partner & Pharmaceutical Support

[redacted]

Source of funding

[redacted]

Protocol Version Number & Date

Version 5.0

17 February 2022

NCT ID: NCT04529538

Confidentiality Statement

This document is confidential and is to be distributed for review only to investigators, potential investigators, consultants, study staff, and applicable independent ethics committees or institutional review boards. The contents of this document shall not be disclosed to others without written authorization from PATH (or others, as applicable), unless it is necessary to obtain informed consent from potential study participants.

CONFIDENTIAL

TABLE OF CONTENTS

Title Page 1
TABLE OF CONTENTS 2
LIST OF TABLES 5
LIST OF FIGURES 6
ABBREVIATIONS AND ACRONYMS 7
INVESTIGATOR'S AGREEMENT 11
KEY ROLES AND CONTACT INFORMATION 12
PROTOCOL SUMMARY 14
1 BACKGROUND AND RATIONALE 20
1.1 Burden of Disease 20
1.2 Pathogen 21
1.3 Description of Study Vaccine 21
1.4 Summary of Nonclinical Studies of Study Vaccine 23
1.4.1 Growth and yield in Vero cells 24
1.4.2 Antigenicity 25
1.4.3 Immunogenicity 25
1.4.4 Attenuation phenotypes 26
1.5 Summary of Clinical Studies of Study Vaccine 26
1.5.1 Safety 27
1.5.2 Immunogenicity 28
1.5.3 Viral Shedding 28
1.5.4 Genetic Stability 28
1.6 Overall Development Strategy 28
2 HYPOTHESIS, OBJECTIVES AND ENDPOINTS 28
2.1 Study Hypotheses 28
2.2 Study Objectives 29
2.2.1 Primary Objective: 29
2.2.2 Secondary Objectives: 29
2.2.3 Exploratory Objectives: 29
2.3 Study Endpoints 29
2.3.1 Primary Endpoints: 29
2.3.2 Secondary Endpoints: 30
2.3.3 Exploratory Endpoints: 30
3 STUDY DESIGN 31
4 STUDY POPULATION 32

4.1 Description of Study Population 32
4.2 Inclusion Criteria 32
4.3 Exclusion Criteria 33

5 STUDY PRODUCTS 34
5.1 Study Vaccine 34

5.1.1 Product Description 34
5.1.2 Manufacturer 35
5.1.3 Presentation and Formulation 35
5.1.4 Stability and Storage 35

5.2 Dose Preparation and Administration 36
5.3 Accountability and Disposal 36

6 STUDY PROCEDURES 36
6.1 Recruitment 36
6.2 Study Visits 36

6.2.1 Screening – Day -90 to Day 1 36
6.2.2 First Vaccination Visit – Day 1 Visit 37
6.2.3 Day 8 Visit, 7 (+1) days after the study vaccination – Clinic follow-up visit 39
6.2.4 Day 29 Visit, 28 (+2) days after the study vaccination – Clinic follow-up visit 39
6.2.5 Day 36 Visit, 7 (+1) days after the second study vaccination – Clinic follow-up visit 41
6.2.6 Day 57 Visit, 28 (+2) days after the second study vaccination – Clinic follow-up visit 41
6.2.7 Day 169 Telephone Visit, 168 (+14) days after the initial study vaccination – Final Study Visit 42
6.2.8 Unscheduled Visits 43

6.3 Discontinuation of Vaccination or Study Procedures 43
6.4 Withdrawal from Study 43
6.5 Loss to Follow-up 44
6.6 Use of Concomitant Medications During the Study 44
6.7 Emergency Unblinding 45
6.8 Management of Birth Control and Pregnancy During Study 45
6.9 Clinical Assessments 45

7 LABORATORY EVALUATIONS/REQUIREMENTS 46
7.1 Sample Collection, Distribution and Storage 46
7.2 Clinical Laboratory Tests 47
7.3 Assessment of Shed Poliovirus 47
7.4 Future Use of Stored Samples 48

7.5 Biohazard Containment 48

8 SAFETY ASSESSMENT AND REPORTING 48

8.1 Definitions 48

8.1.1 Adverse Event (AE) 48
8.1.2 Adverse reaction / Suspected Adverse Reaction (21CFR Part 312.32) 49
8.1.3 Serious Adverse Event (SAE) 50

8.2 Reporting Period and Parameter 50
8.3 Severity of Adverse Events 50
8.4 Causality of Adverse Event 52
8.5 Follow-up of Adverse Event 52
8.6 General Guidance on Recording Adverse Events 53
8.7 Reporting of SAEs 53
8.8 Protocol Deviations 54

9 SAFETY OVERSIGHT 55

9.1 Routine Reviews by Protocol Safety Review Team (PSRT) 55
9.2 Independent Data Monitoring Committee (IDMC) Reviews 55
9.3 Study Pause Rules 55
9.4 Study Pause Procedure 56

10 DATA HANDLING and RECORDKEEPING 56

10.1 Definitions 56

10.1.1 Source Data 56
10.1.2 Source Documents 57

10.2 Data Capture Methods (Case Report Form Development and Completion) 57
10.3 Data Management 57
10.4 Retention of Study Records 57

11 STATISTICAL CONSIDERATIONS 58

11.1 Overview and General Design 58
11.2 Randomization Procedures 58
11.3 Sample Size 58
11.4 Definitions of Populations to be Analyzed 61

11.4.1 Enrolled Population 61
11.4.2 Safety Population 61
11.4.3 Full Analysis Population 61
11.4.4 Per-Protocol Population 61
11.4.5 Other Populations 62

11.5 Analytical Methodology 62

11.5.1 Descriptive Methodology 62

11.5.2 Analysis Sequence 62
11.5.3 Changes in Analysis Plan 62
11.5.4 Baseline and Demographic Characteristics, and Participant Disposition 62
11.5.5 Safety Analysis 63
11.5.6 Immunogenicity Analysis 64
11.5.7 Viral Shedding Analysis 65
11.5.8 Genetic Stability Analyses 66
11.5.9 Handling of Dropouts and Missing Data 67
12 QUALITY ASSURANCE AND QUALITY CONTROL 67
12.1 General Considerations 67
12.2 Study Monitoring 67
12.3 Independent Auditing 68
12.4 Regulatory Agency Auditing 68
13 ETHICAL CONSIDERATIONS (AND INFORMED CONSENT) 68
13.1 Ethical Standards 68
13.2 Ethical Review 68
13.3 Informed Consent Process 68
13.4 Participant Confidentiality 69
13.5 Reimbursement 69
13.6 Risk and Benefits 70
13.7 Reporting to Local Health Department 71
13.8 Compensation for Research Related Injury 71
14 FINANCING AND INSURANCE 72
15 PUBLICATION POLICY 72
16 REFERENCES 73
APPENDICES 74
APPENDIX 1: SCHEDULE OF STUDY VISITS AND EVALUATIONS 74

LIST OF TABLES

Table 1: Genetic modifications of Sabin-2 in nOPV2-c1 and their purposes 22
Table 2: Methods and criteria on which nOPV1 was evaluated. 24
Table 3: Methods and criteria on which nOPV3 was evaluated. 25
Table 4: Grading scale to grade the severity of solicited AEs 50
Table 5: Grading scale to grade the severity of abnormal clinical safety laboratory test results reported as AEs 51

LIST OF FIGURES

Figure 1: Modifications in nOPV1 and nOPV3 viruses. 23

ABBREVIATIONS AND ACRONYMS

Ab	Antibody
AE	Adverse Event
ALT	Alanine Transaminase
AUC	Area Under the Curve
BSL-2	Biosafety Level 2
CBC	Complete Blood Count
$CCID_{50}$	Cell Culture Infectious Dose 50%
CDC	United States Centers for Disease Control and Prevention
cDNA	Complementary Deoxyribonucleic Acid
CFR	Code of Federal Regulations
CI	Confidence Interval
CONSORT	Consolidated Standards of Reporting Trials
COVID-19	Coronavirus Disease 2019
CRE	Cis-acting Replication Element
CRF	Case Report Form
CRO	Contract Research Organization
CSR	Clinical Study Report
CTA	Clinical Trial Agreement
cVDPV	circulating Vaccine-Derived Poliovirus
DM	Data Management
DRM	Data Review Meeting
eCRF	Electronic Case Report Form
EIA	Enzyme Immunoassay
FAP	Full Analysis Population
FDA	(United States) Food and Drug Administration

FOCP	Female of Childbearing Potential
GCP	Good Clinical Practice
GMFR	Geometric Mean Fold Rise
GMT	Geometric Mean Titer
HBV	Hepatitis B Virus
HCV	Hepatitis C Virus
HIV	Human Immunodeficiency Virus
IB	Investigator's Brochure
ICF	Informed Consent Form
ICH	International Council for Harmonisation
ICMJE	International Committee of Medical Journal Editors
IDMC	Independent Data Monitoring Committee
IEC	Independent Ethics Committee
IgA	Immunoglobulin A
IgG	Immunoglobulin G
IND	Investigational New Drug
IPV	Inactivated Poliovirus Vaccine
IRB	Institutional Review Board
IWRS	Interactive Web Response System
LLN	Lower Limit of Normal
LLOQ	Lower Limit of Quantification
MedDRA	Medical Dictionary for Regulatory Activities
mg	milligram
mL	milliliter
mOPV	monovalent Oral Poliomyelitis Vaccine (Sabin)
NAb	Neutralizing Antibody

NGS	Next Generation Sequencing
NRA	National Regulatory Authority
nOPV	novel Oral Poliomyelitis Vaccine
nOPV1	novel Oral Poliomyelitis Vaccine type 1
nOPV3	novel Oral Poliomyelitis Vaccine type 3
OPV	Oral Poliomyelitis Vaccine
PCR	Polymerase Chain Reaction
PI	Principal Investigator
PID	Participant Identification
PP	Per-protocol
PSRT	Protocol Safety Review Team
PT	Preferred Term
RNA	Ribonucleic Acid
SAE	Serious Adverse Event
SAP	Statistical Analysis Plan
SD	Standard Deviation
SIE	Shedding Index Endpoint
SMP	Study Monitoring Plan
SOC	System Organ Class
SOP	Standard Operating Procedure
SUSAR	Suspected Unexpected Serious Adverse Reaction
ULN	Upper Limit of Normal
ULOQ	Upper Limit of Quantification
US(A)	United States (of America)
UTR	Untranslated Region
VAPP	Vaccine-Associated Paralytic Polio
VDPV	Vaccine-Derived Poliovirus

VS	Vital Signs
WBC	White Blood Cell
WHO	World Health Organization
WPV	Wild Poliovirus

INVESTIGATOR'S AGREEMENT

Protocol Title: A first-in-human, phase 1, randomized, observer-blind, controlled study to assess the safety and immunogenicity of novel live attenuated type 1 and type 3 oral poliomyelitis vaccines in healthy adults

'The signature below constitutes approval of this protocol and the attachments and provides the required assurances that this trial will be conducted according to all stipulations of the protocol, including all statements regarding confidentiality, and according to local legal and regulatory requirements and ICH E6 guidelines.'

_____ _____

Signature of Principal Investigator at **Date**

[redacted]

_____ _____

Signature of Principal Investigator at **Date**

[redacted]

_____ _____

Signature of Principal Investigator at **Date**

[redacted]

_____ _____

Signature of Principal Investigator at **Date**

[redacted]

KEY ROLES AND CONTACT INFORMATION

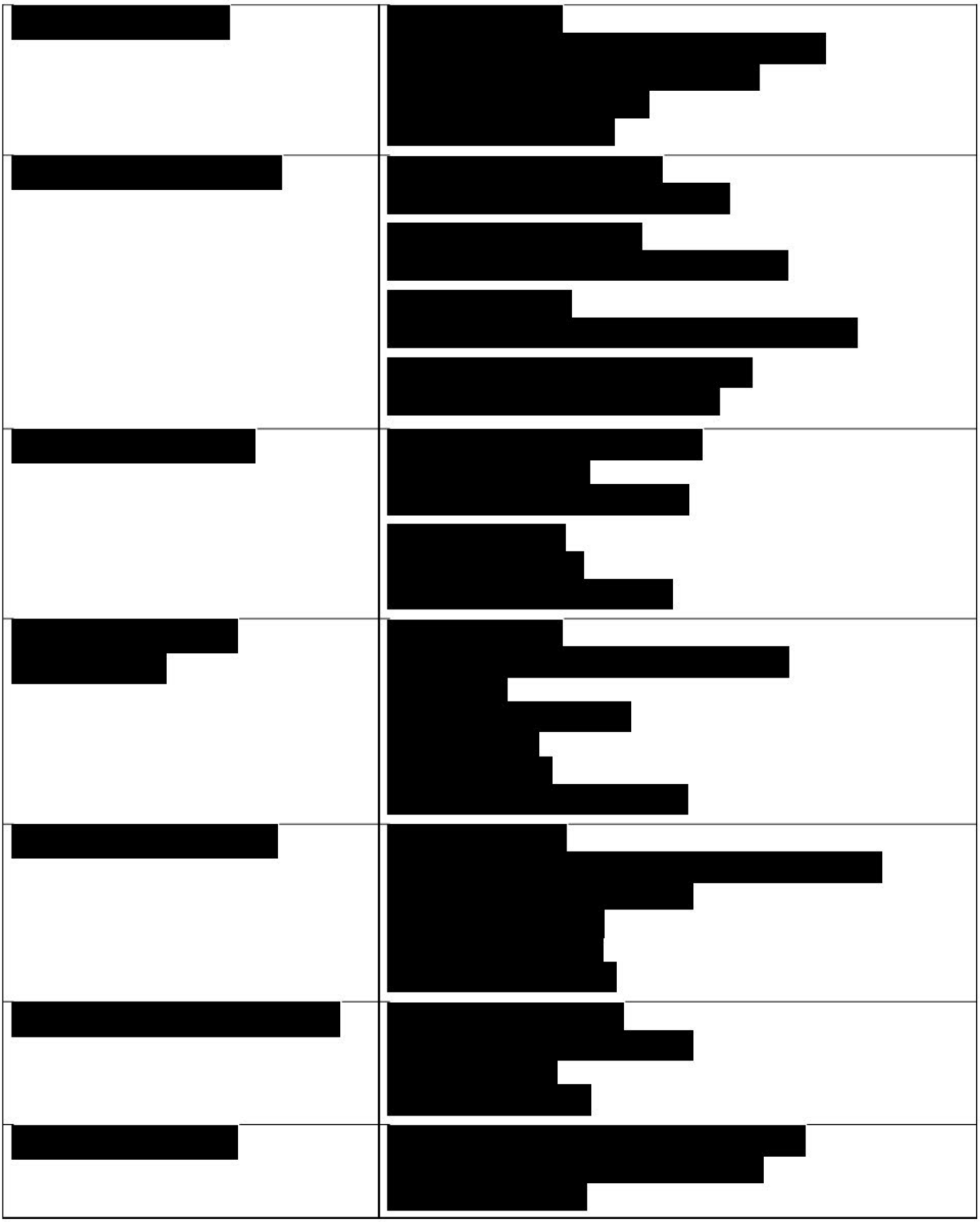

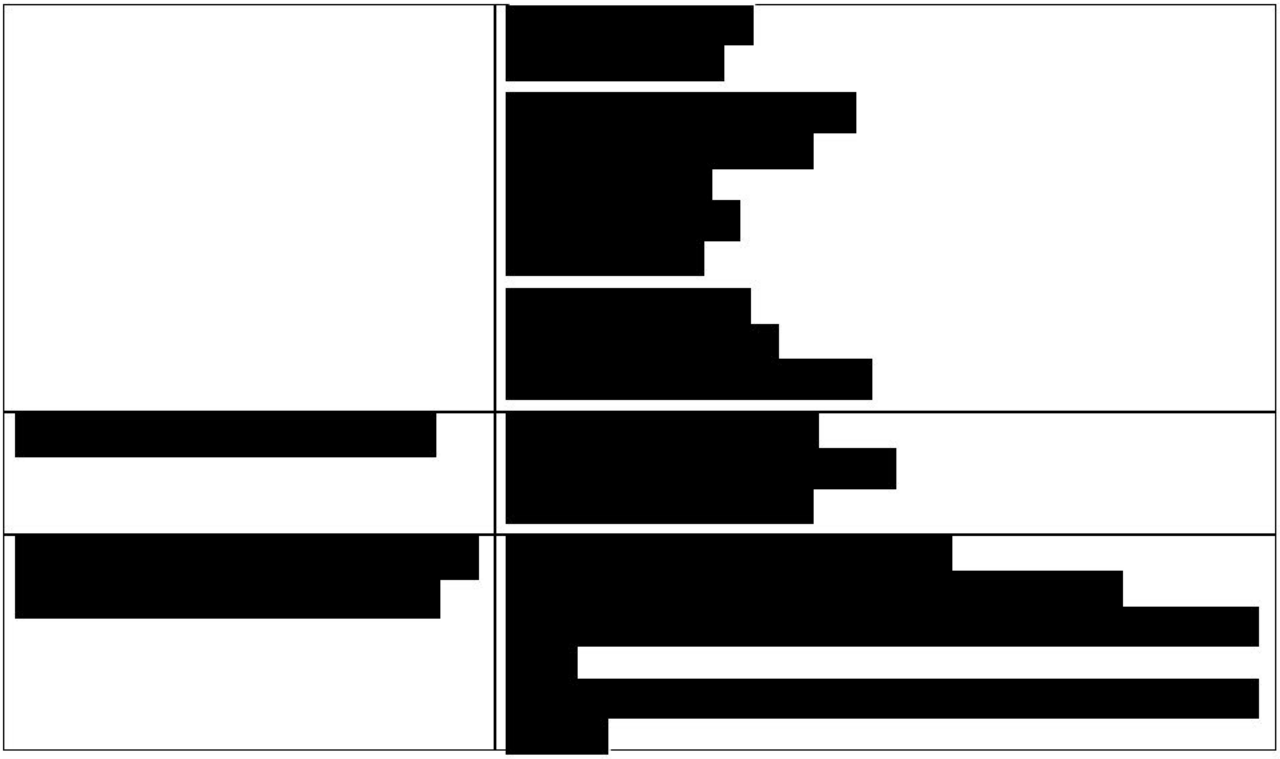

PROTOCOL SUMMARY

**Title**	A first-in-human, phase 1, randomized, observer-blind, controlled study to assess the safety and immunogenicity of novel live attenuated type 1 and type 3 oral poliomyelitis vaccines in healthy adults
**Short Title**	Phase 1 study of novel types 1 and 3 oral poliomyelitis vaccines
**Protocol Number**	CVIA 076
**Trial Registration**	ClinicalTrials.gov: NCT04529538
**FDA Reference Number**	BB-IND 026305
**Trial Phase**	Phase 1
**Investigational Products**	**Novel oral poliomyelitis vaccines (nOPVs):**<br>• Novel OPV type 1 (nOPV1), containing $10^{6.5}$ $CCID_{50}$/dose, manufactured by Bio Farma, Indonesia<br>• Novel OPV type 3 (nOPV3), containing $10^{6.5}$ $CCID_{50}$/dose, manufactured by Bio Farma, Indonesia<br><br>Both nOPVs are live, attenuated polioviruses derived from a modified Sabin type-2 infectious complementary deoxyribonucleic acid (cDNA) clone and propagated in Vero cells. Modifications were introduced in the viral nucleotide sequences in part of the 5'-untranslated region (UTR) to improve the genetic stability of this major attenuating determinant of Sabin type-2, and two modifications were made in the 3D polymerase to further improve stability of the attenuation and reduce recombination. In addition, a key replication element was relocated from the 2C coding region to the 5' UTR to reduce the risk of loss of stabilized 5' UTR through recombination. The cDNA plasmid was then further modified by replacement of the capsid (P1) region of the genome with the capsid from a Sabin-1 (nOPV1) or Sabin-3 (nOPV3) clone, generating chimeric viruses with novel type 2 non-structural regions coupled with Sabin-1 or -3 structural proteins.<br><br>In addition to the attenuated poliovirus, the vaccine formulation includes sucrose, acetic acid or $NaHCO_3$, and Basal Medium Eagle (BME) solution.<br><br>**Active control monovalent oral poliomyelitis vaccines (mOPVs), Sabin:**<br>• Sabin mOPV1 containing not less than $10^{6.0}$ $CCID_{50}$/dose, manufactured by Bio Farma (Indonesia)<br>• Sabin mOPV3 containing not less than $10^{5.8}$ $CCID_{50}$/dose, manufactured by Bio Farma (Indonesia)<br><br>The live types 1 & 3 OPVs contain suspensions of types 1 and 3 attenuated poliomyelitis viruses (Sabin strains) prepared in primary monkey kidney cells. Each dose (2 drops = 0.1 mL) of the two vaccines contains not less than $10^{6.0}$

	infective units of type 1 and $10^{5.8}$ of type 3, respectively. Sucrose is used as a stabilizer. The vaccines may contain trace amounts of not more than 2 mcg erythromycin and not more than 10 mcg kanamycin.
**Study Hypotheses**	Safety<br>• The live attenuated, novel oral poliomyelitis vaccine type 1 (nOPV1) is safe and well-tolerated in healthy adults<br>• The live attenuated, novel oral poliomyelitis vaccine type 3 (nOPV3) is safe and well-tolerated in healthy adults<br>Immunogenicity<br>• nOPV1 and nOPV3 elicit demonstrable immune responses in healthy adults
**Study Objectives**	**Primary Objective:**<br>**Safety**<br>• To evaluate the safety and tolerability of nOPV1 and nOPV3 in healthy adults<br>**Secondary Objectives:**<br>**Immunogenicity**<br>• To assess the humoral immune responses (neutralizing antibody titers) elicited by nOPV1 and nOPV3, and compare to that of mOPV1 and mOPV3, respectively, in healthy adults<br>**Fecal Shedding of Study Vaccine Viruses**<br>• To assess the duration of fecal shedding (as determined by polymerase chain reaction [PCR]) of nOPV1 and nOPV3 after the initial dose and compare to that of the homotypic mOPVs<br>• To assess the rate of fecal shedding (as determined by PCR) of nOPV1 and nOPV3 and compare to that of the homotypic mOPVs in participants with an exclusive inactivated poliovirus vaccine (IPV) prior vaccination history<br>• To assess the extent of shedding (as determined by a shedding index endpoint [SIE] and the area under the curve [AUC]) of nOPV1 and nOPV3 and compare to that of the homotypic mOPVs in participants with an exclusive IPV prior vaccination history<br>**Exploratory Objectives:**<br>• To assess the duration of fecal shedding (as determined by PCR) of nOPV1 and nOPV3 after the second dose and compare to that of the homotypic mOPVs<br>• To assess the rate of fecal shedding (as determined by PCR) of nOPV1 and nOPV3 and compare to that of the homotypic mOPVs in participants with an OPV-containing prior vaccination history

	• To assess the extent of shedding (as determined by an SIE and the AUC) of nOPV1 and nOPV3 and compare to that of the homotypic mOPVs in participants with an OPV-containing prior vaccination history<br>• To assess the potential for neurovirulence of nOPV shed virus as measured by a transgenic mouse neurovirulence assay, and compare to that of mOPV1 and mOPV3<br>• To assess genetic stability of nOPV shed virus as determined by next-generation sequencing (NGS)<br>• To assess the humoral immune responses elicited by nOPV1 and nOPV3 against non-vaccine poliovirus types
**Study Endpoints**	**Primary Endpoints:**<br>**Safety:**<br>• Frequency of serious adverse events (SAEs) from Day 1 study vaccination through the end of the study<br>• Frequency of solicited adverse events (AEs) for 7 days (day of study vaccination and 6 following days) after each dose of study vaccine<br>• Frequency of unsolicited AEs for 28 days (day of study vaccination and 27 following days) after each dose of study vaccine [including clinically significant aberrant safety monitoring laboratory values on Day 8 reported as AEs]<br>**Secondary Endpoints:**<br>**Immunogenicity:**<br>• Median type-specific anti-polio serum neutralizing antibody titers at baseline and post-vaccination<br>• Type-specific anti-polio serum neutralizing antibody Geometric Mean Titer (GMT) at baseline and post-vaccination<br>• Post-vaccination GMT ratios of type-specific anti-polio serum neutralizing antibody, adjusted for baseline immunity<br>• Post-vaccination frequency of any fold-rise (titer increased from baseline), a minimum 2-fold rise, and a minimum 4-fold rise (seroconversion), in type-specific anti-polio serum neutralizing antibody responses<br>**Fecal Shedding of Study Vaccine Viruses**<br>• Time to cessation of fecal shedding (days) of the vaccine virus, following initial dose<br>• Proportion of participants shedding type-specific vaccine virus at each post-vaccination stool collection, as assessed by PCR in participants with an exclusive IPV prior vaccination history<br>• Amount of vaccine virus in each stool sample (log_{10} $CCID_{50}$ per gram) positive for virus (PCR) in participants with an exclusive IPV prior vaccination history

	• Shedding Index of vaccine virus shedding in stool, defined as the mean of log_{10} $CCID_{50}$ per gram of stool at 7, 14, 21 and 28 days following each dose in participants with an exclusive IPV prior vaccination history<br>• AUC of vaccine virus shed in stool in participants with an exclusive IPV prior vaccination history<br>**Exploratory Endpoints**<br>• Time to cessation of fecal shedding (days) of the vaccine virus, following second dose<br>• Proportion of participants shedding type-specific vaccine virus at each post-vaccination stool collection, as assessed by PCR in participants with an OPV-containing prior vaccination history<br>• Amount of vaccine virus in each stool sample (log_{10} $CCID_{50}$ per gram) positive for virus (PCR) in participants with an OPV-containing prior vaccination history<br>• Shedding Index of vaccine virus shedding in stool, defined as the mean of log_{10} $CCID_{50}$ per gram of stool at 7, 14, 21 and 28 days following each dose in participants with an OPV-containing prior vaccination history<br>• AUC of vaccine virus shed in stool in participants with an OPV-containing prior vaccination history<br>• Neurovirulence of shed vaccine virus from select stool samples as measured by a transgenic mouse neurovirulence test<br>• Deep sequencing of shed vaccine virus from select stool samples using NGS<br>• Serum neutralizing antibody titers against non-vaccine poliovirus types at baseline and post-vaccination

Study Design

This multicenter trial is the first-in-human assessment of these novel oral poliomyelitis vaccines. It will be an 8-arm, randomized, observer-blind, controlled trial, with Sabin monovalent vaccines serving as the control for each type (see study schema, below). 150 to 230 healthy, adult participants will be recruited. 30 to 40 of 60 to 80 participants with an exclusive IPV prior vaccination history (cohort 1) will be randomized in a 1:1 ratio to study groups 1 and 2 and allocated to receive nOPV1 or mOPV1, and once these study arms are completely accrued, the other 30 to 40 exclusively-IPV vaccinated participants (cohort 3) will be randomized in a 1:1 ratio to study groups 5 and 6 and allocated to receive nOPV3 or mOPV3.

In parallel, 45 to 75 of 90 to 150 participants with an OPV-containing prior vaccination history (cohort 2) will be randomized in a 2:1 ratio to study groups 3 and 4 and allocated to receive two doses of nOPV1 or mOPV1, respectively, and once these study arms are completely accrued, the other 45 to 75 participants with an OPV-containing prior vaccination history (cohort 4) will be randomized in a 2:1 ratio to study groups 7 and 8 and allocated to receive two doses of nOPV3 or mOPV3, respectively.

Study Schema

Cohort	Group	Prior Vaccination	Number of Participants (Min-Max)	Dosage $CCID_{50}$		Day 1	Day 29
**1**	1	IPV	15-20	$10^{6.5}$		nOPV1	-
	2		15-20	$\geq 10^{6}$		mOPV1	-
**Subtotal**			30-40				
**2**	3	OPV	30-50	$10^{6.5}$		nOPV1	nOPV1
	4		15-25	$\geq 10^{6}$		mOPV1	mOPV1
**Subtotal**			45-75				
**3**	5	IPV	15-20	$10^{6.5}$		nOPV3	-
	6		15-20	$\geq 10^{5\ 8}$		mOPV3	-
**Subtotal**			30-40				
**4**	7	OPV	30-50	$10^{6.5}$		nOPV3	nOPV3
	8		15-25	$\geq 10^{5\ 8}$		mOPV3	mOPV3
**Subtotal**			45-75				
**Total**			150-230				

Participants assigned to two doses of vaccine will receive the second dose 28 days following the first dose. Initial vaccination for Cohorts 1 and 2 participants will be limited to no more than 5 participants per day and a total of 10 participants during the first week of vaccination. Participants will be followed until 24 weeks after their Day 1 study vaccination.

Study population

Healthy adults, 18-45 years of age (inclusive)

**Participating sites**	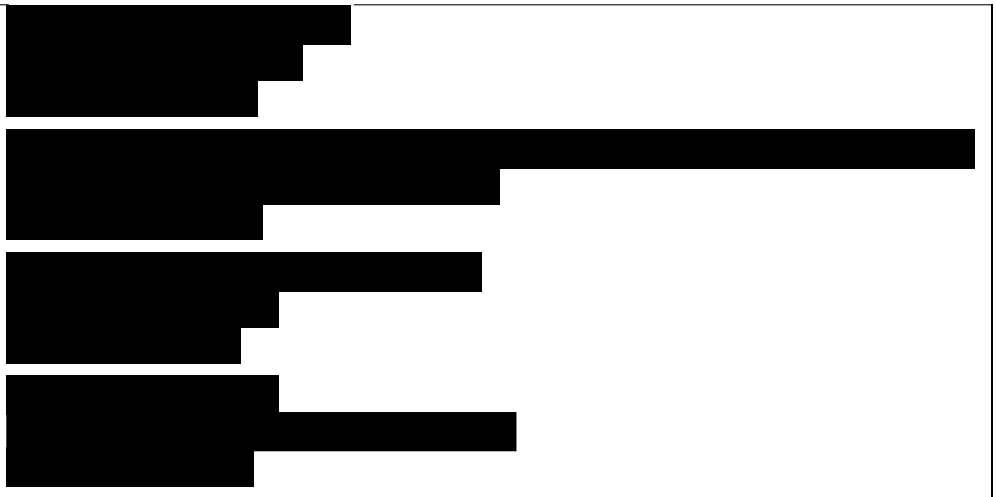
**Study Duration**	Study participation will last about 7 months, allowing for 24 weeks of follow-up after the receipt of the initial study vaccination.

1 BACKGROUND AND RATIONALE

Global efforts to immunize children with Sabin strain oral poliomyelitis vaccines (OPVs) have reduced wild poliovirus cases by 99.9% since 1988 [1]. These vaccines have been demonstrated to be safe and interrupt person-to-person spread of polioviruses. However, on extremely rare occasions, use of OPV can result in cases of polio due to vaccine-associated paralytic polio (VAPP) and circulating vaccine-derived poliovirus (cVDPV). Central to both VAPP and disease induced by cVDPVs is reversion of the vaccine strain to a more neurovirulent phenotype, which occurs during intestinal replication in vaccine recipients. These reverted viruses can either cause disease in the vaccine recipient or be transmitted to contacts or community members. In addition, the ability of cVDPVs to survive in the environment and be transmitted to others without being detected through acute flaccid paralysis surveillance, given most infections are asymptomatic, is a major risk to the entire polio eradication effort. The risk of VAPP and cVDPV, in particular, resulted in the decision to globally discontinue routine use of Sabin type 2 OPV (OPV2) in April 2016 and the planned withdrawal of Sabin types 1 and 3 OPV (OPV1 and OPV3, respectively) in the coming decade. The intent of the novel OPV program is to develop more genetically stable versions of type 1 and type 3 oral poliomyelitis vaccines to reduce the risk of VAPP and cVDPV from vaccine administered to combat outbreaks by types 1 and 3 polioviruses.

Genetically modified candidate viruses for both polioviruses type 1 and type 3 have been developed that retain similar immunogenicity and antigenicity to the parental Sabin OPV strains in animal models, while demonstrating significantly less potential to revert during cell culture passaging. These viruses also appear to have a reduced neurovirulence in a transgenic mouse disease model. The proposed indication and usage is for prevention of type 1 or type 3 poliovirus disease and transmission during outbreaks of either wild-type poliovirus or cVDPV.

The proposed study is the first-in-human evaluation of novel OPVs types 1 and 3 (nOPV1 and nOPV3, respectively) in healthy males and females, from 18 to 45 years of age (inclusive) at the time of enrollment. Should results in adults indicate the nOPVs are safe and have demonstrable immunogenicity, they will progress to phase 2 testing in target pediatric populations (young children and then infants).

A similar novel, genetically stable OPV type 2 (nOPV2) strain has advanced into phase 2 clinical development (adults in Belgium and young children and infants in Panama) with encouraging study results. Since nOPV1 and nOPV3 are chimeric viruses with novel type 2 non-structural regions coupled with Sabin-1 or -3 structural proteins, the clinical experience to date with nOPV2 is relevant.

1.1 Burden of Disease

Polio is an infectious disease caused by polioviruses that are transmitted by person-to-person spread, mainly through the fecal-oral route or, less frequently, by a common vehicle, such as contaminated water or food. Three poliovirus types 1, 2, and 3 exist and infection with each confers type-specific life-long immunity to disease but little or no immunity to disease caused by heterologous types. Over 90% of poliovirus infections are asymptomatic. Symptomatic disease is more often an acute self-limited non-neurologic febrile illness with non-specific symptoms, such as headache, sore throat, fatigue, and gastrointestinal symptoms. Less often, disease may manifest with myalgias, meningitis, and other neurologic findings, with overt paralysis occurring in less than 1% of all infections. A major complication of paralytic poliomyelitis is respiratory failure. There are no licensed antiviral drugs available to treat poliomyelitis and treatment is supportive.

Since the introduction of IPVs and OPVs in the 1950s and the launching of the Global Polio Eradication Initiative campaign in 1988, transmission of wild poliovirus (WPV) has been interrupted in most

populations. Globally, type 2 WPV was declared eradicated in 2015, type 3 WPV declared eradicated in 2019, with current transmission of WPV limited to type 1 and confined to a few regions within only three endemic countries, Nigeria, Pakistan, and Afghanistan. For the year 2019, there were 176 type 1 WPV cases reported, all from two of the three endemic countries, Pakistan, and Afghanistan, while there were 365 cVDPV cases (mostly type 2) reported, 324 of these cases from non-endemic countries [http://polioeradication.org]. Until all WPV transmission is interrupted globally and eradicated, all countries remain at risk of importation of polio from endemic countries, especially vulnerable countries with weak public health and immunization services and travel or trade links to endemic countries. The WHO estimates that failure to eradicate polio from these last remaining regions could result in as many as 200,000 new cases every year, within 10 years, all over the world.

As long as OPV remains the vaccine used globally to achieve eradication, there remains the risk of vaccine-derived poliovirus (VDPV) emergence and spread, which is why eradication of WPV is to be followed with cessation of OPV use in routine immunization, as was done for OPV2 in 2016, with the switch from trivalent OPV to bivalent OPV 1,3. In 2019, three years following the end of immunization with nOPV2, there has been a notable increase in the number of cVDPV type 2 cases compared to prior years, reflecting the increased susceptibility to type 2 infection in at risk regions. From 2000 to 2019, 1085 cases of paralytic cVDPV have been reported, 932 (86%) of which were type 2, notwithstanding that it is estimated that during the same period, over 10 million cases of paralytic polio were averted [2]. With the eradication of type 3 and expected eventual eradication of type 1 WPV, it is anticipated that the concern over cVDPV shall remain. It is hoped that if the nOPVs prove to be safe and effective in the planned and ongoing studies, future cVDPV outbreaks and associated paralytic cases in communities with low immunity could be addressed with these novel vaccines.

1.2 Pathogen

Polioviruses are small, non-enveloped viruses with a single positive strand ribonucleic acid (RNA) genome of approximately 7,400 nucleotides. The genome contains a relatively long non-coding region in the 5' end, referred to as 5' untranslated region (5' UTR), which is highly structured and contains the internal ribosome entry site that is required for translation. The genome is translated as a single polyprotein, which is cleaved during translation by genome-encoded proteases into three regions referred to as P1, P2, and P3. The P1 region is further processed into viral structural (capsid) proteins, while P2 and P3 encode the non-structural proteins that are involved in protein processing and viral replication. There are three serotypes, defined by their capsid (P1) regions, with limited cross-neutralization across the three.

1.3 Description of Study Vaccine

The Sabin OPV strains have been studied intensively for decades and, because of the knowledge and experience gained, several approaches to the design of improved OPV strains have been investigated. In 2011, a consortium of researchers was formed to develop improved strains, with the belief that a collaborative effort using a combination of strategies would have the greatest chance of success. Novel OPV (nOPV) strains were designed, produced, and tested in a variety of non-clinical studies. Two type 2 strains have advanced into clinical development with encouraging phase 1 and 2 study results.

The approach to the design of nOPV strains addressed five critical inter-related properties: cell-culture growth, attenuation, genetic stability (encompassing reversion through mutation and recombination), antigenicity, and immunogenicity. The designs involved innovative strategies and their success was assessed by rigorous testing carried out in parallel for all strains to facilitate the choice of suitable candidates.

With the overall goal of seeking novel OPV1 and OPV3 (nOPV1, nOPV3) vaccines which have less chance of reversion to a neurovirulent phenotype and comparable immunogenicity to the current Sabin vaccines, one candidate for each type has been selected for clinical development thus far.

The candidate OPV1 and OPV3 strains include 4 distinct modifications of the Sabin-2 genome, including changes to the RNA sequence in the 5' UTR, the non-structural protein 2C, and the polymerase 3D (Table 1 and Figure 1) [3]. Of these modifications, only the changes to polymerase 3D result in a change in the amino acid sequence. The modifications aim to stabilize the genetic sequence against reversion in the 5' UTR and to reduce recombination. The vaccine virus initially containing these modifications—a novel oral poliomyelitis vaccine type 2 candidate 1 (nOPV2-c1) has been advanced into clinical development. The cDNA plasmid used to derive nOPV2-c1 was then further modified by replacement of the capsid (P1) region of the genome with the capsid from a Sabin-1 (nOPV1) or Sabin-3 (nOPV3) clone, generating chimeric viruses with novel type 2 non-structural regions coupled with Sabin-1 or -3 structural proteins. The nOPV study vaccines are named nOPV1 candidate 1 and nOPV3 candidate 1 (abbreviated nOPV1-c1 and nOPV3-c1, respectively). The use of the designation 'candidate 1' is due to the potential subsequent development of additional candidate type-specific vaccines. For purposes of simplification, in this protocol these study vaccines will be referred to as nOPV1 and nOPV3, respectively.

Table 1: Genetic modifications of Sabin-2 in nOPV2-c1 and their purposes.

Modification	Purpose
S15 dom V	-Improved stability of attenuated phenotype. Specifically, improve genetic stability of the domain V attenuating mutation to avoid reversion by single nucleotide changes.<br>-Lack of reversion may reduce shedding and transmission risk.
Cre relocation	-Reduce frequency of recombination events. Specifically, a single recombination event replacing dom V will also remove cre, making virus non-viable and non-infectious.
Polymerase (higher fidelity)	-Improved stability of attenuated phenotype. Specifically, improve fidelity of replication leading to less genetic drift and reversion.<br>-Additional attenuation.
Polymerase (rec)	-Reduce frequency of recombination events, thereby reducing ability of population to improve replication fitness.<br>-Additional attenuation.

Figure 1: Modifications in nOPV1 and nOPV3 viruses.

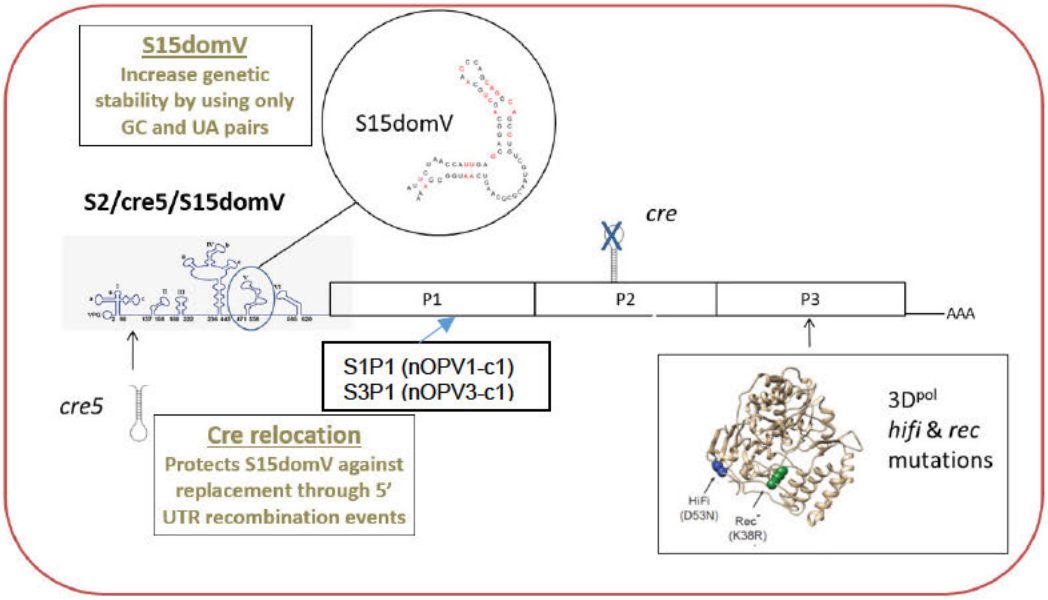

Sabin 2 genome is depicted showing the 5' UTR in grey shading, polyproteins (P1-3), 3' UTR and polyA; locations of modifications within the genome are shown. Nucleotide differences between Sabin 2 and S15 domain V are shown in red.

1.4 Summary of Nonclinical Studies of Study Vaccine

Key criteria for evaluation and selection of vaccine candidates for clinical development were based on growth in cell culture, antigenicity, immunogenicity, attenuation, and genetic stability (i.e., resistance to reversion to a more neurovirulent phenotype).

This section describes the non-clinical evaluation of the nOPV virus seeds for each of the two nOPVs. The specific methods and criteria are summarized in Table 2 and Table 3. Non-clinical (including reproductive) toxicology studies have not been conducted on nOPV1 or nOPV3. The rationale for the lack of these toxicology studies is based on the following considerations:

- Lack of a relevant animal model for oral poliovirus infection and toxicology at relevant human dose
- Ethical implications of conducting an animal study that is unlikely to provide useful data or insight
- Strength of the neurovirulence data available on research and GMP seed virus
- Similarity of nOPV1 and nOPV3 to Sabin OPVs, which have been used for decades and have a well-established safety profile
- A separate non-clinical toxicology study of nOPV1 or nOPV3 is of limited value to establish the safety of the vaccine product prior to initiating clinical vaccine development in a fully vaccinated population

For further details refer to the Investigator's Brochure (IB).

1.4.1 Growth and yield in Vero cells

A critical criterion is the ability of the nOPVs to grow effectively under conditions similar to those to be used for manufacturing of clinical and future commercial supplies, in order to ensure that any clinical vaccine candidate could ultimately be manufactured in adequate supply and at an acceptable cost.

Both nOPVs have demonstrated similar growth properties to type-matched Sabin strains in Vero cell culture at 33°C, which suggests that routine manufacture of these vaccines at an acceptable cost will be achievable. In addition, temperature sensitivity mapping (RCT) of both nOPVs showed them to be similar to the Sabin strains, which represents another confirmation of their attenuated nature.

Table 2: Methods and criteria on which nOPV1 was evaluated.

Attribute	Criteria	nOPV1-c1	Sabin OPV1 control
Attenuation, Tg66 Mice	PD_{50} ≥ Sabin-1 (PD_{50} is estimated concentration of virus which paralyzes 50% of the mice)	PD_{50} > 8.7 log (0/8 paralyzed at max dose)	PD_{50} = 3.1 log
Attenuation after passaging in Vero cells, Tg66 Mice	PD_{50} > Sabin-1	PD_{50} > 8.4 log (2/8 paralyzed at max dose)	PD_{50} = 2.2 log
Deep Sequencing after passaging in Vero cells	No changes in modified regions, including 5' UTR dom V	Passed	Clear evidence of reversion at nucleotides 480 / 525
Antigenicity	Similar reactivity against 4 neutralizing monoclonal antibodies targeting different antigenic sites	Yes	
Immunogenicity in juvenile TgPVR mice	Dose-dependent seroconversion comparable to Sabin-1	Yes	
Yield 33°C (MOI 0.1, Vero)	Titer no greater than 1 log_{10} below that of Sabin-1	Yes	

Table 3: Methods and criteria on which nOPV3 was evaluated.

Attribute	Criteria	nOPV3-c1	Sabin OPV3 control
Attenuation, Tg66 Mice	$PD_{50} \geq$ Sabin-3 (PD_{50} is estimated concentration of virus which paralyzes 50% of the mice)	$PD_{50} > 8.8$ log (0/8 paralyzed at max dose)	$PD_{50} = 4.2$ log
Attenuation after passaging in Vero cells, Tg66 Mice	$PD_{50} >$ Sabin-3	$PD_{50} > 8.4$ log (0/8 paralyzed at max dose)	$PD_{50} = 1.7$ log
Deep Sequencing after passaging in Vero cells	No changes in modified regions, including 5' UTR dom V	Passed	Clear evidence of reversion at nucleotides 472
Antigenicity	Similar reactivity against 4 neutralizing monoclonal antibodies targeting different antigenic sites	Yes	
Immunogenicity in juvenile TgPVR mice	Dose-dependent seroconversion comparable to Sabin-3	Yes	
Yield 33°C (MOI 0.1, Vero)	Titer no greater than 1 $\log_{10}$ below that of Sabin-3	Yes	

1.4.2 Antigenicity

Antigenic structure was analyzed using an assay similar to that used to measure the D-antigen content of poliovirus vaccines [4] which is a non-competitive sandwich ELISA. Four monoclonal antibodies, specific for native conformations of antigenic sites 1, 2, 3 and 4 of each type were used as primary antibodies [5,6].

Both nOPVs reacted with monoclonal antibodies against the four antigenic sites tested in ELISA, and the dose-response curves were parallel to those of the homotypic Sabin strain, suggesting a high degree of antigenic similarity between both the nOPVs and Sabins, as anticipated. This was consistent with the fact that there were no amino acid differences in the capsid proteins between the strains and matched Sabin controls.

1.4.3 Immunogenicity

Because Sabin vaccine strains of polioviruses replicate poorly (or not at all) in animal models, the relative immunogenicity of new vaccine strains dosed by the oral route can only be definitively evaluated in human clinical studies. Nonetheless, a mouse model including both interferon-receptor knock-out and transgenic expression of the human poliovirus receptor (IFNAR-/- TgPVR21) has recently been developed [7], which provides useful data. In this model, unlike other immunogenicity models [8], seroconversion is dependent on replication, which helps to provide some reassurance that these new strains will be immunogenic in humans.

In these experiments, 10 juvenile IFNAR-/- TgPVR21 mice (per group) were inoculated intraperitoneally with a range of doses of Sabin strains or the research seed of the candidates. After 21 days, the mice were

sacrificed, bled, and sera frozen. Neutralizing antibody titers in sera were determined by standard methods using 100 $TCID_{50}$ units of corresponding Sabin strain as the challenge virus.

In this model, mice receiving placebo consistently have titers of less than 4. For nOPV1 and the Sabin-1 control, consistent positive responses were seen across the dosage range, with similar titers for the two at the higher dosages. For type 3, nOPV3 and the Sabin-3 control responses were similar across the full dosage range.

As noted above, in this model seroconversion is dependent on replication; inactivated preparations are not immunogenic. Hence the immunogenicity, and by implication, infectivity of these two nOPVs was confirmed to be similar to the Sabin controls. It is not suggested that this model reflects the environment of the human gut; however, the assay provides evidence that the candidates are phenotypically similar to Sabin-2 in a model that depends on replication and antigen presentation *in vivo*. These data, when coupled with the encouraging immunogenicity of the parental nOPV2 strain in humans, provides confidence that these nOPV strains are likely to be immunogenic in humans.

1.4.4 Attenuation phenotypes

The non-clinical data suggest that these nOPVs have an improved risk profile as compared to the Sabin OPV strains. Regarding risk reduction, the following observations on the nOPV virus seeds supporting this position were made:

- Both nOPV1 and nOPV3 passed the WHO monkey neurovirulence test, with lower lesion scores than the reference controls and no paralysis of any monkeys.
- In a transgenic mouse neurovirulence model, no dose of either nOPV could be established that resulted in paralysis of any mice.
- The temperature-sensitive phenotype, a marker for attenuation, was retained for both nOPVs.
- Upon passaging in cell culture under conditions known to lead to reversion, both deep sequencing and mouse neurovirulence testing confirmed that the nOPVs did not revert to any meaningful extent, while the Sabin strains showed clear reversion by both methods.

Incremental benefits of the polymerase modifications and cre relocation on genetic stability also were demonstrated using targeted experiments.

In total, these results suggest that the potential risks associated with OPV use (VAPP and cVDPV circulation) should be lower for nOPV1 and nOPV3 than for the Sabin comparators. This conclusion is further supported by results from phase 1 and 2 clinical studies of the parental nOPV2 strain (from which these nOPVs are derived) which demonstrated an absence of reversion of domain V and limited increases in virulence for shed virus [9].

1.5 Summary of Clinical Studies of Study Vaccine

Although nOPV1 and nOPV3 have not previously been tested in humans, two candidates of novel OPV2 (nOPV2-c1 and nOPV-c2) have been tested in three clinical trials: phase 1 and 2 trials in adults in Belgium and a phase 2 trial in young children and infants in Panama. As described earlier, the construct of one of these vaccine candidates, nOPV2-c1, is very similar to nOPV1 and nOPV3, with the only difference being the type-specific capsid. For both phase 2 studies, safety, immunogenicity, shedding and genetic stability data were compared to data from studies performed 2 to 3 years prior with mOPV2 and designed to provide data for the comparison (historical control studies). Data for genetic stability in the pediatric study have not yet been finalized, but interim data are available from the young children in that study. A

summary of the three study descriptions followed by the data for nOPV2-c1, which is also the candidate moving forward into phase 3 testing, is presented below, and more detailed results can be found in the IB.

Phase 1, First-in-Human Study in Adults in Belgium

A phase 1, first-in-human study was performed under containment at the University of Antwerp [9]. Two cohorts of 15 adult participants each were enrolled sequentially, with all participants in each cohort receiving the same vaccine candidate (at a 10^6 $CCID_{50}$ dosage) to avoid cross-contamination between the two candidates (nOPV2-c1 and nOPV2-c2). All participants had previously received at least 3 doses of IPV, and none had a history of receiving OPV (per eligibility criteria). The assignment of product to the cohorts was performed randomly, and neither study staff nor participants were aware of which product was administered to which cohort. Containment of each cohort lasted until the last participants in each had been in containment for 28 days following administration of study product.

Phase 2 Study in Adults in Belgium

The study was performed at two sites in Belgium (Antwerp and Ghent). The study population of healthy adults included 200 participants who had previously received OPV (100 receiving nOPV2-c1 and 100 receiving nOPV2-c2, half of whom received a single dose (10^6 $CCID_{50}$) and the other half two doses) and another 50 who had previously received only IPV (17 receiving nOPV-c1, 16 receiving nOPV2-c2 and 17 receiving placebo). Participants were sequentially enrolled, with completion of enrollment of those to receive nOPV2-c2 (IPV and OPV groups simultaneously) before initiation of enrollment of those to receive nOPV2-c1 (IPV and OPV groups simultaneously). IPV-only vaccinated participants were randomized 2:1 to receive one of the two vaccine candidates or placebo. Data from participants who had previously received OPV were compared to those of a previous study, also in Belgium and similarly designed, in which previously OPV-vaccinated healthy adults were vaccinated with mOPV2.

Phase 2 Study in Young Children and Infants in Panama

This was a single-center, multi-site, age de-escalation, partly-randomized study performed in Panama. Two different cohorts of participants were recruited: healthy young children enrolled and vaccinated at 1 to 5 years of age and healthy infants enrolled at 6 weeks of age and vaccinated at 18-22 weeks of age. Infants were recruited at 6 weeks of age to assure vaccination with 3 doses of bOPV and 1 dose of IPV prior to vaccination with nOPV2 at 18-22 weeks of age.

The study was performed in two consecutive stages, Stage I and Stage II. In Stage I, young children and infants were vaccinated with nOPV2-c2 manufactured in 2016. In Stage II, young children and infants were vaccinated with nOPV2-c1 and nOPV2-c2 manufactured in 2018. The difference between nOPV2-c2 manufactured in 2016 and 2018 was the working virus seed used for each. Children received 10^6 $CCID_{50}$/dose (high dose), and infants received either 10^5 $CCID_{50}$/dose (low dose) or 10^6 $CCID_{50}$/dose (high dose). All 1-5 year old children were to receive two doses of vaccine, and a subset of 50 infants from each of the six groups were to receive two doses (the balance to receive a single dose). In Stage II, 49 1-5 year old children were vaccinated with the high dose, 138 infants were vaccinated with the low dose, and 150 infants were vaccinated with the high dose of nOPV2-c1.

1.5.1 Safety

The candidate vaccine, nOPV2-c1, was generally well-tolerated in all three studies. Safety data were reassuring, with overall safety profiles similar to those of applicable control groups for the phase 2 studies

(adults in Belgium, and 1-5 year old children and 12-22 week old infants in Panama). No SAE was assessed as causally related to vaccine. Most solicited and unsolicited adverse events (AEs) were mild or moderate, and they were infrequently severe. No unsolicited severe AEs were assessed as related to vaccination in young children and infants, and no AEs resulted in termination from subsequent dosing (in those scheduled to receive two doses) or in withdrawal from the study.

1.5.2 Immunogenicity

In all three studies, nOPV2-c1 was immunogenic, and the immune responses were non-inferior to those following vaccination with mOPV2 in the historical control studies.

1.5.3 Viral Shedding

Results of stool testing for virus shedding generally indicate that nOPV2-c1 is not shed in a substantially greater rate or quantity as compared to mOPV2, and the rate of viral shedding is lower at four weeks following vaccination in a key demographic subgroup (bOPV/IPV-vaccinated infants).

1.5.4 Genetic Stability

Genetic stability assessment of shed virus from the adult participants in the phase 1 and 2 studies in Belgium were consistent with improved genetic and phenotypic stability of the nOPV2 candidates. Interim results from 1-5 year old children in the phase 2 study in Panama are consistent with data from adults and show improved genetic and phenotypic stability of nOPV2-c1; data from infants are pending.

1.6 Overall Development Strategy

The objectives of the nOPV1,3 program are (1) to achieve licensure and World Health Organization (WHO) prequalification of novel OPV1 and OPV3 monovalent vaccines (nOPV1 and nOPV3) with an indication for active immunization against disease caused by poliomyelitis virus type 1 or 3, respectively; (2) to provide sufficient evidence to support a policy decision to stockpile these monovalent vaccines in lieu of the current Sabin vaccines, and (3) to potentially make nOPV1 and/or nOPV3 available pre-licensure during outbreaks, provided the early data demonstrate appropriate safety and immunogenicity. If use of bivalent OPV (bOPV, which includes the Sabin type 1 and type 3 strains) continues for longer than anticipated, nOPV1 and nOPV3 could also be considered for routine use.

2 HYPOTHESIS, OBJECTIVES AND ENDPOINTS

2.1 Study Hypotheses

Safety

- The live attenuated, novel oral poliomyelitis vaccine type 1 (nOPV1) is safe and well-tolerated in healthy adults
- The live attenuated, novel oral poliomyelitis vaccine type 3 (nOPV3) is safe and well-tolerated in healthy adults

Immunogenicity

- nOPV1 and nOPV3 elicit demonstrable immune responses in healthy adults

2.2 Study Objectives

2.2.1 Primary Objective:

Safety

To evaluate the safety and tolerability of nOPV1 and nOPV3 in healthy adults

2.2.2 Secondary Objectives:

Immunogenicity

To assess the humoral immune responses (neutralizing antibody titers) elicited by nOPV1 and nOPV3, and compare to that of mOPV1 and mOPV3, respectively, in healthy adults

Fecal Shedding of Study Vaccine Viruses

- To assess the duration of fecal shedding (as determined by PCR) of nOPV1 and nOPV3 after the initial dose and compare to that of the homotypic mOPVs
- To assess the rate of fecal shedding (as determined by PCR) of nOPV1 and nOPV3 and compare to that of the homotypic mOPVs in participants with an exclusive IPV prior vaccination history
- To assess the extent of shedding (as determined by an SIE and the AUC) of nOPV1 and nOPV3 and compare to that of the homotypic mOPVs in participants with an exclusive IPV prior vaccination history

2.2.3 Exploratory Objectives:

- To assess the duration of fecal shedding (as determined by PCR) of nOPV1 and nOPV3 after the second dose and compare to that of the homotypic mOPVs
- To assess the rate of fecal shedding (as determined by PCR) of nOPV1 and nOPV3 and compare to that of the homotypic mOPVs in participants with an OPV-containing prior vaccination history
- To assess the extent of shedding (as determined by an SIE and the AUC) of nOPV1 and nOPV3 and compare to that of the homotypic mOPVs in participants with an OPV-containing prior vaccination history
- To assess the potential for neurovirulence of nOPV shed virus as measured by a transgenic mouse neurovirulence assay, and compare to that of mOPV1 and mOPV3
- To assess genetic stability of nOPV shed vaccine virus as determined by NGS
- To assess the humoral immune responses elicited by nOPV1 and nOPV3 against non-vaccine poliovirus types

2.3 Study Endpoints

2.3.1 Primary Endpoints:

Safety

- Frequency of serious adverse events (SAEs) from Day 1 study vaccination through the end of the study

- Frequency of solicited adverse events (AEs) for 7 days (day of study vaccination and 6 following days) after each dose of study vaccine
- Frequency of unsolicited AEs for 28 days (day of study vaccination and 27 following days) after each dose of study vaccine [including clinically significant aberrant safety monitoring laboratory values on Day 8 reported as AEs]

2.3.2 Secondary Endpoints:

Immunogenicity

- Median type-specific anti-polio serum neutralizing antibody titers at baseline and post-vaccination
- Type-specific anti-polio serum neutralizing antibody Geometric Mean Titer (GMT) at baseline and post-vaccination
- Post-vaccination GMT ratios of type-specific anti-polio serum neutralizing antibody, adjusted for baseline immunity
- Post-vaccination frequency of any fold-rise (titer increased from baseline), a minimum 2-fold rise, and a minimum 4-fold rise (seroconversion), in type-specific anti-polio serum neutralizing antibody response

Fecal Shedding of Study Vaccine Viruses

- Time to cessation of fecal shedding (days) of the vaccine virus, following initial dose
- Proportion of participants shedding type-specific vaccine virus at each post-vaccination stool collection, as assessed by PCR in participants with an exclusive IPV prior vaccination history
- Amount of vaccine virus in each stool sample ($\log_{10}$ $CCID_{50}$ per gram) positive for virus (PCR) in participants with an exclusive IPV prior vaccination history
- Shedding Index of vaccine virus shedding in stool, defined as the mean of $\log_{10}$ $CCID_{50}$ per gram of stool at 7, 14, 21 and 28 days following each dose in participants with an exclusive IPV prior vaccination history
- AUC of vaccine virus shed in stool in participants with an exclusive IPV prior vaccination history

2.3.3 Exploratory Endpoints:

- Time to cessation of fecal shedding (days) of the vaccine virus, following second dose
- Proportion of participants shedding type-specific vaccine virus at each post-vaccination stool collection, as assessed by PCR in participants with an OPV-containing prior vaccination history
- Amount of vaccine virus in each stool sample ($\log_{10}$ $CCID_{50}$ per gram) positive for virus (PCR) in participants with an OPV-containing prior vaccination history
- Shedding Index of vaccine virus shedding in stool, defined as the mean of $\log_{10}$ $CCID_{50}$ per gram of stool at 7, 14, 21 and 28 days following each dose in participants with an OPV-containing prior vaccination history
- AUC of vaccine virus shed in stool in participants with an OPV-containing prior vaccination history

- Neurovirulence of shed vaccine virus from select stool samples as measured by a transgenic mouse neurovirulence test
- Deep sequencing of shed vaccine virus from select stool samples, using NGS
- Serum neutralizing antibody titers against non-vaccine poliovirus types at baseline and post-vaccination

3 STUDY DESIGN

This multicenter trial is the first-in-human assessment of these novel oral poliomyelitis vaccines. It will be an 8-arm, randomized, observer-blind, controlled trial, with Sabin monovalent vaccines serving as the control for each type (see study schema, below). 150 to 230 healthy, adult participants will be recruited. 30 to 40 of 60 to 80 participants with an exclusive IPV prior vaccination history (cohort 1) will be randomized in a 1:1 ratio to study groups 1 and 2 and allocated to receive nOPV1 or mOPV1, and once these study arms are completely accrued, the other 30 to 40 exclusively-IPV vaccinated participants (cohort 3) will be randomized in a 1:1 ratio to study groups 5 and 6 and allocated to receive nOPV3 or mOPV3.

In parallel, 45 to 75 of 150 participants with an OPV-containing prior vaccination history (cohort 2) will be randomized in a 2:1 ratio to study groups 3 and 4 and allocated to receive two doses of nOPV1 or mOPV1, respectively, and once these study arms are completely accrued, the other 45 to 75 participants with an OPV-containing prior vaccination history (cohort 4) will be randomized in a 2:1 ratio to study groups 7 and 8 and allocated to receive two doses of nOPV3 or mOPV3, respectively.

Participants assigned to two doses of vaccine will receive the second dose 28 days following the first dose. Initial vaccination for Cohorts 1 and 2 participants will be limited to no more than 5 participants per day and a total of 10 participants during the first week of vaccination. Participants will be followed until 24 weeks after their Day 1 study vaccination.

Study Schema

Cohort	Group	Prior Vaccination	Number of Participants (Min-Max)	Dosage $CCID_{50}$		Day 1	Day 29
**1**	1	IPV	15-20	$10^{6\,5}$		nOPV1	-
	2		15-20	$\geq 10^{6}$		mOPV1	-
**Subtotal**			30-40				
**2**	3	OPV	30-50	$10^{6\,5}$		nOPV1	nOPV1
	4		15-25	$\geq 10^{6}$		mOPV1	mOPV1
**Subtotal**			45-75				
**3**	5	IPV	15-20	$10^{6\,5}$		nOPV3	-
	6		15-20	$\geq 10^{5.8}$		mOPV3	-
**Subtotal**			30-40				
**4**	7	OPV	30-50	$10^{6\,5}$		nOPV3	nOPV3
	8		15-25	$\geq 10^{5.8}$		mOPV3	mOPV3
**Subtotal**			45-75				
**Total**			150-230				

4 STUDY POPULATION

4.1 Description of Study Population

The study population will include a total of 150 to 230 healthy adults, between the ages of 18 and 45 years, inclusive. Following informed consent, potentially eligible participants will continue to be screened until 150 to 230 adults have been found to be eligible, randomized and vaccinated. Participants withdrawn for any reason before vaccination will be replaced, participants withdrawn for any reason after vaccination will not be replaced. Enrollment into the study will be competitive in that clinical sites will enroll as many subjects as they can, until the overall study enrollment goal is achieved; the total number of trial participants enrolled study-wide does not change.

To be eligible for randomization and vaccination, participants must meet all the inclusion criteria and none of the exclusion criteria for the study. The investigator should always use good clinical judgement in considering a participant's overall eligibility based on the inclusion and exclusion criteria.

4.2 Inclusion Criteria

1. Males or females, from 18 to 45 years of age (inclusive) at the time of enrollment
2. Healthy, as defined by the absence of any clinically significant medical conditions, either acute or chronic, as determined by medical history, physical examination, screening laboratory test results, and clinical assessment of the investigator
3. Willing and able to provide written informed consent prior to performance of any study-specific procedure
4. If female and of childbearing potential*, be not breastfeeding and not pregnant (based on a negative serum pregnancy test at screening and a negative urine pregnancy test during the 24 hours prior to any study vaccination), agreeing to have repeated pregnancy tests prior to any study vaccination, and having practiced adequate contraception** for 30 days prior to first study vaccination and willing to continue using adequate contraception consistently for at least 90 days after the last study vaccination and until cessation of vaccine virus shedding is confirmed
   * Females can be considered not of childbearing potential if they are with current bilateral tubal ligation, occlusion or removal, or post-total hysterectomy, or post-bilateral ovariectomy
   ** Adequate contraception is defined as a contraceptive method with failure rate of less than 1% per year when used consistently and correctly and when applicable, in accordance with the product label, for example:
   - Abstinence from penile-vaginal intercourse
   - Combined estrogen and progesterone oral contraceptives
   - Hormonal (e.g., progestogen) injections
   - Hormonal (e.g., etonogestrel or levonorgestrel) implants
   - Contraceptive vaginal ring
   - Percutaneous contraceptive patches
   - Intrauterine device
   - Intrauterine hormonal system
   - Male condom combined with a vaginal spermicide (foam, gel, film, cream, or suppository), and/or progesterone alone oral contraceptive
   - Monogamous relationship with vasectomized (≥ 180 days prior to enrollment) partner

5. Resides in study area and is able and willing to adhere to all study restrictions and to all study visits and procedures (as evidenced by a signed informed consent form [ICF] and assessment by the investigator)
6. Agrees not to and has no plans to travel outside the United States (US) until confirmation of cessation of vaccine virus shedding in stool at or after the study Day 57 stool collection
7. Able and willing to be contacted by telephone or text, and willing for study staff to leave telephone voice or electronic messages as needed
8. Neutralizing antibody titer ≥ 1:8 for poliovirus type 1 (for participants in cohorts 1 and 2) and ≥ 1:8 for poliovirus type 3 (for participants in cohorts 3 and 4)
9. For Cohorts 1 and 3 only: previously received at least 3 doses of IPV and with no history of receipt of OPV. For Cohorts 2 and 4 only: previously received a primary polio immunization series containing OPV

4.3 Exclusion Criteria

1. Have any condition (medical, psychiatric or behavioral) that, in the opinion of the investigator, would increase the participant's health risks in study participation or would increase the risk of not achieving the study's objectives (e.g., would compromise adherence to protocol requirements or interfere with planned safety and immunogenicity assessments)
2. Receipt of polio vaccine within 12 months before the start of the study
3. Having Crohn's disease or ulcerative colitis or having had major surgery of the gastrointestinal tract involving significant loss or resection of the bowel
4. A known allergy, hypersensitivity, or intolerance to any components of the study vaccines, including all macrolide and aminoglycoside antibiotics (e.g., erythromycin and kanamycin)
5. Any confirmed or suspected immunosuppressive or immunodeficiency condition (human immunodeficiency virus [HIV] infection, or total serum immunoglobulin A (IgA) or immunoglobulin G (IgG) level below the testing laboratory's lower limit of normal [LLN])
6. Administration of any long-acting immune-modifying drugs (e.g., infliximab or rituximab) or the chronic administration (i.e., longer than 14 days) of immunosuppressant drugs (e.g., oral or systemic steroids) or other immune-modifying drugs within 6 months prior to the first vaccine dose or planned use during the study (inhaled and topical steroids are allowed whereas intra-articular and epidural injection/administration of steroids are not allowed)
7. Will have household direct or close professional contact during the study with individuals expected to be immunosuppressed (due to underlying condition or treatments) or individuals who have not yet completed their primary infant polio immunization series (i.e., three doses)
8. Will have household direct or close professional contact during the study with pregnant women
9. Will have household direct or close professional (e.g., neonatal nurses) contact during the study with children less than 2 years of age or with individuals who are encopretic (i.e., infants/toddlers who are not yet toilet trained or other individuals, including adults, with fecal incontinence)
10. Will have professional handling of food, catering, or food production activities during the study
11. Reside in homes with septic tanks
12. Acute illness or fever (body temperature measured orally ≥ 38°C or 100.4°F) at the time of study vaccine administration (once acute illness/fever is resolved, if appropriate, as per investigator assessment, participant may complete screening)
13. Indications of drug abuse or excessive use of alcohol as deemed by the investigator to confound safety assessments or render the participant unable or unlikely to adhere to protocol requirements or provide accurate safety reports

14. Participation in another investigational product (drug or vaccine) clinical trial within 30 days prior to entry in this study or receipt of any such investigational product other than the study vaccine within 30 days prior to the first administration of study vaccine, or planned use during the study period
15. Administration of any vaccine or any intramuscular injection (except seasonal inactivated influenza and COVID-19 vaccines which are prohibited for only 14 days prior to or following each study vaccination) other than the study vaccine within 30 days prior to the first dose of study vaccine or planned administration within 30 days prior to or after any study vaccination.
16. Receipt of transfusion of any blood product or application of immunoglobulins within the 12 weeks prior to the first administration of study vaccine or planned use during the study period
17. Hepatitis B or C virus infection
18. Any hematological# or chemistry** parameter that is out of range of normal†† and is considered clinically significant by the investigator
    #Complete blood count (CBC), includes hemoglobin, hematocrit, white blood cell (WBC) count, neutrophil count, lymphocyte count, eosinophil count, and platelet count
    **Creatinine, alanine transaminase (ALT), total bilirubin
    ††Per the site clinical laboratory's reference ranges. All tests with out of range results that are regarded as clinically significant by the clinician must be repeated and determined to be not clinically significant before any participant can be enrolled.
19. The following hematological or chemistry laboratory results will be considered exclusionary, irrespective of assessment of clinical significance:
    Hemoglobin (Male) < 12.5 g/dL
    Hemoglobin (Female) < 11.0 g/dL
    Neutrophil count < 1,000 cells/mm^3
    Eosinophil count > 650 cells/mm^3
    Platelet count < 125,000 cells/mm^3
    Creatinine > 1.4 mg/dL
    ALT > 1.1 X Upper limit of normal (ULN) ††
    ††Per the site clinical laboratory's reference ranges

5 STUDY PRODUCTS

5.1 Study Vaccine

5.1.1 Product Description

5.1.1.1 nOPV study vaccines

Both nOPVs are live, attenuated polioviruses derived from a modified Sabin type-2 infectious cDNA clone and propagated in Vero cells. Modifications were introduced in the viral nucleotide sequences in part of the 5' UTR to improve the genetic stability of this major attenuating determinant of Sabin type-2, and two modifications were made in the 3D polymerase to further improve stability of the attenuation and reduce recombination. In addition, a key replication element from the 2C coding region was relocated to the 5' UTR to reduce the risk of loss of stabilized 5' UTR through recombination. The cDNA plasmid was then further modified by replacement of the capsid (P1) region of the genome with the capsid from a Sabin-1 (nOPV1) or Sabin-3 (nOPV3) clone, generating chimeric viruses with novel type 2 non-structural regions coupled with Sabin-1 or -3 structural proteins.

For this study, a $10^{6.5 \pm 0.5}$ $CCID_{50}$ dosage is planned for both nOPV1 and nOPV3. The dosage is considered likely to be similar to the actual dosages of Sabin-1 and Sabin-3 released by manufacturers, considering overages above the expiry label claim ($10^{6.0}$ and $10^{5.8}$ $CCID_{50}$) for stability losses as well as process and analytical variability. Moreover, if *in vitro* culture data suggesting a fitness reduction at 37°C compared to the homotypic Sabin strains transfers to human infectivity, this dosage allows a reasonable margin over the historical Sabin monovalent dosage known to be immunogenic ($10^{5.0}$ $CCID_{50}$ [10]) to have a reasonable expectation of responses. The safety of a $10^{6\,5}$ $CCID_{50}$ dose is not considered a concern, as most of the viral exposure for OPV comes from replication *in vivo*, and Sabin strains have been studied at $10^{7.0}$ $CCID_{50}$.

5.1.1.2 Sabin mOPV control vaccines

Both control vaccines are Sabin mOPVs that are constituent components of the WHO prequalified bivalent OPV manufactured by Bio Farma. Bio Farma also manufactures a WHO prequalified mOPV1 for supplemental campaigns. Both mOPV1 and mOPV3 will be manufactured and vialled by Bio Farma for this trial.

In addition to the attenuated poliovirus, the vaccine formulation includes sucrose and may contain trace amounts of not more than 2 mcg erythromycin and not more than 10 mcg kanamycin.

5.1.2 Manufacturer

All vaccines to be used in this trial, both the nOPV study vaccines and the Sabin mOPV control vaccines are manufactured by Bio Farma, Indonesia.

5.1.3 Presentation and Formulation

5.1.3.1 nOPV study vaccines

In addition to the attenuated poliovirus, the vaccine formulation includes sucrose, acetic acid or $NaHCO_3$, and Basal Medium Eagle (BME) solution. The nOPVs are clear solutions, yellow to pink in color. The multidose vaccine vials (20 doses) contain approximately 2.2 mL of vaccine and are packaged with the dropper that is attached for administration directly from the vial. Each 0.1 mL (2 drops) dose of vaccine contains approximately $10^{6\,5}$ $CCID_{50}$. In this study, only a single dose is to be administered from each multidose vial provided.

5.1.3.2 Sabin control vaccines

The control vaccines are clear and light yellow to light red. The multidose vaccine vials (20 doses) contain approximately 2.2 mL of vaccine and are packaged with the dropper that is attached for administration directly from the vial. The mOPV1 control vaccine contains $\geq 10^{6.0}$ $CCID_{50}$ per 0.1 mL (2 drops) dose, and the mOPV3 control vaccine contains $\geq 10^{5.8}$ $CCID_{50}$ per 0.1 mL (2 drops) dose. In this study, only a single dose is to be administered from each multidose vial provided.

5.1.4 Stability and Storage

Both nOPVs and Sabin control mOPVs are to be transported and stored at ≤-20°C and thawed prior to administration. Unopened vaccine vials may be thawed and kept at 2-8°C for up to 14 days prior to administration, although it is preferred to administer the vaccines the same day as they are thawed. The thawed vaccines should not be left at ambient temperature for more than one hour and must not be refrozen.

5.2 Dose Preparation and Administration

Dose preparation, as described in detail in the Pharmacy Manual, will be carried out by a qualified unblinded research pharmacist and witnessed by another unblinded study staff member. The unblinded pharmacist will affix the dropper to the vial and apply an overlay on the vial on which the participant ID is printed, masking the identity of the contents of the vial. Study nOPVs and control mOPVs will be dispensed directly from the vials by unblinded staff who will dispense 2 drops of study vaccine directly from the dropper into the participant's mouth. To maintain the observer-blind study design, these unblinded staff will not be involved in any other study assessments (e.g., safety assessments) of the participants. Any vomiting following study vaccination during the post-vaccination observation period, including the precise time of the onset of vomiting, will also be recorded. There will be no vaccine redosing to compensate for any vomited vaccine.

5.3 Accountability and Disposal

The site pharmacist is required to maintain complete records of all study vaccines received from PATH and will be responsible for maintaining an accurate record of the randomization codes, inventory, and an accountability record of vaccines for this study. The site pharmacist will also be responsible for ensuring the security of these documents. Partially used vials will not be used for human administration or for in vitro experimental studies. At the end of the study, the site will receive instruction from PATH regarding the final disposition of any remaining study vaccines.

6 STUDY PROCEDURES

6.1 Recruitment

Study participants will be recruited from the local community and reflect the demographics of the community yet keeping with the strict eligibility criteria. The sites selected have ample prior experience in the recruitment of healthy adult participants from their local community for participation in preventive vaccine clinical trials. Information regarding this trial may be provided to potential participants who have previously participated in vaccine trials conducted at the site. Other forms and mechanisms of recruitment may also be used. The local Institutional Review Board/Independent Ethics Committee (IRB/IEC) will approve all materials prior to use. Careful recruitment and communication about all aspects of the study will be critical to ensure eligible participants who eventually participate in the trial are committed to participate for the full length of the study. It is anticipated that the sites selected will recruit the required study sample size over a period of approximately sixteen months.

6.2 Study Visits

A schedule of study visits and evaluations for the participants with an exclusive IPV vaccination history (single dose study groups) and participants with an OPV-containing vaccination history (two dose study groups) can be found in Appendix 1.

6.2.1 Screening – Day -90 to Day 1

After the site Principal Investigator (PI) or designee has obtained informed consent from participants, the participant will be considered enrolled, and the following procedures will be completed during screening to determine study eligibility and may occur over multiple screening visits. Additional screening visits may be scheduled for any follow up, as needed, but are not required. At the screening visit(s), the site PI or designee will provide prospective participants a detailed description of the study objectives and study

participation requirements, as well as potential health risks and benefits associated with study participation. Baseline data are obtained during the screening period, which has an allowable window up to no more than 90 days prior to Day 1, the day of first vaccination. It is anticipated that it may take up to two weeks to receive and evaluate all screening laboratory tests to confirm eligibility and allow for randomization. All inclusion/exclusion criteria must be assessed from data obtained within the screening period, unless otherwise specified in the eligibility criteria.

After study information has been provided and the appropriate informed consent has been obtained, the following procedures are performed during the screening period before the Day 1 Visit:

- Confirm written informed consent has been obtained and solicit/discuss any remaining questions the participant may have
- Assign participant ID once study specific consent form has been signed
- Obtain demographic and contact (e.g., address, telephone, email) information
- Obtain medical history
- Obtain history of prior COVID-19 vaccinations and ongoing concomitant medication use and any prior use that impacts eligibility, including poliovirus vaccination history (documented vaccination records is preferred; however, if not obtainable, to ascertain if vaccination history can be reasonably established by history of lifelong residency locations and polio vaccines that were available for routine immunizations at the relevant time periods in these locations)
- Measure height and weight
- Perform complete physical examination (including vital signs [VS])
- Collect venous blood samples (approximately 45 mL) for
    - CBC, including hemoglobin, hematocrit, WBC count, neutrophil count, lymphocyte count, eosinophil count, and platelet count
    - Serum chemistries to include creatinine, ALT, and total bilirubin
    - Human immunodeficiency viruses 1 and 2 (HIV 1/2) infection testing
    - Hepatitis B virus surface antigen (HBsAg)
    - Hepatitis C virus antibody (HCV Ab) and if positive, HCV PCR
    - Total IgG and total IgA
    - Serum pregnancy test for females of childbearing potential (FOCB)
    - Neutralizing antibodies to type-specific polioviruses (in addition to sample sent to the Polio and Picornavirus Laboratory Branch at the CDC, a sample will be sent to a commercial qualified laboratory, Quest Diagnostics, for screening purposes [eligibility determination] only

After confirmation of eligibility, participants can be randomized prior to Day 1.

6.2.2 First Vaccination Visit – Day 1 Visit

Prior to Vaccine Administration

- Review concomitant medications
- Review inclusion/exclusion criteria and ensure continued eligibility for study vaccination, including absence of symptoms that may confound later safety and solicited AE assessments
- If any interim symptoms, perform a targeted physical examination
- Measure vital signs

- For females of childbearing potential (FOCB), assess and document continuing use of contraceptives and perform urine pregnancy test
- Collect venous blood samples (approximately 15 mL) for
    - CBC, including hemoglobin, hematocrit, WBC count, neutrophil count, lymphocyte count, eosinophil count, and platelet count
    - Serum chemistries to include creatinine, ALT, and total bilirubin
- If not done earlier than Day 1, once eligibility is confirmed, randomize participant

Clinical safety laboratory test results used to determine participant eligibility will be those obtained at screening, but the Day 1 test results will be used as baseline for analyses.

Vaccine Administration

- If an eligible participant is disqualified prior to study vaccine administration (e.g., participant withdraws consent or the investigator reconsiders and disqualifies for a documented reason), another eligible participant will be selected for randomization in place of the disqualified participant
- The vaccine will be administered – 2 drops (0.1 mL) by unblinded study staff directly from the masked vaccine vial's dropper into the participant's mouth

Post Vaccination

- Observe participant for at least 30 minutes post-vaccination with medical treatment readily available in case of any immediate hypersensitivity reactions
- Assess and document any interim adverse events, and if a participant requires further on-site observation, additional site or clinical assessments may be completed as needed
- Provide participant with supplies (including a thermometer and a memory aid) to record solicited AE information and instruct the participant in their use
- Prior to discharge from the clinic, once the required observation period is over, the Day 1 solicited AE memory aid entries may be completed by the participant and reviewed on site to further support good comprehension
- Provide participants in Cohorts 1 or 3 with instructions and supplies to collect stool specimens within the appropriate windows on study Days 3, 5, and 8, and participants in Cohorts 2 or 4 to collect a stool specimen within the appropriate windows 7 days after vaccination (study Day 8), prior to their next scheduled visit. Cohorts 1 and 3 participants may contribute additional stool samples between study Days 8 and 29, in addition to the nominal study stool sampling days, if available. These optional samples will be used to increase the number of samples available for the exploratory genetic stability evaluation. Similar to future use samples, participants' refusal to provide the additional stool sampling timepoints should not affect their eligibility to enroll into the study.
- Provide participant clear guidance on appropriate hygienic measures and supplies to minimize contact and contamination during the stool collection process (e.g., collection kit, sanitizer, disinfectant, gloves) and minimize risk of transmission of vaccine virus to others. Ask participant to describe these measures ('teach back method') for hand washing and stool collection before leaving the clinic and correct any errors
- Provide participant information about storage and transport of stool specimens to the study site
- Schedule next study visit (Day 8 Visit) and instruct participant to contact study staff for any symptoms of concern, and irrespective, for any solicited AE defined greater than moderate in severity, and ensure participant has appropriate contact details for the study team

- Once all study related procedures are complete and the investigator determines that the participant's condition is acceptable, the participant may be discharged from the study clinic

6.2.3 Day 8 Visit, 7 (+1) days after the study vaccination – Clinic follow-up visit

At the Day 8 Visit, the participant will return to the study clinic for the following procedures:

- Collect any pending stool samples from the participant
- Review with participant the completed memory aid and ensure that the participant has correctly interpreted the instructions in completing the memory aid; any required corrections to be clearly documented
- Collect any new AEs since Day 1 Visit that have not already been captured and review any AEs that are currently ongoing
- Collect any new concomitant medications since Day 1 Visit that have not already been captured and review any concomitant medications that are currently ongoing
- If the participant reports any intervening symptom or AE since the previous visit, perform a targeted physical examination, which is to include vital signs
- Any topics or new information considered by investigator to be important to continued informed consent to be shared with the participant
- Collect venous blood samples (approximately 15 mL) for
    - CBC, including hemoglobin, hematocrit, WBC count, neutrophil count, lymphocyte count, eosinophil count, and platelet count
    - Serum chemistries to include creatinine, ALT, and total bilirubin
- Provide participants in Cohorts 1 or 3 with instructions and supplies to collect stool specimens within the appropriate windows on study Days 10, 15, 22, and 29, and participants in Cohorts 2 or 4 to collect stool specimens on study Days 15, 22, and 29 prior to their next scheduled visit. Cohorts 1 and 3 participants may contribute additional stool samples between study Days 8 and 29, in addition to the nominal study stool sampling days, if available. These optional samples will be used to increase the number of samples available for the exploratory genetic stability evaluation. Similar to future use samples, participants' refusal to provide the additional stool sampling timepoints should not affect their eligibility to enroll into the study.
- Provide participant clear guidance on appropriate hygienic measures and supplies to minimize contact and contamination during the stool collection process (e.g., collection kit, sanitizer, disinfectant, gloves) and minimize risk of transmission of vaccine virus to others
- Provide participant information about storage and transport of stool specimens to the study site
- Schedule next study visit (Day 29 Visit) and instruct participant to contact study staff for any symptoms of concern, and ensure participant has appropriate contact details for the study team

6.2.4 Day 29 Visit, 28 (+2) days after the study vaccination – Clinic follow-up visit

At the Day 29 Visit, the participant will return to the study clinic for the following procedures:

- Collect any pending stool samples from the participant
- Collect any new AEs since Day 8 Visit that have not already been captured and review any AEs that are currently ongoing
- Collect any new concomitant medications since Day 8 Visit that have not already been captured and review any concomitant medications that are currently ongoing

- If the participant reports any intervening symptom or AE since the previous visit, perform a targeted physical examination, which is to include vital signs
- Any topics or new information considered by investigator to be important to continued informed consent to be shared with the participant
- Collect venous blood sample (approximately 10 mL) for neutralizing antibodies to type-specific polioviruses

For participants in Cohorts 1 or 3, who were randomized to receive only a single dose of study vaccine:

- Provide participant with instructions and supplies to collect stool specimens within the appropriate windows on study Days 36, 43, 50, and 57 prior to their next scheduled visit
- Provide participant clear guidance on appropriate hygienic measures and supplies to minimize contact and contamination during the stool collection process (e.g., collection kit, sanitizer, disinfectant, gloves) and minimize risk of transmission of vaccine virus to others. Ask participant to describe these measures ('teach back method') for hand washing and stool collection before leaving the clinic and correct any errors
- Provide participant information about storage and transport of stool specimens to the study site
- Schedule next study visit (Day 169 Visit), which will occur via telephone, and instruct participant to contact study staff for any symptoms of concern and ensure participant has appropriate contact details for the study team
- Inform participant that if cessation of vaccine virus shedding is not confirmed by Day 57 stool sample, they will be asked to continue with stool collections and the associated protocol restrictions until they are informed that cessation of shedding has been confirmed

If poliovirus shedding is detected by PCR on one of the last two scheduled stool samples, stool sample collection duration for that participant will be extended. As soon as the shedding results are known (anticipated approximately two weeks after the last stool sample provided for evaluation) the participant will be asked to collect additional stool samples after the last per protocol sample obtained over a period no shorter than 24 hours, and to repeat this until shedding is PCR-negative for poliovirus on two consecutive stool samples collected. They will be provided additional stool collection kit for each sampling day.

If the last stool sample is missing the participant will be asked to provide a new sample as soon as possible in order to determine the need for further stool sample collection until poliovirus shedding is PCR negative on two consecutive stool samples.

For participants in Cohorts 2 or 4, who were randomized to receive two doses of study vaccine:

- Review inclusion/exclusion criteria and ensure continued eligibility for study vaccination, including absence of symptoms that may confound later safety and solicited AE assessments
- Measure vital signs
- For females of childbearing potential (FOCB), assess and document continuing use of contraceptives and perform urine pregnancy test
- If eligibility criteria are still met, 2 drops (0.1 mL) of study vaccine will be administered by unblinded study staff directly from the masked vaccine vial's dropper into the participant's mouth
- Observe participant for at least 30 minutes post vaccination with medical treatment readily available in case of any immediate hypersensitivity reactions

- Assess and document any interim adverse events, and if a participant requires further on-site observation, additional site or clinical assessments may be completed as needed
- Provide participant with supplies (including a thermometer and a memory aid) to record solicited AE information and review its use again
- Prior to discharge from the clinic, once the required observation period is over, the first day solicited AE memory aid entries may be completed by the participant and reviewed on site to further support retained comprehension
- Provide participant with instructions and supplies to collect a stool specimen 7 days after second vaccination (study Day 36) prior to their next scheduled visit
- Provide participant clear guidance on appropriate hygienic measures and supplies to minimize contact and contamination during the stool collection process (e.g., collection kit, sanitizer, disinfectant, gloves) and minimize risk of transmission of vaccine virus to others
- Provide participant information about storage and transport of stool specimen to the study site
- Schedule next study visit (Day 36 Visit) and instruct participant to contact study staff for any symptoms of concern, and irrespective, for any solicited AE defined greater than moderate in severity, and ensure participant has appropriate contact details for the study team
- Once all study related procedures are complete and the investigator determines that the participant's condition is acceptable, the participant may be discharged from the study clinic

6.2.5 Day 36 Visit, 7 (+1) days after the second study vaccination – Clinic follow-up visit

Only for those in Cohorts 2 or 4, who have received a second study vaccination

At the Day 36 Visit, the participant will return to the study clinic for the following procedures:

- Review with participant the completed memory aid and ensure that the participant has correctly interpreted the instructions in completing the memory aid; any required corrections to be clearly documented
- Collect any new AEs since Day 29 Visit that have not already been captured and review any AEs that are currently ongoing
- Collect any new concomitant medications since Day 29 Visit that have not already been captured and review any concomitant medications that are currently ongoing
- If the participant reports any intervening symptom or AE since the previous visit, perform a targeted physical examination, which is to include vital signs
- Any topics or new information considered by investigator to be important to continued informed consent to be shared with the participant
- Provide participant with instructions and supplies to collect stool specimens within the appropriate windows on study Days 43, 50, and 57 prior to their next scheduled visit
- Provide participant clear guidance on appropriate hygienic measures and supplies to minimize contact and contamination during the stool collection process (e.g., collection kit, sanitizer, disinfectant, gloves) and minimize risk of transmission of vaccine virus to others
- Provide participant information about storage and transport of stool specimens to the study site
- Schedule next study visit (Day 57 Visit) and instruct participant to contact study staff for any symptoms of concern, and ensure participant has appropriate contact details for the study team

6.2.6 Day 57 Visit, 28 (+2) days after the second study vaccination – Clinic follow-up visit

Only for those in Cohorts 2 or 4, who have received a second study vaccination

At the Day 57 Visit, the participant will return to the study clinic for the following procedures:

- Collect any new AEs since Day 36 Visit that have not already been captured and review any AEs that are currently ongoing
- Collect any new concomitant medications since Day 36 Visit that have not already been captured and review any concomitant medications that are currently ongoing
- If the participant reports any intervening symptom or AE since the previous visit, perform a targeted physical examination, which is to include vital signs
- Any topics or new information considered by investigator to be important to continued informed consent to be shared with the participant
- Collect venous blood sample (approximately 10 mL) for neutralizing antibodies to type-specific polioviruses
- Schedule next study visit (Day 169 Visit), which will occur via telephone and instruct participant to contact study staff for any symptoms of concern and ensure participant has appropriate contact details for the study team
- If cessation of vaccine virus shedding has not been confirmed by Day 57 stool sample, ask participant to continue with stool collections and the associated protocol restrictions until informed that cessation of shedding has been confirmed. Ensure that cessation of vaccine virus shedding has been ultimately confirmed.

If poliovirus shedding is detected by PCR on one of the last two scheduled stool samples, stool sample collection duration for that participant will be extended. As soon as the shedding results are known (anticipated approximately two weeks after the last stool sample provided for evaluation) the participant will be asked to collect additional stool samples after the last per protocol sample obtained over a period no shorter than 24 hours, and to repeat this until shedding is PCR-negative for poliovirus on two consecutive stool samples collected. They will be provided additional stool collection kit for each sampling day.

If the last stool sample is missing the participant will be asked to provide a new sample as soon as possible in order to determine the need for further stool sample collection until poliovirus shedding is PCR negative on two consecutive stool samples.

6.2.7 Day 169 Telephone Visit, 168 (+14) days after the initial study vaccination – Final Study Visit

At Day 169, the participant is expected to be available for a telephone follow-up for the following procedures:

- Collect any new SAEs reported since previous Visit that have not already been captured and review SAEs that are currently ongoing
- Collect any new SAE associated concomitant medications reported since previous Visit that have not already been captured and review any SAE associated concomitant medications that are currently ongoing
- Instruct participant to come to the clinic for a targeted physical exam, including vital signs, if they report any intervening symptom or AE since the previous visit that is of clinical concern

6.2.8 Unscheduled Visits

Unscheduled visits (those between regularly scheduled follow up visits) may be performed at participant request or as deemed necessary by the investigator at any time during the study. All unscheduled contacts and visits will be documented in the participant's study records and on applicable case report forms.

6.3 Discontinuation of Vaccination or Study Procedures

Participants have the right to decline study vaccinations or procedures for any reason and at any time during the study. If a participant declines further vaccination or study procedures, this will be recorded as a study deviation and the reason will be clearly documented in the source document. The participant will be encouraged to complete the remaining applicable safety related follow-ups and immunogenicity blood draw. If the participant does not wish to remain in the study by declining any follow-up or procedures, the participant can choose to withdraw consent and be withdrawn from the study.

Discontinuation from further vaccination may be at the discretion of the investigator or Protocol Safety Review Team (PSRT), if determined to be in the participant's best interest to do so (e.g., safety concern). In addition, participants will be discontinued from further vaccination for the following reasons:

- Pregnancy
- Ineligibility (either arising during the trial or retrospectively having been overlooked at screening)
- Significant or repeated non-compliance with trial requirements
- An adverse event which requires discontinuation of study vaccination or results in inability to continue to comply with trial procedures
- Intercurrent illness or diseases or medical treatment that occur during the trial and might influence the study results or ability to continue to comply with trial procedures

6.4 Withdrawal from Study

Participation in the study is strictly voluntary. Participants have the right to withdraw from the study at any time and for any reason, without penalty. The investigator or PATH may, at their discretion, withdraw a participant from continuing in the study if it is considered to be in the participant's best interest to do so, or if the participant is not willing or able to comply with the study requirements. The reason for withdrawal will be documented in source document and relevant case report form (CRF).

In the event of withdrawal from study, reasonable efforts should be made to conduct the following procedures:

- Review memory aid if still in use prior to withdrawal
- Update any ongoing AE/SAEs that remain ongoing at time of participant's last visit prior to withdrawal
- Query about AEs/SAEs and concomitant medications if the interval between the participant's last visit and the time of withdrawal is within the protocol defined reporting period
- Physical examination including vital signs
- Obtain any interim stool samples the participant may have collected for the study
- Collect venous blood samples for safety laboratory testing if withdrawal occurs before Day 8 Visit, and for immunological testing if withdrawal occurs before Day 57 Visit
- Update contact information

All participants who withdraw from the study following study vaccination and in whom cessation of vaccine virus shedding has not been confirmed, will be required to continue providing stool specimens for analysis and continue with requirements for mitigating the risk of fecal transmission until cessation of

shedding is confirmed. If a participant does not adhere to the required provision of stool specimens to enable confirmation of cessation of shedding, the investigator shall notify the relevant local health department.

6.5 Loss to Follow-up

To avoid participants being lost to follow-up, participants will be reminded by email or text message within a few days before scheduled visit. In the event of a missed visit, participants will be contacted by phone, email, or text message the next business day following the missed visit. Any participant who fails to attend the final study visit will be classified as lost to follow-up. In addition, a participant who cannot be located or fails to respond after three attempted contacts and has missed two consecutive visits (other than those scheduled primarily to provide stool samples) will be considered lost to follow-up. Efforts to contact the participant will be documented in source documents. There will be no replacement of participants who are lost to follow-up.

6.6 Use of Concomitant Medications During the Study

Prior to study start, participants will be encouraged to receive Coronavirus Disease 2019 (COVID-19) vaccines, and receipt of COVID-19 vaccine prior to study will be reported in the Concomitant Medication CRF. At each study visit, the investigator will ask whether the participant has taken any prescription or over the counter medication since the last visit.

Any medication (includes vaccines not administered as part of study) administered to a participant at any time between the Day 1 visit and 28 days following each study vaccination during the study period must be recorded on source documents and the CRF with generic and/or trade name, indication, dosage, regimen, route of administration, and start and end dates. Between the end of these periods and the final study visits only concomitant medications associated with an SAE need to be recorded in the CRF.

The following concomitant medications/vaccinations are prohibited during the study; however, they must not be withheld by the treating physician if clinically indicated to treat a participant (e.g., response to epidemic, or post-exposure tetanus or rabies vaccine prophylaxis):

- Any investigational drug or vaccine throughout the duration of the study
- Any immunosuppressant or immune-modifying drug (includes systemic steroids) throughout the duration of the study
- Any immunoglobulin or blood product through study Day 57
- Any vaccine other than the study vaccines through study Day 57, which are prohibited for 30 days prior to or following each study vaccination (except for seasonal inactivated influenza and COVID-19 vaccines, which are prohibited for only 14 days prior to or following each study vaccination)
- Any intramuscular injection (except seasonal inactivated influenza and COVID-19 vaccines which are prohibited for only 14 days prior to or following each study vaccination) for 30 days prior to or following each study vaccination

Note: Medication (e.g., antipyretic) should not be recommended prophylactically prior to study vaccine administration to prevent the onset of any post study vaccine administration symptoms, although may be administered after study vaccine administration as clinically indicated.

Use of any prohibited medication must be recorded in the CRF and as such participant's inclusion in the Per Protocol Population will be evaluated on a case by case basis.

6.7 Emergency Unblinding

In the event of a medical emergency, the investigator may request that the blind be broken for the participant experiencing the emergency if knowledge of the participant's study vaccine assignment may influence the participant's clinical care. Prior to unblinding, the investigator is encouraged (to the extent possible, without jeopardizing the participant's health) to contact the study medical monitor to discuss the decision to break the blind. Unblinding will occur through the secure interactive web response system (IWRS), to which there will be 24-hour access. Documentation of the unblinding event will be captured by the IWRS. The investigator will be expected to provide the rationale for the necessity of unblinding based on the reasonable expectation that knowledge of the participant's treatment assignment will have a meaningful impact on the participant's medical care in the short term. If a participant's treatment assignment is unblinded, the participant will remain in the study and continue with protocol-defined follow-up evaluations, including provision of biological samples, but will not receive further study vaccinations. The decision to unblind will be recorded in the participant's study record and communicated to the IRB/IEC and other regulatory bodies, as required.

6.8 Management of Birth Control and Pregnancy During Study

Contraception status is assessed and documented prior to enrollment and each study visit for female participants who are of childbearing potential. Prior to enrollment and at each vaccination visit, staff will ask volunteers to confirm their use of adequate contraception methods if they are able to become pregnant and remind participants about the need to continue using adequate contraception consistently for at least 90 days after the last study vaccination and until cessation of vaccine virus shedding is confirmed. If a female participant becomes pregnant following randomization, no further study vaccines will be administered to that participant, and she will be encouraged to complete remaining visits and study procedures unless medically contraindicated. The investigator is required to notify Emmes within 24 hours of knowledge of a pregnancy. Any participant who becomes pregnant during the period between first vaccination and the last study visit will continue to be followed for pregnancy outcome, even if birth occurs after the scheduled end of the study for the participant, if possible. The pregnancy and its outcome will be reported on the Pregnancy CRF.

6.9 Clinical Assessments

Vital Signs (VS)

- For vaccination visits, the site must measure oral temperature in degrees Celsius or Fahrenheit (recorded to the nearest 0.1 degree) using the same thermometer that will be used subsequently by the participant to measure oral temperature during the post-vaccination period for assessing reactogenicity. For all non-vaccination visits, sites may record temperature using any route (aural, axillary, tympanic, temporal, etc.), and document the method used.
- Respiratory rate will be recorded in breaths per minute.
- Pulse rate in beats per minute will be measured by automated device or manually.
- Non-invasive systolic and diastolic blood pressure will be measured in millimeters of mercury (Hg) by automated device or manually.
- Vital signs will be taken during all targeted physical exams

Height and Weight

- Height will be measured and recorded to the nearest centimeter (cm).

- Weight will be measured in kilogram (kg) and recorded to the nearest 0.1 kg.

Physical Examination

Complete physical examination will include assessment of vital signs, head, eyes, ears, nose, oropharynx, neck, respiratory/pulmonary, cardiovascular, abdomen, musculoskeletal, lymph nodes (neck, supraclavicular, axillary, inguinal), extremities, skin, and neurological.

Targeted Physical Examination

A focused physical examination based on any interim symptoms/AEs reported by the participant. Whenever required, to always include an assessment of vital signs.

Medical History

A comprehensive medical history will be collected at screening, including details of any previous vaccinations in the past 30 days, any history of reactions to vaccinations, participation in clinical trials (especially clinical trials of vaccines), prior surgery or hospitalization, allergy to food/drugs, current medication and history of any chronic or recurrent medical conditions. Detailed poliovirus vaccination history to be collected, including requests to review poliovirus vaccination records. If records not obtainable, to ascertain if vaccination history can be reasonably established by history of lifelong residency locations and polio vaccines that were available for routine immunizations at the relevant time periods in these locations.

7 LABORATORY EVALUATIONS/REQUIREMENTS

7.1 Sample Collection, Distribution and Storage

Blood samples to evaluate vaccine safety will be obtained and processed at the clinical trial site and transported to each site's designated laboratory for clinical testing.

Serum specimens collected for assessment of immunogenicity will be separated into aliquots per study specific process and stored at ≤-20° C before being shipped to the Polio and Picornavirus Laboratory Branch at the United States Centers for Disease Control and Prevention (CDC). For screening purposes (eligibility determination) only, a sample will be shipped to a commercial qualified laboratory, Quest Diagnostics. Samples will be stored in controlled-temperature freezers, with backup power supply to assure proper sample storage.

Stool samples to assess presence of poliovirus and genetic stability of shed virus will be processed at the clinical trial site in a Biosafety Level 2 (BSL-2) laboratory and stored at ≤-20°C before being transported to the Polio and Picornavirus Laboratory Branch at the CDC or Viroclinics Biosciences B.V., Rotterdam, The Netherlands.

After either reference laboratory (CDC or Viroclinics) completes a successful test on any serum or stool sample that has a back-up sample in storage, the laboratory will destroy any of the remaining sample used for the testing within six months.

Details as to how blood and stool specimens should be collected, processed, aliquoted, labeled, stored, and shipped will be provided in the study-specific Laboratory Manual.

7.2 Clinical Laboratory Tests

Protocol mandated clinical screening and safety laboratory tests will be conducted in real time by site local laboratories that are properly accredited and subscribed to a proficiency testing program. These tests include:

- CBC: hemoglobin, hematocrit, WBC count, neutrophil count, lymphocyte count, eosinophil count, and platelet count, performed at screening, and at Day 1 and Day 8 visits
- Serum Chemistry: ALT, total bilirubin, and creatinine, performed at screening, and at Day 1 and Day 8 visits
- Pregnancy test: Serum β-HCG at screening, then urine β-HCG prior to each study vaccination (conducted on site)
- Human immunodeficiency viruses 1 and 2 (HIV 1/2) infection testing performed at screening
- Hepatitis B virus surface antigen performed at screening (HBsAg)
- Hepatitis C virus antibody (HCV Ab), if positive, HCV PCR, performed at screening
- Total IgG and IgA performed at screening

Laboratory results will be reviewed promptly by the investigator. Participants will be notified of any clinically significant abnormalities. If clinically significant abnormalities are identified during screening, participants will be referred to their primary health provider or appropriate medical center. If identified during the study, participants may be asked to return to the study site for further evaluation, including clinical evaluation and repeat laboratory testing (e.g., monitoring for resolution or stabilization) as warranted. Any test may be repeated for test results determined to be spurious by the investigator (e.g., following improper specimen collection) or if a valid test cannot be performed on the original blood collected (e.g., collection tube broken during transportation).

The immunological assays to be performed include:

- Serum neutralizing antibodies to type-specific polioviruses at screening and 28 days after each vaccine dose, to be performed at the Polio and Picornavirus Laboratory Branch at the CDC (for screening purposes [eligibility determination] only, baseline assays will be performed at a commercial qualified laboratory, Quest Diagnostics)

7.3 Assessment of Shed Poliovirus

- Multiplex real-time PCR for identification of type-specific poliovirus in stool, to be performed at the Polio and Picornavirus Laboratory Branch at the CDC
- $CCID_{50}$ for quantification of type 1 or 3 poliovirus in stool, to be performed at the Polio and Picornavirus Laboratory Branch at the CDC
- Quantitative PCR for quantification of type 1 or 3 poliovirus in stool may be performed at Viroclinics Biosciences B.V.
- Neurovirulence of fecally shed vaccine virus as assessed by a transgenic mouse neurovirulence test (TgmNVT), to be performed at Viroclinics Biosciences B.V.
- NGS of fecally shed vaccine virus, to be performed by Viroclinics Biosciences B.V.

The operational definition of cessation of vaccine virus shedding in stool is 2 consecutive stool specimens determined to be negative by PCR obtained over a period no shorter than 24 hours.

7.4 Future Use of Stored Samples

Participants will be informed about and asked whether they agree to the long-term storage of their biological (stool and serum) specimens for use in future research as part of the informed consent process. Participants need not agree to this long-term storage component of the study to otherwise be consented to take part in the study. Participants who do agree to this will be consenting to the indefinite long-term storage of their remaining biological samples and data for use in future research, which unless further consent and IRB/IEC approvals are obtained, will be restricted to further knowledge about polio, the study vaccines, immune responses to the study vaccines, and the development of assays to measure these immune responses. No tests will be performed on the participant's genome. At study closure, these additional remaining samples will continue to be stored at the Polio and Picornavirus Laboratory Branch at the CDC, and/or if required, another PATH-designated biorepository that meets all current poliovirus containment requirements.

7.5 Biohazard Containment

As transmission of blood-borne pathogens can occur through contact with contaminated needles, blood, and blood products, appropriate blood and secretion precautions will be employed by all personnel in the drawing of blood and shipping and handling of all specimens for this study as recommended by the CDC. Risk mitigation procedures for the prevention of transmission of shed vaccine poliovirus during the collection, processing, storage, and transport of stool specimens will also follow CDC guidelines as stated in this protocol and detailed in the study-specific Laboratory Manual. All biological specimens will be transported using packaging mandated by United States Code of Federal Regulations (CFR) 42 Part 72. All dangerous goods materials, including diagnostic specimens and infectious substances, must be transported according to instructions detailed in the International Air Transport Association (IATA) Dangerous Goods Regulations. Biohazardous waste will be contained according to institutional, transportation/carrier, and all other applicable regulations.

All protocol specimens will be shipped using packing that meets requirements specified by the International Air Transport Association Dangerous Goods Regulations for UN 3373, Biological Substance, Category B, and Packing Instruction 650. Culture isolates, if obtained in this study, are to be shipped as specified for UN 2814 Category A Infectious Substances.

8 SAFETY ASSESSMENT AND REPORTING

8.1 Definitions

8.1.1 Adverse Event (AE)

An adverse event (AE) is any untoward medical occurrence in a participant after administration of the study vaccine and that does not necessarily have a causal relationship with the study vaccine. An AE can therefore be any unfavorable and unintended sign (including clinically significant abnormal laboratory finding), symptom, physical examination, or disease temporally associated with the use of the study vaccine, whether or not related to the study vaccine. This definition includes exacerbations of pre-existing conditions. Stable pre-existing conditions that do not change in nature or severity during the study are not considered AEs; however, these should be reported as part of the medical history.

Solicited AEs are pre-specific AEs that are common or known to be associated with vaccination that are actively monitored as potential indicators of vaccine reactogenicity. Investigators will not be required to

assess causality of solicited AEs if the onset is during the solicitation periods. Solicited AEs with onset after the solicitation period will be captured as unsolicited AEs.

For this trial, solicited AEs will be assessed on the day of study vaccination and for the 6 following days after each dose of study vaccine. Participants will be provided a memory aid to record the presence and severity or absence of solicited AEs. The memory aid may be accessed by smartphone or any device that connects to the internet. Solicited AEs with onset during the solicitation period that persist beyond the solicitation period will continue to be captured as solicited AEs.

The following specific solicited AEs will be monitored for this trial (Cohort 1):

- Fever (oral temperature ≥ 38.0°C or 100.4°F). (The temperature comparison table with the different methods used can be found in the study MOP.)
- Chills
- Fatigue
- Headache
- Muscle aches/Myalgias
- Joint aches/Arthralgias
- Nausea
- Vomiting
- Abdominal pain
- Diarrhea

The following specific solicited AEs will be monitored for this trial (F Cohorts 2 and 3):

- Fever (axillary temperature ≥ 37.5°C)
- Vomiting
- Diarrhea
- Irritability
- Decreased feeding
- Decreased activity

Unsolicited AEs are any AEs reported spontaneously by the participant, observed by the study personnel during study visits or those identified during review of medical records or source documents.

In the absence of a diagnosis, abnormal physical examination findings or abnormal clinical safety laboratory test results that are assessed by the investigator to be clinically significant will be reported as an AE.

8.1.2 Adverse reaction / Suspected Adverse Reaction (21CFR Part 312.32)

Suspected adverse reaction is any adverse event for which there is a reasonable possibility that the vaccine caused the adverse event. For the purposes of IND safety reporting, "reasonable possibility" means there is evidence to suggest a causal relationship between the vaccine and the adverse event. Suspected adverse reaction implies less certainty about causality than adverse reaction, which means any adverse event caused by a vaccine.

Adverse reaction is any adverse event caused by the vaccine. Adverse reactions are a subset of suspected adverse reactions for which there is reason to conclude that the vaccine caused the event.

Unexpected adverse event or unexpected suspected adverse reaction refers to an event or reaction that is not listed in the IB or is not listed at the specificity or severity that has been observed.

8.1.3 Serious Adverse Event (SAE)

Serious adverse event is any adverse event that results in any of the following outcomes:

- Death
- Is life-threatening (life-threatening means that the study participant was, in the opinion of the investigator or PATH, at risk of death at the time of the event; it does not refer to an event which hypothetically might have caused death if it were more severe)
- Requires inpatient hospitalization or prolongation of existing hospitalization
- Results in persistent or significant incapacity or substantial disruption of the ability to conduct normal life functions
- Congenital anomaly or birth defect
- Important medical event that may not result in one of the above outcomes, but based upon appropriate medical judgment, may jeopardize the health of the study participant, and require medical or surgical intervention to prevent one of the outcomes listed above

Suspected unexpected serious adverse reaction (SUSAR) is any suspected adverse reaction that is both unexpected and serious.

8.2 Reporting Period and Parameter

Safety events are reported from the time of signing the ICF through each participant's completion of the study 168 days after their Day 1 study vaccination. Specifically, solicited AEs will be collected for 7 days (day of study vaccination and 6 following days) after each dose of study vaccine. If a solicited AE started during the 7 days post-vaccination and continues beyond the 7 days, it will continue to be reported as a solicited AE. Unsolicited AEs will be collected for 28 days (day of study vaccination and 27 following days) after each dose of study vaccine. SAEs will be collected from Day 1 study vaccination through the end of the study (Day 169 Telephone visit).

Any untoward medical occurrence after signing the ICF but before receipt of study vaccine, although not to be reported as an AE, if it is assessed as related to participation in the study, must still be reported by email to PATH within 3 days of awareness.

Due to uncertainties around the COVID-19 pandemic and the potential for stay-at-home orders, if a subject is unable to come to the clinic for a visit, the scheduled and applicable safety assessments may be conducted by telephone.

8.3 Severity of Adverse Events

Solicited AEs:

The severity of all solicited AEs will be graded based on the grading scale in Table 4 below.

Table 4: Grading scale to grade the severity of solicited AEs

Solicited AE	Grade	Definition
Fever (oral body temperature)	0	< 100.4°F (none)
	1	≥ 100.4°F to < 101.5°F
	2	≥ 101.5°F to < 102.7°F
	3	≥ 102.7°F

Diarrhea	0	None
	1	2 to 3 loose stools per 24 hours
	2	4 to 5 stools per 24 hours
	3	6 or more watery stools per 24 hours
Chills or fatigue or headache or myalgias or arthralgias or nausea or vomiting or abdominal pain	0	None
	1	Causes no or minimal interference with usual social & functional activities
	2	Causes greater than minimal interference with but does not prevent usual social & functional activities
	3	Causes inability to perform usual social & functional activities

Abnormal clinical safety laboratory test results reported as AEs:

The severity of abnormal clinical safety laboratory test results reported as AEs will be graded based on the grading scale in Table 5 below. The table is adapted from the FDA Guidance for Industry: Toxicity Grading Scale for Healthy Adult and Adolescent Volunteers Enrolled in Preventive Vaccine Clinical Trials (September 2007). In the absence of a diagnosis, any clinical safety laboratory test result that meets the definition of an SAE as determined by the investigator (e.g., life-threatening) must be reported as an SAE.

Table 5: Grading scale to grade the severity of abnormal clinical safety laboratory test results reported as AEs

Parameter	Grade 1 (Mild)	Grade 2 (Moderate)	Grade 3 (Severe)
Hemoglobin [Female] (g/dL)	11.0 – 12.0	9.5 – 10.9	< 9.5
Hemoglobin [Female] change from baseline value (g/dL)	Any decrease – 1.5	1.6 – 2.0	> 2.0
Hemoglobin [Male] (g/dL)	12.5 – 13.5	10.5 – 12.4	< 10.5
Hemoglobin [Male] change from baseline value (g/dL)	Any decrease – 1.5	1.6 – 2.0	> 2.0
WBC Increase (cell/mm^3)	10,800 – 15,000	15,001 – 20,000	> 20,000
WBC Decrease (cell/mm^3)	2,500 – 3,500	1,500 – 2,499	< 1,500
Lymphocytes Decrease (cell/mm^3)	750 – 1,000	500 – 749	< 500
Neutrophils Decrease (cell/mm^3)	1,500 – 2,000	1,000 – 1,499	< 1,000
Eosinophils (cell/mm^3)	650 – 1,500	1,501 – 5,000	> 5,000
Platelets Decreased (cell/mm^3)	125,000 – 140,000	100,000 – 124,000	< 100,000
Creatinine (mg/dL)	1.5 – 1.7	1.8 – 2.0	> 2.0
ALT increase by factor	1.1 – 2.5 x ULN*	> 2.5 – 5.0 x ULN*	> 5.0 x ULN*
Total bilirubin increase by factor	1.1 – 1.5 x ULN*	> 1.5 – 2.0 x ULN*	> 2.0 x ULN*

*ULN = upper limit of normal

Other AEs:

The severity of all AEs other than solicited AEs and abnormal clinical safety laboratory test results reported as AEs will be assessed by the investigator and participant (as applicable) based on the Division of AIDS (DAIDS) Table for Grading the Severity of Adult and Pediatric Adverse Events, corrected version 2.1, July 2017, of the US National Institute of Health, available from:

https://rsc.niaid.nih.gov/sites/default/files/daidsgradingcorrectedv21.pdf

The severity grading criteria provided grade AEs from mild (grade 1) to life threatening (grade 4). All AEs leading to death are Grade 5 events. AEs are graded with the worst severity grade during the illness/symptoms. Life threatening events and events leading to death must be reported as SAEs.

8.4 Causality of Adverse Event

The study investigators will determine the causal relationship between the study vaccine and the AE. The causality assessment is made based on the available information at the time of reporting and can be subsequently changed according to follow-up information. Assessment of causality is based on clinical judgment and should take into consideration the following factors:

- Is there a temporal relationship between the event and administration of the study vaccine?
- Is there a plausible biological mechanism for the study vaccine to cause the AE?
- Is there a possible alternative etiology for the AE such as concurrent illness, concomitant medications?
- Are there previous reports of similar AEs associated with the study vaccine or other vaccines in the same class?

For this study, the investigator must classify the causality of the AE according to the categories defined below:

Related: There is a reasonable possibility that the study vaccine caused the event. "Reasonable possibility" means that there is evidence to suggest a causal relationship between the study vaccine and the AE.

Not Related: There is not a reasonable possibility that the administration of the study vaccine caused the event.

8.5 Follow-up of Adverse Event

All reported AEs should be followed until resolution or stabilization, or until the participant's participation in the study ends. The investigator must ensure that any AEs that are ongoing at study completion have been appropriately referred to the local health care system for continuation of care. Participants who have an ongoing study vaccine related SAE at study completion or at discontinuation from the study will be followed by the investigator until the event is resolved or determined to be irreversible, chronic, or stable by the investigator.

The outcome of adverse events will be assessed at the time of last observation as per the following categories:

- Recovered/resolved without sequelae
- Recovered/resolved with sequelae
- Recovering/resolving
- Not recovered/not resolved
- Fatal
- Unknown. The outcome of the AE is not known

Due to uncertainties around the COVID-19 pandemic, if a subject has a reportable adverse event that meets the clinical criteria of the current CDC case definition of COVID-19, the investigator will ensure that the subject will undergo standard of care diagnostic testing and if needed, a quarantine period for SARS-CoV-2.

8.6 General Guidance on Recording Adverse Events

To improve the quality and precision of acquired AE data, the investigator should observe the following guidelines:

- AEs must be assessed for severity (graded) and causality and reviewed by the site investigator.
- If an AE (e.g., low hemoglobin) meets the criteria for two different severity grades simultaneously (e.g., Grade 1 for absolute hemoglobin level and Grade 2 for change from baseline value), the severity grade reported should be the most severe grade (i.e., Grade 2 in the example provided).
- Whenever possible, use recognized medical terms when recording AEs on the AE CRF. Do not use colloquialisms or abbreviations.
- If known, record the diagnosis (i.e., disease or syndrome) rather than component signs, symptoms and laboratory values (e.g., record congestive heart failure rather than dyspnea, rales, and cyanosis); however signs and symptoms that are considered unrelated to an encountered syndrome or disease should be recorded as individual AEs (e.g., if congestive heart failure and severe headache are observed at the same time, each event should be recorded as an individual AE).
- AEs occurring secondary to other events (e.g., sequelae) should be identified by the primary cause. A "primary" AE, if clearly identifiable, generally represents the most accurate clinical term to record. If a primary serious AE (SAE) is recorded, events occurring secondary to the primary event should be described in the narrative description of the case. For example:

  Orthostatic hypotension ® Fainting and fall to floor ® Head trauma® Neck pain

  The primary AE is orthostatic hypotension.

- Death is an outcome of an event. The event that resulted in the death should be recorded and reported on the SAE CRF.
- For hospitalizations for surgical or diagnostic procedures, the illness leading to the surgical or diagnostic procedure should be recorded as the SAE, not the procedure itself. The procedure should be captured in the case narrative as part of the action taken in response to the illness.
- Pregnancies that occur in study participants are not considered AEs and will be recorded on a separate Pregnancy CRF. Pregnancy outcomes that include spontaneous abortion, stillbirth or any congenital anomaly must be reported as SAEs.

8.7 Reporting of SAEs

PATH has designated Emmes with authority to coordinate SAE reporting activities. All SAEs which occur during the study, whether considered to be associated with the study vaccine or not, must be reported within 24 hours of the site becoming aware of the event to the Emmes Medical Monitor by one of the mechanisms provided (e.g., electronic data capture [EDC] system, email, or fax). Contact details and instructions for submitting SAEs will be provided in a handout located in the Investigator Site File. If the SAE is fatal or life-threatening, the study medical monitor shall be informed immediately by email.

The investigator should not wait for additional information to fully document the event before notifying Emmes. When additional information becomes available, follow-up submissions will be submitted. The initial SAE form should be completed with all information known at the time and should include minimal elements for initial assessment:

- Name and contact of the investigator submitting the SAE report
- Participant ID number
- Date participant received study vaccine

- Description of the SAE and date of event onset
- Investigator's preliminary assessment of severity and causality

When applicable, hospital case records and autopsy reports should be obtained (without name or personal identifiers).

The investigator is also responsible for reporting all SAEs to their IRB/IEC in accordance with institutional policy and adequate documentation of this reporting must be provided to Emmes.

Reporting procedures for all SAEs will be followed as per FDA regulatory guidelines. The study Safety Management Plan (SMP) will contain all the details of safety and regulatory reporting. Emmes is responsible for safety reporting to the FDA within the following time periods:

- Serious and unexpected suspected adverse reaction within 15 days of awareness
- Fatal or life-threatening suspected adverse reaction within 7 days of awareness

8.8 Protocol Deviations

A protocol deviation is any noncompliance with the clinical trial protocol, GCP, or site SOP requirements. The noncompliance may be either on the part of the participant, the PI, or the study site staff. As per ICH E6(R2), the investigator must not deviate from the protocol unless it is required to eliminate an immediate hazard to a trial participant. If required, as soon as is possible, the implemented deviation and the reason for it should be submitted to the IRB/IEC and PATH and a determination made as to whether a protocol amendment is required.

Deviations can be categorized as either major or minor. Reporting requirements of deviations depend on the type or degree of deviation. The procedures for handling protocol deviations will occur as follows: The PI will be notified by study staff as soon as possible if he or she is not present when the deviation is discovered.

The timeline for reporting protocol deviations to the IRB and PATH is determined by the categorization of the deviation as major or minor. Major protocol deviations are a subset of protocol deviations that may significantly impact the completeness, accuracy, or reliability of the study data or that may significantly affect a participant's rights, safety, or well-being. For example, major protocol deviations may include failure to obtain informed consent, failure to report SAEs, enrolling participants in violation of key eligibility criteria designed to ensure a specific participant population or failing to collect data necessary to interpret primary endpoints, as this may compromise the scientific value of the trial. Major deviations that occur in this protocol will be reported to PATH in an expedited manner (within 48 hours) and to the IRB/IEC as per their stated requirements.

Minor deviations are departures from the protocol that do not involve participant safety or integrity of the study data. Minor deviations will be reported to the IRB/IEC in the context of the annual Continuing Review Report or as per their stated requirements if any different. Major deviations should also be summarized in the continuing review reports.

Knowledge of any instances of serious or continuing non-compliance with the regulations or requirements will be reported immediately to PATH and the IRB/IEC.

9 SAFETY OVERSIGHT

The site PIs and designated site staff will be responsible for continuous close safety monitoring of all study participants and for alerting PATH if unexpected concerns arise or once aware that any study pause criteria are met.

9.1 Routine Reviews by Protocol Safety Review Team (PSRT)

The Protocol Safety Review Team (PSRT), composed of the site PIs, the PATH Medical Officer and Emmes medical monitor, will routinely monitor safety throughout the duration of the trial. The PSRT will be chaired by the PATH Medical Officer and may seek additional independent expert medical opinion as dictated by needs, including referral to the IDMC, particularly for consideration of unblinded review. The Emmes statistician, with assistance of the data management staff, will prepare blinded safety reports for review by the PSRT. These reports will provide at a minimum the following information:

- accrual and participant status data regarding completion of study vaccinations and study visits
- summaries of solicited, unsolicited, and serious adverse events
- predefined abnormal safety laboratory test results
- updated medical history and concomitant medication listings

The PSRT safety review will be conducted by teleconference (or electronically when appropriate) occurring at least once monthly until all participants have completed their Day 57 study visits and as needed thereafter for the remainder of the study. In addition to safety review, the PSRT may elect to discuss trial conduct issues that impact study integrity and participant safety. These may include, but are not limited to, data quality, critical monitoring findings, study vaccine concerns, and issues with research specimens. Emmes will also notify the PSRT of the need for ad hoc safety reviews whenever it is aware of a SUSAR or adverse events that meet pre-specified study pause criteria as per section 9.3.

9.2 Independent Data Monitoring Committee (IDMC) Reviews

An IDMC composed of at least three independent members with expertise in vaccine clinical trials will be convened by PATH to periodically review the conduct and safety of the study. The responsibilities and procedures of the IDMC are defined in the IDMC Charter.

The IDMC will convene for an organizational meeting prior to study initiation and then for at least three scheduled meetings during the conduct of the study. For each of the two nOPV types, one meeting will be scheduled once 15 participants have completed their one week post-initial dose study vaccination visits. The third meeting will be scheduled based upon observed enrollment rates and study progress. In addition to these routinely scheduled meetings, if the PSRT has serious safety concerns or study pause criteria are met, the IDMC will convene by teleconference to jointly review the data. The IDMC reviews will be summarized with recommendations to PATH as to whether there are safety concerns and whether the study should continue without change, be modified, or be terminated.

If, at any time, a decision is made to permanently discontinue administration of study vaccines in all participants, PATH will notify the FDA and the site investigators of record will notify the responsible IRB/IEC expeditiously.

9.3 Study Pause Rules

The following study pause rules will automatically halt any further study vaccinations; however, participants already vaccinated will continue to be followed for safety during the pause. These pause rules

refer to suspected adverse reactions and will be triggered automatically if any of the criteria described below are met in a specific study group during the conduct of the study:

- One or more participants experience an SAE that cannot reasonably be attributed to a cause other than study vaccine
- Three or more participants experience the same severe (grade 3) solicited AE within 7 days following study vaccination that cannot reasonably be attributed to a cause other than study vaccine
- Three or more participants experience the same severe (grade 3) unsolicited AE (including laboratory abnormality) within 28 days following study vaccination that cannot reasonably be attributed to a cause other than study vaccine

9.4 Study Pause Procedure

The PSRT will be notified immediately by Emmes if they ascertain that a pause rule has been met. If a site investigator or the PSRT first become aware that a pause rule has been met, they will inform Emmes immediately. Emmes will cease randomization and notify the site PIs that a pause rule has been met and that no further enrollment should occur, and no study vaccines are to be administered until specific notification is provided that study vaccinations can resume.

As soon as it is confirmed that a study pause rule has been met, the IDMC will be notified and will expeditiously (within 48 hours, if possible) convene by teleconference to review all available, relevant information, including initial exclusive access to unblinded data. The IDMC reviews will be summarized with recommendations to PATH as to whether there are safety concerns and whether the study should continue without change, be modified, or be stopped.

If at any time, a decision is made to permanently discontinue administration of study vaccine in all participants, notification will be provided by PATH to the NRA and by the PI to the IRB/IEC within 48 hours.

If PATH re-starts the study after IDMC review and recommendation, enrollment and study vaccination may resume. The PIs will report the study pause and decision to resume to their IRB/IEC.

10 DATA HANDLING and RECORDKEEPING

The investigator is responsible for assuring that the data collected are complete, legible, attributable, accurate, and recorded in a timely manner. Data collection is the responsibility of the site clinical trial staff under the supervision of the site PI. All source documents and laboratory reports must be reviewed by the clinical team and data entry staff, who will ensure that they are accurate and complete.

Emmes is responsible, under oversight of PATH, for data management activities, including quality review, analysis, and reporting of the study data according to Standard Operating Procedures (SOPs).

10.1 Definitions

10.1.1 Source Data

All information in original records and certified copies of original records or clinical findings, observations, or other activities in a clinical trial necessary for the reconstruction and evaluation of the trial. Source data are contained in source documents (original records or certified copies; ICH E6[R2] section 1.51).

10.1.2 Source Documents

Original documents, data and records (e.g., hospital records, clinical and office charts, laboratory notes, memoranda, participants' diaries of evaluation checklists, pharmacy dispensing records, recorded data from automated instruments, copies or transcriptions certified after verification as being accurate and complete, microfiches, photographic negatives, microfilm or magnetic media, x-rays, participant files, and records kept at the pharmacy, at the laboratories, and at medico-technical departments involved in the clinical trial (ICH E6[R2] section 1.52).

10.2 Data Capture Methods (Case Report Form Development and Completion)

The clinical data in source documents will be entered directly into a 21 CFR Part 11-compliant EDC system by trained and qualified study staff. The electronic CRF (eCRF) for the EDC system will be developed by the Emmes data management center with input from study staff and approval of PATH. Clinical data for each participant will be entered directly into the eCRF from the source documents.

It is the site PIs' responsibility to ensure the accuracy, completeness, and timeliness of the data reported in the participant's eCRF and any supporting documentation. All source documents should be completed in a neat, legible manner to ensure accurate interpretation of data. Source documentation supporting the eCRF data should document the dates and details of study procedures, AEs and participant status. The site PIs/institutions will maintain all information in the eCRFs and all source documents that support the data collected from each participant in a secure area and treated as confidential material.

10.3 Data Management

A thorough Data Management Plan and corresponding database compliant with ICH requirements will be developed by Emmes Data Management (DM). Emmes DM will build, validate, and maintain a Good Clinical Practice (GCP) compliant EDC system. The data system will include password protection and internal quality checks, such as automatic range checks, to identify data that appear inconsistent, incomplete, or inaccurate. Write access to the system will be limited to authorized Investigators and study staff, and the system will automatically keep an audit trail of all entries and corrections in the eCRF.

Emmes DM will perform all activities as per their SOPs, and in accordance with PATH SOPs. Coding of medical history and adverse events will be performed using the latest version of MedDRA. Medications will be coded using the latest World Health Organization Drug Dictionary. The eCRFs and any supporting documentation should be available for retrieval or review at any given time.

10.4 Retention of Study Records

The site PIs are responsible for retaining study records for a period of 2 years following the date that a marketing application is approved for the product or, if no application is to be filed or, if a file application is not approved, until 2 years after the investigation is discontinued and the NRA is notified. PATH will be responsible for providing the site with date of vaccine approval or IND/regulatory withdrawal. No records will be destroyed without the written consent of PATH. PATH will notify the investigator in writing when the trial related records are no longer needed.

These records are also to be maintained in compliance with local IRB/IEC and local authority medical records retention requirements, whichever is longest. Storage of all trial-related documents will be such that confidentiality will be strictly maintained to the extent provided by local law.

11 STATISTICAL CONSIDERATIONS

This section summarizes the primary features of the statistical analysis for the study. A statistical analysis plan (SAP) will be prepared and finalized prior to database lock and will specify all analyses to be performed. Emmes will author the SAP and produce the statistical analysis, under the guidance and oversight of PATH.

11.1 Overview and General Design

This multicenter trial is the first-in-human assessment of these novel oral poliomyelitis vaccines. It will be an 8-arm, randomized, observer-blind, controlled trial, with Sabin monovalent vaccines serving as the control for each novel type (see study schema in Section 3). Within each cohort and according to prior vaccination status (exclusively IPV or OPV-containing), healthy, adult participants will be randomized into the study arms receiving mOPV1 or nOPV1 (cohorts 1 and 2, 115 participants), and once each of those cohorts are completely accrued, participants will be randomized into the corresponding type-specific cohorts receiving mOPV3 or nOPV3 (cohorts 3 and 4, 115 participants) in the same manner.

11.2 Randomization Procedures

Within cohorts 1 and 3, participants with exclusively IPV prior vaccination will be randomized 1:1 to nOPV vs mOPV with all participants receiving a single dose, and within cohorts 2 and 4, previously OPV-vaccinated participants will be randomized 2:1 to nOPV vs mOPV, with all participants receiving 2 doses, with the second dose 28 days following the first dose. Block randomization will be stratified by site to ensure balance within each site, without a prespecified number to be enrolled at each site. The randomization scheme will be generated and maintained by Emmes. Participants will be randomized using the Emmes IWRS. Any enrolled participant who is randomized but withdraws for any reason prior to vaccination will be replaced.

The site pharmacists with primary responsibility for dispensing study vaccines are charged with maintaining security of the treatment assignments.

11.3 Sample Size

The sample sizes chosen for this study are chosen primarily to enable evaluation of safety in a sufficient number of adults prior to a subsequent study phase in younger individuals, to provide an opportunity to demonstrate an immune response with study vaccination, and to enable collection of stool samples to permit a preliminary evaluation of genetic stability of shed virus. Definitive evaluations of immunogenicity, viral shedding, and genetic stability are planned for future study phases.

Primary Safety Evaluation

For the safety endpoints, the table below describes the probability of detection of rare events and the precision afforded by the sample sizes used in this study. These levels are consistent with standard phase 1 vaccine studies aimed at safety evaluation and provide adequate ability to detect unexpected adverse events prior to age de-escalation in a subsequent study.

Sample size	Minimum AE rate producing 95% probability of observation with given sample size (%)	95% Confidence interval for event rate if 0 events are observed	95% Confidence interval for event rate if 1 event is observed
**15**	18.2	(0.0, 21.8)	(0.1, 31.9)

**20**	13.9	(0.0, 16.8)	(0.1, 24.9)
**25**	11.3	(0.0, 13.7)	(0.1, 20.4)
**50**	5.8	(0.0, 7.1)	(0.1, 10.6)
**70**	4.2	(0.0, 5.1)	(0.04, 7.7)

Minimum adverse event rates required to have ≥95% probability of detecting at least one such event with given sample sizes, along with two-sided 95% exact confidence intervals if 0 or 1 events are observed.

Immunogenicity Evaluation

Assessment of immunogenicity is a secondary objective in this study. Due to the prior vaccinations received by those to be enrolled into the study, baseline immunity (titer of type-specific neutralizing antibody) to poliovirus types 1 and 3 is expected to be high, with the level boosted by study vaccination. While it is expected that the presence of an immune response can be observed by an increase in neutralizing antibody titer following vaccination, and because the post-vaccination immunity is anticipated to exceed the assay upper limit of quantification (ULOQ) for some participants, definitive comparisons of study vaccination groups to the relevant control (nOPV vs type-specific control, within cohort according to prior vaccination [exclusively IPV vs OPV-containing]) will not be possible within this phase 1 study. Evaluation of immunogenicity, therefore, will be primarily descriptive in nature; comparisons to the relevant control will be made, but the sample size is not based on achieving a specific level of statistical power for these comparisons. Comparative evaluation for immunogenicity will focus on the cohorts previously receiving OPV vaccinations but will also be conducted among exclusively IPV-vaccinated participants.

Prior studies of type 2 in OPV-vaccinated adult populations indicate baseline immunity within the quantifiable range, with mean $\log_2$ neutralizing antibody (NAb) titers ranging from 7.8 to 8.8 (unpublished data), and with the majority of participants achieving NAb titer >ULOQ 28 days following a single dose of a Sabin-2 vaccine as well as investigational type 2 vaccines. Using a regression model permitting censoring at assay lower limit of quantification (LLOQ) and ULOQ, the standard deviation (SD) of post-vaccination NAb titers is estimated to be in the range of 3 – 3.5 on the $\log_2$ scale, which is consistent with other published data for type 1 (prior to a boost vaccination in adulthood) [11]. In the study involving type 2, the post-vaccination GMT ratio (candidate/control) and its corresponding CI were able to be estimated; similar methods will be employed here (see below). With respect to binary immune response endpoints (seroconversion, other seroresponse rates), data from the type 2 study indicate seroconversion rates ranging from approximately 30% to 75%, depending on the vaccine and potency level administered. Rates of a minimum 2-fold response ranged from approximately 50% to 75%, with rates of any fold-rise ranging from approximately 60% to 85%.

Using these values, simulation studies were conducted to evaluate the precision of estimates of the GMT, GMT ratio, response rates, and differences in response rates, assuming that 48 and 24 of the 50 and 25 or 28 and 14 of the 30 and 15 prior OPV participants randomized to receive nOPV or mOPV, respectively, provide samples for analysis. The figure below displays the average half-width of the two-sided 95% confidence intervals for the four evaluations across the range of relevant inputs ($\log_2$ NAb SD for GMTs, true rate for the various response rates). The level of precision expected to be available, described within the figures, is considered adequate to determine if there is sufficient evidence of an immune response to warrant progressing into populations where the immune response may be more thoroughly described and compared.

The figure indicates, for example, that if the SD of the log_2 post-baseline serum NAbs is 3.0 and both candidate and control vaccines are equally immunogenic, then one can expect, on average, that the CI for the difference in mean log_2 NAbs will be approximately (-1.48, 1.48) with 48:24 or (-1.92, 1.92) with 28:14 when the estimated difference is 0 (ratio = 1), providing high confidence that the actual absolute log_2 difference is <2 (ratio <4). If instead the SD is 2.0, the CI would be approximately (-0.97, 0.97) with 48:24, providing confidence that the actual absolute difference is <1 (ratio <2). For the lower sample size, with an SD of 2.0, the CI would be approximately (-1.25, 1.25), providing high confidence that the actual absolute log_2 difference is <1.58 (ratio < 3). For the candidate vaccine group alone, assuming a SD just less than 3.5 and 48 evaluable subjects, the CI for the mean log_2 titer would be approximately ±1.0 log_2. That is, if the estimated GMT is 2^8 (256), the figure indicates the CI would be expected to be approximately (2^7, 2^9), or equivalently (128, 512), on average. If instead the SD is 2.5 with 48 evaluable subjects, the CI would be expected to be approximately ($2^{7.3}$, $2^{8.7}$), or equivalently (158, 416), on average.

With respect to response rates, for which precision is naturally lower than the continuous measurement of serum NAbs: if a true response rate is 50% in each group (maximum variability), then the CI for the estimated candidate vaccine response rate would be expected to average ±15% (±20% for 28 evaluable subjects) from the point estimate, and the CI for the difference in response rates would be expected to average ±23% (±30% for 28:14) from the point estimate.

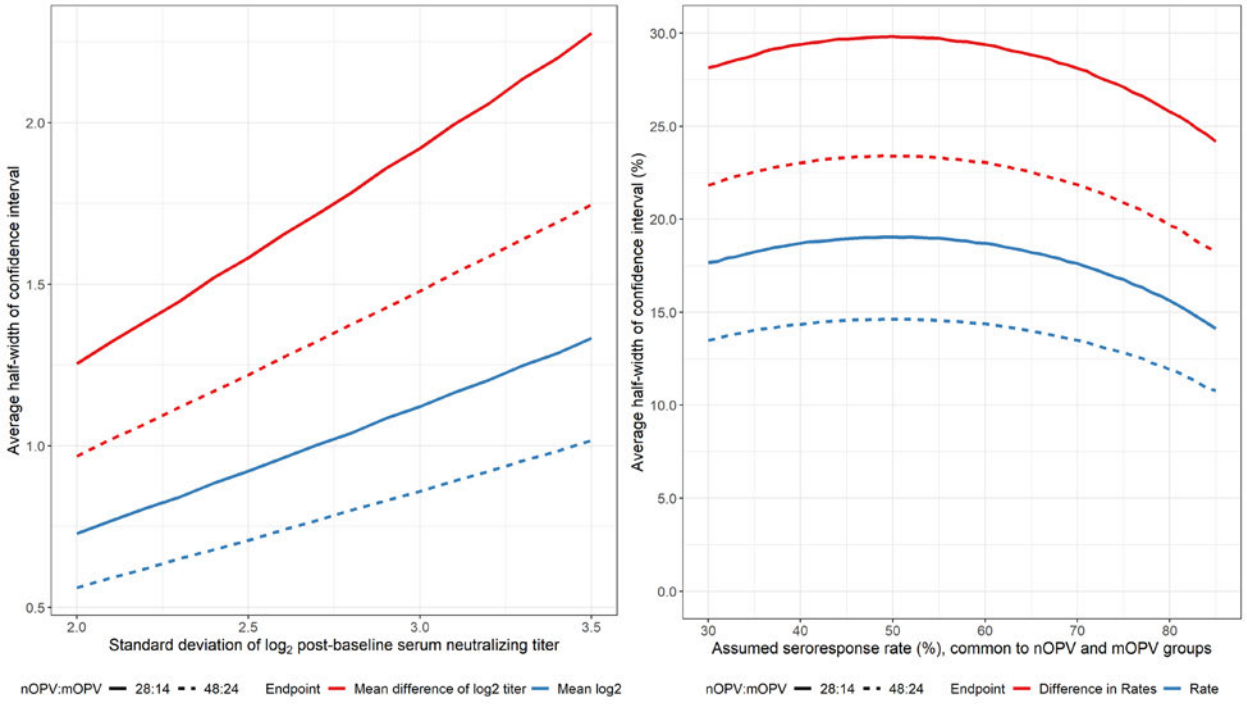

Intestinal Immunity, Genetic Stability

In recognition that previously OPV-vaccinated participants are unlikely to shed vaccine virus in substantial quantities, the 30-40 participants for each type with exclusive prior IPV vaccination to be randomized 1:1 to nOPV or mOPV are included primarily to provide a preliminary indication of intestinal take (shedding of virus, indicating replicating infection), as well as to provide samples for potential genetic stability analysis of shed virus, including NGS and evaluation in a mouse neurovirulence assay. The relevant

neurovirulence assay for comparison to Sabin-strain vaccines is yet to be developed, but it is anticipated that a null hypothesis of no difference, vs. an alternative hypothesis of superiority of nOPV to mOPV will be evaluated from this model; a defined level of statistical power is not a requirement for this preliminary evaluation. It is currently unknown what levels of paralysis may be seen in transgenic mice in such an assay as assay development is ongoing but based on preclinical data indicating no paralysis associated with passaged candidate vaccine virus, and the known reversion to neurovirulence resulting in frequent paralysis of mice inoculated with reverted Sabin-strain vaccines, sufficient discriminatory capability is expected to be provided by 15-20 participants per vaccine.

11.4 Definitions of Populations to be Analyzed

11.4.1 Enrolled Population

The Enrolled Population is defined as all participants who provide informed consent, regardless of the participant's randomization and treatment status in the study.

11.4.2 Safety Population

The Safety Population is defined as all participants in the Enrolled Population who received a study vaccine.

11.4.3 Full Analysis Population

The Full Analysis Population (FAP) is defined as all participants in the Enrolled Population who were randomized, received a study vaccination, and provided a baseline and at least one post-study vaccination evaluable serum sample.

11.4.4 Per-Protocol Population

The Per-Protocol (PP) population is defined as all participants in the FAP who correctly received all study vaccinations per randomization with no major protocol deviations that are determined to potentially interfere with the immunogenicity result of the participant. The PP population will be defined on the time-point level; that is, a participant may be evaluable in the PP population with their baseline and Day 29 samples, but not for the Day 57 sample due to a major protocol deviation. For summaries of the PP population on the participant level, the PP membership for the Day 29 visit will be used.

Prior to database lock, the database will be searched for potentially disqualifying deviations. Additionally, protocol deviations will be collected from monitoring and medication listings will be reviewed; this information will be reviewed in a Data Review Meeting (DRM) attended by representatives from PATH, Emmes, and the study sites to determine PP population eligibility. Efforts will be taken to blind participant group membership during this review to the extent possible in this observer-blind study. The following constitute potential criteria for elimination from the per-protocol population. The list is not exhaustive, as unexpected deviations may arise requiring unique consideration.

- Missed vaccinations
- Significant non-compliance with visit windows
- Any eligibility criteria not met
- Receipt of a non-study vaccine
- Receipt of immunosuppressants or immune modulators
- Receipt of study vaccine not stored as per manufacturers approved storage condition
- Incomplete vaccine dose administration or vomiting within 5 minutes of vaccination

- Serological results unavailability
- Wrong randomization
- Dosed with wrong treatment arm

A DRM report will provide the criteria used for determination, as well as list the participants excluded and accompanying rationale.

11.4.5 Other Populations

The SAP will define additional subgroup participant populations for specific analyses.

11.5 Analytical Methodology

11.5.1 Descriptive Methodology

All data collected will be summarized and/or listed. Analyses will be performed using SAS® software (SAS Institute Inc., Cary, NC, USA) version 9.4 or higher.

Unless otherwise specified, descriptive statistics include the mean, SD, median, minimum, maximum for continuous variables, and the number and proportion in each group for categorical variables. Unless otherwise specified here or in the SAP, statistical tests, and confidence intervals (CIs) will be computed using a two-sided 5% significance level. Exact (Clopper-Pearson) CIs will be used for univariate summaries of dichotomous variables, and Miettinen-Nurminen score-based confidence intervals will be used for rate differences. All proportions will use as denominator the number of participants contributing data at the specified time point within the specified group and study population.

Summaries will be presented by group and by time point, where relevant.

For all summaries outlined below, the SAP will contain additional detailed description of the analyses to be conducted.

11.5.2 Analysis Sequence

Analysis of immunological endpoints will occur separately for each type, in advance of the completion of long-term safety follow-up of participants. Following database lock for long-term safety follow-up and completion of stool viral shedding assays, a complete final analysis will be produced and incorporated into the clinical study report (CSR).

11.5.3 Changes in Analysis Plan

Any deviations or changes from the statistical analyses specified in the protocol will be described and justified in the SAP and the CSR.

11.5.4 Baseline and Demographic Characteristics, and Participant Disposition

Descriptive statistics will be computed for demographic (e.g., height, weight, race, sex) characteristics in both the safety and PP populations, and other initial participant characteristics (e.g., medical and surgical history, concomitant diseases) in the safety population.

Concomitant medications will be coded using the WHO Drug Dictionary. Medical history will be coded using the most recent version of the Medical Dictionary for Regulatory Activities (MedDRA).

Participant disposition including dropout and reasons for dropout, as well as study population membership will be summarized descriptively, and supported with a CONSORT (Consolidated Standards of Reporting Trials) diagram.

Screening serology will be summarized descriptively, including HIV, HBV, and HCV, pregnancy testing, and type-specific anti-polio antibodies among all participants, and in the enrolled population.

11.5.5 Safety Analysis

All safety analyses will be conducted in the safety population. Summaries will be computed separately by group, and within type across group (i.e., combined Groups 1 and 3 compared to combined Groups 2 and 4, and combined Groups 5 and 7 compared to combined Groups 6 and 8).

The extent of exposure will be summarized by tabulating the number of doses of a given vaccine received for each participant, as well as descriptive summaries of the time (days) between vaccinations

11.5.5.1 Solicited Adverse Events

Solicited events will be summarized by computing the proportion of participants with observation of any event, and any event according to event type, over all vaccinations within groups, and for each vaccination individually within group. Additional analyses will summarize the frequency and duration of events ongoing at Day 7 post-study vaccine administration. Summaries will be prepared corresponding to maximum severity and duration per participant, where relevant. Solicited adverse event rates will be accompanied by two-sided exact 95% confidence intervals. Within cohorts (separately for each type), the rate of solicited adverse events will be compared using a two-sided Fisher's exact test for pairwise comparisons, both overall and by type following each dose, and overall (across doses); this will be repeated for severe and for moderate or greater events.

11.5.5.2 Unsolicited Adverse Events

All unsolicited adverse events will be listed and summarized in tables. Unless an AE is classified as an SAE, summaries of unsolicited AEs will be made using only those events recorded with onset within 28 days of the prior vaccine dose. Unsolicited adverse events will primarily be summarized on the participant level, where a participant contributes once to a given event type under the maximum severity and/or causality, as appropriate. Tables will additionally display the number of events of a given type observed within a group, regardless of the number of participants from which they originate.

Unsolicited adverse events will be summarized by severity and by relationship; SAEs will be summarized overall, and by reason for seriousness designation. In addition, all AEs, coded with MedDRA, will be summarized by System Organ Class (SOC) and Preferred Term (PT), and separate tables by SOC and PT will be prepared for severe unsolicited AEs, severe AEs, related AEs, severe related AEs, and SAEs. A table will be prepared summarizing all preferred terms occurring in ≥2% of participants. Additional summaries will include the rate of participants experiencing an AE leading to withdrawal from vaccinations, or from the study. Additional listings will be prepared for severe AEs, related AEs, and SAEs. Within OPV type, the rates of participants experiencing severe adverse events, related adverse events, and serious adverse events will be compared across groups using a two-sided Fisher's exact test for pairwise comparisons.

11.5.5.3 Safety Labs

Each continuous hematology and chemistry laboratory test will be evaluated by means of descriptive statistics (i.e., number of participants, mean, SD, median, minimum, and maximum) on the actual values,

at each assessment time point and by group. Changes from baseline will also be summarized by assessment time point and by group.

Clinical safety laboratory test values will be evaluated according to the table for grading the severity of adverse events provided in this protocol or in accordance with the normal ranges of the clinical laboratory (below, within, or above normal range) for parameters for which no toxicity grades are defined.

Clinical laboratory abnormalities will be summarized by group, parameter, time point, and grade, including the maximum grade post-baseline abnormality for each participant for each parameter. Summaries will include any baseline abnormality across parameters within group, and any post-baseline abnormality across visits within parameters, as well as across parameters and visits. Abnormalities will also be summarized in a shift table, displaying the frequency of post-baseline abnormalities by grade (with separate categories for high and for low values of a given lab, where relevant) cross-tabulated with the baseline classification for each time point for each lab. Boxplots will display the raw value by lab parameter and study collection day and will include separate symbols to depict values out of range, and those that are grade 2 or higher.

11.5.5.4 Other Safety Measures

Vital signs will be summarized descriptively, including change from baseline for continuous measures. Vital sign abnormalities will be summarized descriptively.

Abnormal findings in physical examination will be summarized descriptively.

11.5.6 Immunogenicity Analysis

All immunogenicity analyses will be performed in the PP population and select analyses will be repeated in the FAP. All descriptive analyses will be conducted across all participants within a group/time point combination, and these will be repeated for each site.

11.5.6.1 Descriptive Analyses

At each time point where NAb titers are obtained:

- Median of $\log_2$ antibody titers will be computed along with 95% CIs
- GMT with accompanying 95% CIs will be computed. GMTs will be estimated with likelihood-based methods (SAS PROC LIFEREG) to accommodate censoring at assay ULOQ and LLOQ.
- The geometric mean fold rise (GMFR) will be computed for each post-baseline time point as the reverse-transformed difference between the post-baseline $\log_2$ value and the baseline $\log_2$ value and accompanied by two-sided paired-sample 95% CIs computed using asymptotic methods on the $\log_2$ scale, then reverse-transformed. A subgroup analysis of GMFR will consider only those participants with values between the LLOQ and ULOQ (exclusive of the endpoints) for the numerator and denominator samples. For post-dose-2 GMFR, this will be computed from baseline, and from the pre-dose-2 titer. Among these participants, a plot of the $\log_2$ fold rise vs the baseline $\log_2$ antibody titer will be generated.
- Type-specific seroconversion rates with 95% exact CIs will be tabulated for post-vaccination time points. Seroconversion rate will be computed among those participants with seroconversion possible to observe (within 4-fold of the assay ULOQ). For post-dose-2 seroconversion, seroconversion rates will be computed both from baseline and from the pre-dose-2 titer

- In addition, the rate of any fold-rise (titer increased from baseline) and a minimum 2-fold rise in titer and accompanying CIs will be computed in the same manner

- Type-specific seroprotection rates with 95% exact CIs will be tabulated
- Plots of the reverse cumulative distribution of antibody titers will be generated

Additional figures will display immunity and changes in immunity over time, by type. Additional subgroup computations will be specified and conducted, per the SAP.

11.5.6.2 Comparative Analyses

No specific immunogenicity hypothesis is to be tested. Descriptive comparative evaluation will be conducted with multiple endpoints; such evaluations will be conducted for the nOPV-vaccinated groups compared to the mOPV-vaccinated control groups within type, and within background vaccination cohort (within groups defined by exclusive IPV prior vaccination, and separately among those previously receiving OPV). Separately for each pairwise comparison, the NAb GMT ratio (nOPV/mOPV) will be estimated via a linear model of the $\log_2$ NAb titer as a function of group with a fixed parameter for site and a covariate for the $\log_2$ NAb titer level. This will be conducted using SAS PROC LIFEREG to account for censoring of the dependent variable assuming a Normal error distribution on the $\log_2$ scale; baseline values achieving ULOQ or LLOQ will be replaced with ULOQ or LLOQ, as appropriate, for the covariate. The log-scale difference and its corresponding confidence interval, estimated with SAS LSMEANS, will be reverse-transformed to obtain the estimated GMT ratio and corresponding 95% confidence interval. Among the subset of participants where baseline and post-baseline NAb titers are below the assay ULOQ, the fold-rise in titer will be computed, and these fold-rises will be compared between nOPV vs mOPV groups within background vaccination cohort and type using standard *t*-distribution methodology.

Similar comparisons will be made for seroprotection rates, seroconversion rates, and other response rates (any fold-rise, 2-fold rise). For these binary variables, two-sided 95% Miettinen and Nurminen confidence intervals for the rate difference (nOPV minus mOPV) will be computed, and these will be supplemented with a two-sided Fisher exact test p-value.

Imbalance in baseline immunity between sites will be evaluated via the Kruskal-Wallis test for Nab titers. Wherever the global test is significant at two-sided level $\alpha = 0.10$, pairwise comparisons will be drawn from the Wilcoxon test.

11.5.7 Viral Shedding Analysis

Only participants receiving a dose will be evaluated for viral shedding in the corresponding post-dose period. The SAP will provide detail about participants selected into each analysis.

11.5.7.1 Descriptive Analyses

For each group and time point, viral shedding positivity (PCR) and infectivity ($\log_{10}$ $CCID_{50}$ per gram) will be summarized. Additional summary of infectivity will be conducted only among those PCR-positive for (only) the vaccine virus. Positivity will be accompanied by two-sided exact 95% confidence intervals for the proportion, and infectivity will be summarized with the median and bootstrap-based 95% CI. All samples will be evaluated for the appropriate virus type (1 or 3). Participants PCR-positive for type-specific viral shedding of only the appropriate virus but with $\log_{10}$ $CCID_{50}$ per gram equal to LLOQ will contribute a value equal to the LLOQ; participants PCR-negative for viral shedding of the appropriate virus will contribute zero. A summary will also be produced for each post-dose period of the proportion of

participants contributing any PCR-positive sample, any culture-positive sample (≥2.75 $\log_{10}$ $CCID_{50}$ per gram), and those with $\log_{10}$ $CCID_{50}$ per gram ≥4.0)

The Shedding Index Endpoint (SIE) will be computed as the mean of the $\log_{10}$ $CCID_{50}$ per gram from nominal collection days 7, 14, 21, and 28 post-dose (e.g., study days 8, 15, 22, and 29 following the first dose). An additional summary table will describe the samples considered to be within-window for computation of the Shedding Index Endpoint. Permissible stool sample windows will mirror the visit windows defined in this protocol. The SIE will be computed for each participant contributing samples at each time point, and will be summarized by group, including the median and its bootstrap-based 95% confidence intervals. The SAP will define the AUC computations for each post-dose period, which will be summarized similarly.

Time to cessation of shedding is defined as the time (days) between vaccination and last PCR-positive stool prior to 2 consecutive PCR-negative stools (with a minimum 24-hour interval between the 2 negative stools). The time to cessation of shedding will be assessed with interval-censored methodology to describe and compare the duration of shedding. Similarly, supplementary definitions of shedding negativity (culture-negative, defined as the last culture-positive value, and "transmission-negative", defined as the last value with ≥4.0 $\log_{10}$ $CCID_{50}$ per gram) will be described in the SAP, and evaluated with methods accommodating censored data. The methods for considering participants censored will be further defined in the SAP.

Descriptive analysis and plots of the reverse cumulative distribution of the daily infectivity assay results, the SIE, and the AUC will be generated. Additional figures will display the shedding of virus over time.

Additional subgroup analyses may be specified in the SAP.

11.5.7.2 Comparative Analyses

Comparisons of viral shedding data will be made within type and within background vaccination group (exclusively IPV and OPV-containing). Rate of shedders (PCR, culture-positive, etc.) will be compared at each time point using summary statistics, as well as 95% score-based confidence intervals for the difference in proportions at each time point. The $\log_{10}$ $CCID_{50}$ per gram will be compared between groups with a Wilcoxon test for the difference between groups, accompanied by the difference in medians and accompanying bootstrap-based 95% confidence interval, both overall and among only those PCR-positive for (only) the appropriate virus. The same methods will be used for the SIE and AUC.

Tests between nOPV and mOPV for time-to-shedding cessation endpoints will be conducted with two-sided log-rank tests using a Type I error rate of 5%.

11.5.8 Genetic Stability Analyses

As an exploratory evaluation, genetic stability of shed vaccine virus may be evaluated for the potential for neurovirulence in a transgenic mouse model using select post-vaccination stool samples. Briefly, transgenic mice susceptible to poliovirus will be inoculated with virus isolated and cell culture amplified from participant stool samples, and the mice will be monitored and scored for presence/absence of paralysis. The proportion of mice paralyzed as a function of inoculum dose will be used to describe and compare the nOPVs to the corresponding mOPVs, according to OPV type. The assay may include mice inoculated with relevant control material, and/or mice inoculated with the clinical trial material, to compare to the resultant shed virus from participants.

In addition, NGS may be used to explore genetic heterogeneity of shed virus, including reversion at known sites of attenuation as well as attenuating modifications introduced to enhance genetic stability. Sequence information on shed virus may be compared with the results of neurovirulence testing, if available. Both stool suspension and amplified material may be sequenced.

11.5.9 Handling of Dropouts and Missing Data

All missing data will be assumed to be missing completely at random, and no imputation will be performed. Analysis will, therefore, exclude participants with missing or non-evaluable measurements. If an excess of data is missing or if patterns in the missing data are detected, the SAP may specify methods to accommodate this.

12 QUALITY ASSURANCE AND QUALITY CONTROL

Guidance on internal and external processes to assure effective protocol implementation, quality of the research conducted and compliance with PATH and applicable regulatory requirements.

12.1 General Considerations

The study will be conducted in full compliance with the protocol and ICH GCP to provide public assurance that the rights, safety, and well-being of trial participants are protected, and that the clinical trial data are credible. To ensure quality and standardization, the site will develop SOPs for key protocol procedures and conduct the study guided by the study Manual of Procedures or other written guidelines. The site will also develop routine operational checks to verify that critical protocol requirements and procedures are executed correctly and completely at the time the work is being performed. Prior to the initiation of the study, PATH and Emmes will conduct training for the protocol, including applicable SOPs, for study staff.

The investigational site will provide direct access to all study related documents, source data/documents, and reports for the purpose of monitoring and auditing by PATH, and inspection by local and regulatory authorities.

12.2 Study Monitoring

PATH, the sponsor of this study is responsible for ensuring that the study is conducted in accordance with ICH GCP and regulatory requirements. For this purpose, monitors under contract from PATH or its designees will provide external monitoring for this study. A site initiation visit will be conducted prior to beginning the study, and monitoring will be conducted during, and at closeout of the study. During the study, the monitors will visit the clinical site at intervals to verify compliance to the protocol, completeness, accuracy, and consistency of the data, study vaccine accountability, and adherence to ICH GCP and applicable regulations. As needed and when appropriate, the monitors will also provide clarifications and additional training to help the site resolve issues identified during the monitoring visit. As appropriate and informed by risk assessment, remote centralized monitoring activities may be considered in place of or to supplement onsite monitoring. These may include analysis of data quality (e.g., missing or inconsistent data, outlier data), and identifying data trends not easily detected by onsite monitoring and performance metrics (e.g., screening or withdrawal rates, eligibility violations, timeliness, and accuracy of data submission).

The extent and frequency of the monitoring visits will be described in a separate Study Monitoring Plan (SMP) developed prior to study initiation. The investigator will be notified in advance of the scheduled monitoring visit. The monitor should have access to all trial related sites, participant medical records, study vaccine accountability and other study-related records needed to conduct monitoring activities.

PATH is to be notified by the investigator or Emmes in an expedited manner of all detected major protocol deviations. PATH and Emmes will share the findings of the monitoring visit, including any corrective actions, with the site investigator. The site PI and the monitor must agree to cooperate to ensure that any problems detected during these monitoring visits are resolved in a predefined timeframe.

12.3 Independent Auditing

PATH or its designee may audit the study to ensure that study procedures and data collected comply with the protocol and applicable SOPs at the clinical site and that data are correct and complete. The site PIs will permit auditors (employees of PATH or employee of a company designated by PATH) to verify source data validation of the regularly monitored clinical study. The auditors will compare the entries in the eCRFs with the source data and evaluate the study site for its adherence to the clinical study protocol and GCP guidelines and applicable regulatory requirements.

12.4 Regulatory Agency Auditing

The site PIs must be aware that regulatory authorities, including IRB/IEC may wish to inspect the site to verify the validity and integrity of the study data, and protection of human research participants. The site PIs will notify PATH within 24 hours following contact by a regulatory authority. The site PIs must make the relevant records available for inspection and will be available to respond to reasonable requests and audit queries made by authorized representatives of regulatory agencies. The site PIs will provide PATH with copies of all correspondence that may affect the review of the current study or their qualification as an investigator in clinical studies conducted by PATH. PATH will provide any needed assistance in responding to regulatory audits or correspondence.

13 ETHICAL CONSIDERATIONS (AND INFORMED CONSENT)

13.1 Ethical Standards

This study will be conducted in accordance with the ethical principles set forth in The Belmont Report: Ethical Principles and Guidelines for the Protection of Human Subjects of Research, as drafted by the US National Commission for the Protection of Human Subjects of Biomedical and Behavioral Research and in conformity with ICH GCP E6 (R2), 45 CFR Part 46 and 21 CFR 50.

13.2 Ethical Review

Each participating institution will be responsible for assuring that this protocol and the associated ICFs and study-related documents are reviewed and approved by an IRB/IEC prior to implementation of the protocol. Any amendments to the protocol, ICFs, or other study-related documents must be approved by the IRB/IEC and PATH prior to implementation. A copy of the protocol, proposed ICF, other written participant information, and any proposed advertising material will be submitted to each study site's designated IRB/IEC for written approval. The investigator must submit and obtain, as necessary, approval from the IRB/IEC for all subsequent protocol amendments and changes to the ICF. The investigator will notify the IRB/IEC of SAEs as noted in the protocol and of protocol deviations according to local regulatory authority and IRB/IEC requirements. The study will be conducted in full compliance with the protocol.

13.3 Informed Consent Process

Informed consent is a process that is initiated prior to the individual agreeing to participate in the study and continues throughout the individual's study participation. Before any study-related activities and in agreement with applicable regulatory requirements, the site PI must ensure that the participant is fully

informed about the aims, procedures, potential risks, and potential benefits of the study. The participant will be given the written, local IRB/IEC approved ICF, allowed ample time to read the consent form, encouraged to ask questions about the study, have the questions answered and then be given time to decide if they would like to participate in the study. It will be emphasized that participation is voluntary, and that the participant has the right to decline to participate or subsequently withdraw from the study at any time without prejudice.

The site PIs or designees must obtain the participant's voluntary, signed, and dated ICF before any study-related procedures are performed. Study staff must document the informed consent process. The original, signed ICF must be kept in the site study file. A copy of the informed consent document will be given to the participants for their records.

For each participant, following ICF signature, as further evidence that the comprehension requirement of informed consent is fulfilled, an informed consent comprehension assessment will be used in the form of an open-ended questionnaire to assess the participant's recall of information related to study investigational procedures and expectations regarding hygiene.

The informed consent process and the informed consent comprehension assessment may be undertaken using electronic systems and processes, provided designated IRB/IEC approval, and provided that there is adherence to the guidance provided in the FDA document "Use of Electronic Informed Consent. Questions and Answers. Guidance for Institutional Review Boards, Investigators, and Sponsors. December 2016."

13.4 Participant Confidentiality

The investigators, PATH and all staff from organizations involved with the implementation of the trial must ensure that the participant's confidentiality is maintained. Personal identifiers will not be included in any study report. All study records will be kept confidential to the extent provided by national and local laws. Medical records containing identifying information may be made available for review when the study is monitored by PATH or an authorized regulatory agency. Direct access may include examining, analyzing, verifying, and reproducing any records and reports that are important to the evaluation of the study.

When appropriate and to the extent possible, study procedures will be conducted to protect participant privacy and confidentiality.

All study-related information will be stored securely at the study site. All participant information will be stored in locked file cabinets in areas with access limited to study staff. Data collection, process, and administrative forms, laboratory specimens, and other reports will be identified by a coded number only to maintain participant confidentiality. All records that contain names or other personal identifiers, such as locator forms and ICFs, will be stored separately from study records identified by code number. All local databases will be secured with password-protected access systems. Forms, lists, logbooks, appointment books, and any other listings that link Participant ID numbers to other identifying information will be stored in a separate, locked file in an area with limited access. Participants' study information will not be released without their written permission, except as necessary for monitoring.

13.5 Reimbursement

Pending local EC approval, participants will be compensated for their time and effort in this study and be reimbursed for travel to study visits. The study ICF will state the plan for reimbursement. Participants will not be charged for study vaccinations, research clinic visits, research-related examinations, or research-related laboratory tests.

13.6 Risk and Benefits

Participants will not derive any direct benefits from their participation in this research study. Participants may indirectly benefit from the clinical assessments (medical history, physical examination, and routine clinical safety laboratory tests) conducted at screening and during the study. If the participant is found to have any newly diagnosed medical condition or infection, the investigator will ensure that the participant is provided with appropriate and adequate referrals within the health care system. Participation in this study will hopefully contribute to development and eventual deployment of a vaccine to address a global public health concern.

Preclinical testing of the two nOPVs (nOPV1 and nOPV3) to be assessed in this study have not identified specific, inherent risks of these products. There are currently no clinical safety data for nOPV1 or nOPV3 as they have not yet been tested in humans; however, a similar nOPV, against type 2 poliovirus (nOPV2), has been assessed in phase 1 and 2 trials in adults in Belgium and in a phase 2 trial in young children and infants in Panama, and the vaccine has been well-tolerated and no specific safety concerns have been identified.

As the nOPVs are derived from Sabin OPV, the risks associated with Sabin OPV must be considered possible for these products. There are certain symptoms that have commonly been associated with immunizations in general, and if they do occur, are usually not severe in intensity and transient in nature. Consequently, in early clinical trials of investigational vaccines, these symptoms are typically considered as risks and are solicited as potential AEs in the first week following immunization. As with other similar vaccines, these events have also been observed following immunization with Sabin OPV; therefore, in this study, the AEs that will be actively solicited in the one week following administration of the study vaccines include such events, specifically, fever, chills, fatigue, headache, myalgias (muscle aches), arthralgias (joint aches), nausea, vomiting, abdominal pain, and diarrhea.

The most concerning risks for the Sabin OPVs are VAPP and the introduction of VDPV into vulnerable populations. VAPP, in which vaccine administered to an individual reverts to virulence (a risk the nOPVs are designed to avoid) and results in paralytic polio in that individual, occurs in approximately 1 in every 2.4 million doses distributed, overall, and approximately 1 case in every 750,000 doses among those receiving the first dose. More specifically, participants will have had the full recommended polio vaccination series, with confirmation of protective levels of antibodies against types 1 and 3 poliovirus, and will also be assessed for immunocompetence, further greatly reducing the prospects of this generally very rare risk of Sabin OPV. And for further risk mitigation, to protect against provocation poliomyelitis, participants will be prohibited from the receipt of any intramuscular injection for 30 days, prior to or following each study vaccination (except seasonal inactivated influenza and COVID-19 vaccines, which are prohibited for 14 days).

Risks associated with contamination with shed vaccine virus, and potential transmission to others, is to be mitigated by emphasis on strict hygienic practices for the vaccine recipient, at least until cessation of shedding of vaccine virus by the participant is confirmed. As components of such strict hygienic practices, study participants will be counseled to:

- flush toilet with toilet lid closed, and then sanitize the toilet surfaces
- use hygienic techniques to obtain and handle stool specimens
- wash hands after toilet use
- wash hands before handling food
- shower prior to entering public swimming pools and avoid spas and hot tubs

Individuals who may be involved with the professional handling of food, catering or food production activities during study participation will not be eligible to participate in the study.

Individuals who during their study participation are likely to come in direct household or close professional contact with those who are either immunocompromised, pregnant, encopretic, less than two years of age, or who have not received a full polio vaccine primary immunization series, will be excluded from the study, and study participants will be reminded to avoid contact with such people, at least until it is confirmed that they have ceased to shed vaccine virus. Close professional contact means that the person works in close physical proximity for routine, sustained time periods with the person at risk.

Study clinics will be requested to schedule participant study visits and their flow through the clinic, insofar as is possible, to avoid direct contact between participants participating in this study. Participants in the study will be advised to notify their primary caregivers and any health care provider who were to attend to the participant about their participation in the study. The contact information for the study will be provided to the healthcare provider to allow them to obtain additional information on the study.

Risks of vaccine virus transmission to study clinic staff will be mitigated by ensuring that all site staff in contact with participants or their stool specimens have been fully immunized against polio and they will be required to adhere to the relevant CDC guidelines for the prevention of transmission of poliovirus in healthcare settings, included under both standard and contact precautions [12]. The site laboratory processing the stool specimens must meet BSL-2 requirements. Details as to how stool specimens should be collected, managed, and processed on site, as well as the use of personal protective equipment, incorporating these precautions, will be provided in the study-specific Laboratory Manual.

Hypersensitivity reactions may occur following the administration of any vaccine, including licensed vaccines, which in rare circumstances may be life-threatening. Investigators are informed in the protocol and IB of this possibility following study vaccine administration. All participants shall be informed in the ICF and observed for a minimum of 30 minutes following study vaccine administration. As with all immunizations, appropriate emergency medical treatment shall be made available in case of severe immediate reactions, such as anaphylaxis. Participants with a known hypersensitivity to any component of the study vaccines will be excluded from the study, or if hypersensitivity is observed after administration of initial vaccination, the participant will be excluded from any further study vaccination.

Blood drawing and venipuncture associated risks may include minor bleeding or bruising at the venous access site, mild discomfort, upset stomach, dizziness, light-headedness, syncope, or very rarely infection. Blood samples will only be drawn by trained staff members using aseptic technique and medical assistance will be available in case of any complications. Participants will be informed of risks in the ICF and will be in a seated or supine position during blood draws.

The known risks of participation in this study are believed to be outweighed by the value of the information to be gained.

13.7 Reporting to Local Health Department

Each study site will be required to notify the local health department that oversees their catchment area of the proposed implementation of this study, and any further guidance and precautions mandated by them will be followed.

13.8 Compensation for Research Related Injury

Participants will be instructed that if they experience any illness during the study, they should contact study staff as soon as possible. Participants will be encouraged to be evaluated at the study clinic, if

possible. If participants seek medical care outside the study clinic, they should inform the health care provider(s) of their participation in this study, provide the health care provider(s) with contact information for the investigator, and separately contact the investigator to inform the investigator of the medically attended illness. There are limited funds available to the study for care of study-related injury, in addition to modest no-fault insurance for study-related injury. In addition, both PATH, the Sponsor, and Bio Farma, the vaccine manufacturer, will have liability insurance coverage for the trial. Although the investigators and PATH will make every effort to cover the costs of any study-related injury, full coverage cannot be guaranteed, and uncovered costs may fall on participants and/or their insurers. Participants will be reimbursed to the extent possible for costs of medical care necessary for any study-related injury. Participants will be informed of the limitations for coverage of study-related care costs and that there is the possibility that they may ultimately be responsible for at least some of the cost of care.

14 FINANCING AND INSURANCE

The trial is supported by a grant from the Bill and Melinda Gates Foundation to PATH. Funding of the sites and Emmes' activities will also be covered by this grant through PATH. Bio Farma will be providing pharmaceutical support through the provision of the clinical study vaccines.

PATH will secure insurance that complies with the applicable regulatory and legal requirements.

15 PUBLICATION POLICY

According to the policy of the International Committee of Medical Journal Editors (ICMJE) member journals, this clinical trial will be registered in a public trials registry such as ClinicalTrials.gov, which is sponsored by the National Library of Medicine in accordance with their requirements under the Food and Drug Administration Amendments Act.

The information generated in this study will be used by PATH in connection with the development of the product and therefore may be disclosed to government regulatory agencies in various countries, as well as global public health organizations, such as the World Health Organization. PATH (or designee) will prepare a CSR according to ICH-E3 guidelines. PATH and Bio Farma recognize the importance of communicating study findings and will therefore encourage their publication in reputable scientific journals and presentation at seminars or conferences, while protecting the integrity of the ongoing trial. Study results will be made publicly available in compliance with the WHO mandated timeframe for public disclosure of results from clinical trials. Any publication, lecture, manuscripts of the findings of this study by any individual involved with the study will be governed by the procedure outlined in the Clinical Trial Agreement. The ICMJE authorship criteria will be strictly followed for publication of any manuscripts arising from this trial. Within any presentation or publication, confidentiality of individual participants will be maintained, with identification by participant code number and initials, if applicable.

16 REFERENCES

[1] GPEI-cVDPV-factsheet: http://polioeradication.org/wp-content/uploads/2018/07/GPEI-cVDPV-Fact-Sheet-20191115.pdf

[2] WHO. Wkly Epidemiol Rec. No 46, 2019, 94, 525–540

[3] Yeh MT, Bujaki E, Dolan PT, Smith M, Wahid R, Konz J, et al. Engineering the Live-Attenuated Polio Vaccine to Prevent Reversion to Virulence [published online ahead of print, 2020 Apr 10]. Cell Host Microbe. 2020; S1931-3128(20)30230-4

[4] Singer C, Knauert F, Bushar G, Klutch M, Lundquist R, Quinnan GV Jr. Quantitation of poliovirus antigens in inactivated viral vaccines by enzyme-linked immunosorbent assay using animal sera and monoclonal antibodies. J Biol Stand. 1989; 17:137–50

[5] Minor P.D., Ferguson M., Katrak K., Wood D., John A., Howlett J., et al. Antigenic structure of chimeras of type 1 and type 3 polioviruses involving antigenic sites 2, 3, and 4. J. Gen Virol. 1991 Oct; 72:2475-81

[6] Minor P.D., Macadam, A.J. Personal communication on monoclonal antibodies for type 3 available at NIBSC

[7] Ohka S, Igarashi H, Nagata N, Sakai M, Koike S, Nochi T, et al. Establishment of a poliovirus oral infection system in human poliovirus receptor-expressing transgenic mice that are deficient in alpha/beta interferon receptor. J Virol. 2007 Aug; 81(15):7902-12

[8] Dragunsky EM, Ivanov AP, Wells VR, Ivshina AV, Rezapkin GV, Abe S, et al. Evaluation of immunogenicity and protective properties of inactivated poliovirus vaccines: a new surrogate method for predicting vaccine efficacy. J Infect Dis. 2004 Oct 15; 190(8):1404-12

[9] Van Damme P, De Coster I, Bandyopadhyay AS, Revets H, Withanage K, De Smedt P, et al. The safety and immunogenicity of two novel live attenuated monovalent (serotype 2) oral poliovirus vaccines in healthy adults: a double-blind, single-centre phase 1 study. Lancet. 2019; 394(10193):148-158

[10] Caceres VM, Sutter RW. Sabin monovalent oral polio vaccines: review of past experiences and their potential use after polio eradication. Clin Infect Dis. 2001 Aug 15; 33(4):531-41

[11] Eggers M, Terletskaia-Ladwig E, Rabenau HF, Doerr HW, Diedrich S, Enders G, et al. Immunity status of adults and children against poliomyelitis virus type 1 strains CHAT and Sabin (LSc-2ab) in Germany. BMC Infectious Diseases. 2010 Dec 9; 10:347

[12] Siegel JD, Rhinehart E, Jackson M, Chiarello L, and the Healthcare Infection Control Practices Advisory Committee. 2007 Guideline for Isolation Precautions: Preventing Transmission of Infectious Agents in Healthcare Settings https://www.cdc.gov/infectioncontrol/guidelines/isolation/index.html

APPENDICES

APPENDIX 1: SCHEDULE OF STUDY VISITS AND EVALUATIONS

Participants with an Exclusive IPV Vaccination History (Cohorts 1 and 3: Single-dose groups)														
**Procedure**	Screen	Visits												
Visit Name	00	01	01A	01B	02	02A	02B	02C	03	03A	03B	03C	03D	04
Study Day (allowed window days)	-90 to 1	1	3	5	8 (+1)	10	15	22	29 (+2)	36	43	50	57	169 (+14)
Clinic visits	X	X			X				X					
Stool collection			X	X	X#	X#	X#	X#	X#	X	X	X	X[1]	
Allowed stool collection window (days)			±1	±1	D8±1	±1	±2	±2	D29±3	±1	±2	±2	±3	
Informed consent	X													
Eligibility criteria	X	X												
Demographic data	X													
Medical history	X													
Concomitant medication	X									X[2]				
Full physical exam (PE) with VS	X													
Vital signs (VS)		X												
Targeted PE with VS, if indicated		X			X				X					X[4]
Blood for HIV1/2, HBsAg, HCV Ab, total IgG & IgA, pregnancy in FOCP	X													
Blood for CBC & chemistry	X	X[3]			X									
Blood for type-specific poliovirus neutralizing Ab	X								X					
Urine pregnancy in FOCP		X												
Randomization		X[5]												
Study vaccine		X												
Collect solicited AEs		X												
Collect unsolicited AEs		X												
Collect SAEs		X												

[#] – Participants may contribute additional stool samples between study Days 8 and 28, in addition to the nominal study stool sampling days, if available.
X[1] – If cessation of vaccine virus shedding not confirmed, collections will continue until cessation confirmed
X[2] – Only those associated with SAEs or COVID-19 vaccinations
X[3] – The results of these laboratory tests are not required for eligibility, but will be used as baseline for analyses
X[4] – Day 169 assessments will be performed via telephone. The participants will only come to the clinical if a physical exam is indicated.
X[5]– Randomization can be done prior to Day 1 after confirmation of eligibility.

Participants with an OPV-Containing Vaccination History (Cohorts 2 and 4: Two-dose groups)											
**Procedure**	Screen	Visits									
Visit Name	00	01	02	02A	02B	03	04	04A	04B	05	06
Study Day (allowed window days)	-90 to 1	1	8 (+1)	15	22	29 (+2)	36 (+1)	43	50	57 (+2)	169 (+14)
Clinic visits	X	X	X			X	X			X	
Stool collection			X	X	X	X	X	X	X	X[1]	
Allowed stool collection window (days)			D8±1	±2	±2	D29±3	D36±1	±2	±2	D57±3	
Informed consent	X										
Eligibility criteria	X	X				X					
Demographic data	X										
Medical history	X										
Concomitant medication	X										X[2]
Full physical exam (PE) with VS	X										
Vital Signs (VS)		X				X					
Targeted PE with VS, if indicated		X	X			X	X			X	X[4]
Blood for HIV1/2, HBsAg, HCV Ab, total IgG & IgA, pregnancy in FOCP	X										
Blood for CBC & chemistry	X	X[3]	X								
Blood for type-specific poliovirus neutralizing Ab	X					X				X	
Urine pregnancy in FOCP		X				X					
Randomization		X[5]									
Study vaccine		X				X					
Collect solicited AEs		X				X					
Collect unsolicited AEs		X									
Collect SAEs		X									

X[1] – If cessation of vaccine virus shedding not confirmed, collections will continue until cessation confirmed
X[2] – Only those associated with SAEs or COVID-19 vaccinations
X[3] – The results of these laboratory tests are not required for eligibility, but will be used as baseline for analyses
X[4] – Day 169 assessments will be performed via telephone. The participants will only come to the clinical if a physical exam is indicated.
X[5] – Randomization can be done prior to Day 1 after confirmation of eligibility.
